# Advanced optimal control approaches for immune boosting and clinical treatment to enhance dengue viremia models using ABC fractional-order analysis

**DOI:** 10.3389/fpubh.2024.1398325

**Published:** 2024-10-21

**Authors:** G. M. Vijayalakshmi, M. Ariyanatchi, Lenka Cepova, Krishnasamy Karthik

**Affiliations:** ^1^Department of Mathematics, Vel Tech Rangarajan Dr. Sagunthala R&D Institute of Science and Technology, Chennai, Tamil Nadu, India; ^2^Department of Machining, Assembly and Engineering Metrology, Faculty of Mechanical Engineering, VSB-Technical University of Ostrava, Ostrava, Czechia; ^3^Department of Mechanical Engineering, Vel Tech Rangarajan Dr. Sagunthala R&D Institute of Science and Technology, Chennai, Tamil Nadu, India

**Keywords:** dengue fractional-order mathematical modeling, Atangana-Baleanu operator, Lyapunov stability, basic reproduction value, optimal control, Adams-Bashforth method

## Abstract

**Introduction:**

This work focuses on the Dengue-viremia ABC (Atangana-Baleanu Caputo) fractional-order differential equations, accounting for both symptomatic and asymptomatic infected cases. Symptomatic cases are characterized by higher viremia levels, whereas asymptomatic cases exhibit lower viremia levels. The fractional-order model highlights memory effects and other advantages over traditional models, offering a more comprehensive representation of dengue dynamics.

**Methods:**

The total population is divided into four compartments: susceptible, asymptomatic infected, symptomatic infected, and recovered. The model incorporates an immune-boosting factor for asymptomatic infected individuals and clinical treatment for symptomatic cases. Positivity and boundedness of the model are validated, and both local and global stability analyses are performed. The novel Adams-Bash numerical scheme is utilized for simulations to rigorously assess the impact of optimal control interventions.

**Results:**

The results demonstrate the effectiveness of the proposed control strategies. The reproduction numbers must be reduced based on specific optimal control conditions to effectively mitigate disease outbreaks. Numerical simulations confirm that the optimal control measures can significantly reduce the spread of the disease.

**Discussion:**

This research advances the understanding of Dengue-viremia dynamics and provides valuable insights into the application of ABC fractional-order analysis. By incorporating immune-boosting and clinical treatment into the model, the study offers practical guidelines for implementing successful disease control strategies. The findings highlight the potential of using optimal control techniques in public health interventions to manage disease outbreaks more effectively.

## 1 Introduction

Worldwide, thousands of dengue cases are reported every year. The world's tropical and subtropical regions are affected by dengue infection, which is a mosquito-carrying disease. A high temperature and flu-like symptoms are signs of mild illness or asymptomatic to stern disease. DHF (Dengue Hemorrhagic Fever) or DSS (Dengue Show Syndromes Syndromes) is a highly infectious form of dengue fever that causes serious bleeding, shock, and death. Generally, it was noticed that only one out of four dengue contagions is symptomatic. Dengue virus occurs in four major types (DENV types 1, 2, 3, and 4), all of which can cause serious illness. The usual signs of DENV type 1 are like a common cold and mild fever, which will not lead directly to DHF; conversely, later DENV types can lead to DHF (1–3).

To understand the dynamical behavior of dengue transmission, we formulated a mathematical model, particularly focusing on vector-borne disease transmission from mosquitoes to humans. Esteva and Vargas (4, 5) pioneered the creation of a fundamental dengue model and explored numerous fundamental mathematical concepts and their accompanying numerical simulations. Feng et al. (6) presented a two-strain dengue infection model and examined competitive exclusion. Researchers have conducted numerous studies to better understand the transmission of dengue fever (7–9).

The importance of fractional-order models lies in their ability to capture the complex dynamics and long-term dependencies within the transmission process. By incorporating fractional derivatives, these models provide a more comprehensive understanding of disease spread, which is crucial for designing effective intervention strategies. The fractional-order models can accommodate the nuanced behavior of dengue transmission, offering insights that integer-order models may overlook, thereby enhancing the accuracy and effectiveness of disease control measures.

The fractional order model has been conclusively demonstrated by a recent study to be capable of controlling the trend of complex diffusion disorder (10–14). Many have emphasized various mathematical models for Dengue transmission and prevention (15–19). All cited references explain the transmission process of Dengue infection from different perspectives, including dynamic analysis, evaluation of vaccination, and optimal control measures (20–24). The most updated studies on Dengue with real-life data are presented in (25, 26). The mathematical description of Dengue is briefly described in Deterministic and Stochastic terms. The evolution of dengue with asymptomatic carriers using optimal control measures was investigated in (27).

Therefore, motivated by the aforementioned literature, we propose a computational framework for the dissemination of dengue at a given viremia level. We investigated whether symptom-free people were markedly more susceptible to mosquitoes than clinically symptom-positive patients. The new idea of a mathematical model to analyse the immune-boosting factor for asymptomatic infected cases and the waning immunity that cases re-infect is reported. To make practical applications and simulations easier, we utilize the Adams-Bash forth numerical scheme, which is renowned for its accuracy and stability. This choice ensures that our model reflects real-world scenarios while maintaining computational efficacy. A key highlight of this study is the incorporation of optimal control strategies into the ABC fractional order Dengue viremia model. These strategies are designed to explore how interventions, such as self-prevention and vector control, can be optimized to curtail disease spread. The analysis extends to investigating disease-free and endemic stability, providing crucial insights into the long-term behavior of the system under various control scenarios.

This article is prepared as follows: In portion 2, we review the fundamental definitions for the fractional-order operator and provide a list of mathematical properties that were used throughout the work. The dengue viral mathematical model with fractional order was presented in portion 3. Portion 4 examines the local as well as global consistency of the suggested model through the Routh-Hurwitz criteria and the Lyapunov function. An optimal control solution and discussion are present in portion 5. The final section focuses on numerical simulations and a comprehensive conclusion.

## 2 Fundamental results

This section introduces fractional derivation and some of its properties, which will be used in the following components.

**Definition 2.1**.

Consider ψ ∈ ℍ′ (0, *T*) and η ∈ [0, ȶ], then Atangana-Baleanu fraction component in Caputo case is


(1) $$\begin{array}{l} \begin{array}{c} AℬC\\ 0 \end{array}{𝔇}_{ȶ}^{\eta }\psi \;\left(ȶ\right)\;=\;\frac{\bar{N\left(\eta \right)}}{1-\;\eta }\int_{0}^{ȶ}\frac{d}{dx}\;\psi \left(S\right)N_{\eta }\left[-\frac{\eta }{1-\;\eta }\left(ȶ-S\right)\right]dS \end{array}$$

The method yields a variation operator Caputo-Fabrizio that replaces


$$\begin{array}{l} N_{\eta }\left[-\frac{\eta }{1-\;\eta }\left(ȶ-S\right)\right]dS\text{ by }{\;N}_{1}=\;exp\left[-\frac{\eta }{1-\;\eta }\left(ȶ-S\right)\;\right] \end{array}$$

It's noteworthy that $\begin{array}{c} AℬC\\ 0 \end{array}{𝔇}_{ȶ}^{\eta }$[constant] = 0. Here $\bar{N\left(\eta \right)}$ is the typical function and it is defined as $\bar{N\left(0\right)}=1$ and $\bar{N\left(1\right)}=1$. $\bar{\;N\left(\eta \right)}$ depict the familiar Mittag–Leffler operator, it also reflects the exponential function generality.

**Definition 2.2**.

The fractional integral of ABC with order η given by


(2) $$\begin{array}{l} \begin{array}{c} AℬC\\ 0 \end{array}{𝔗}_{ȶ}^{\eta }\psi \left(ȶ\right)=\;\frac{1-\;\eta }{\bar{N\left(\eta \right)}}\;\psi \left(ȶ\right)\;+\;\frac{\eta }{\bar{N\left(\eta \right)\;\Gamma \left(\eta \right)\;}}\int_{0}^{ȶ}\;\psi \left(S\right){\left(ȶ-S\right)}^{\eta \;-1}dS \end{array}$$

**Lemma 2.1**.

Consider a fractional-order system


(3) $$\begin{array}{l} \begin{array}{c} AℬC\\ 0 \end{array}{𝔇}_{ȶ}^{\eta }x\left(ȶ\right)=f\left(ȶ,x\right),\;t>t_{0} \end{array}$$

Where ηϵ(0, 1) in the initial case $x\left({ȶ}_{0}\right).$

If f(ȶ,x) fulfills the Lipschitz condition in relation to *x*, then system (Equation 3) exhibits a unique solution in the region [*t*_0_, +∞) × φ and φ ⊆ ℝ^*n*^.

**Lemma 2.2**.

If $x\left(ȶ\right)\in {\mathbb{R}}^{+}$ become an ongoing and attainable consequence. Then


(4) $$\begin{array}{l} \begin{array}{c} AℬC\\ 0 \end{array}{𝔇}_{ȶ}^{\eta }\left(x\left(ȶ\right)-x^{*}-x^{*}\ln\frac{x\left(ȶ\right)}{x^{*}}\right)\;\leq \left(1-\frac{x^{*}}{x\left(ȶ\right)}\right)\begin{array}{c} AℬC\\ 0 \end{array}{𝔇}_{ȶ}^{\eta }x\left(ȶ\right) \end{array}$$

Here *t* > *t*_0_, ηϵ(0, 1) and $x^{*}\in \;{\mathbb{R}}^{+}.$

## 3 Evaluation of dengue dynamics

In this section, we expand upon the previously described Dengue SIR-SI model (18) by incorporating additional factors and refining the classification of both human and mosquito populations. Our model includes viremia levels, an immune-boosting factor for asymptomatic infected cases, and clinical treatment for symptomatic infected cases.

**Figure 1:**
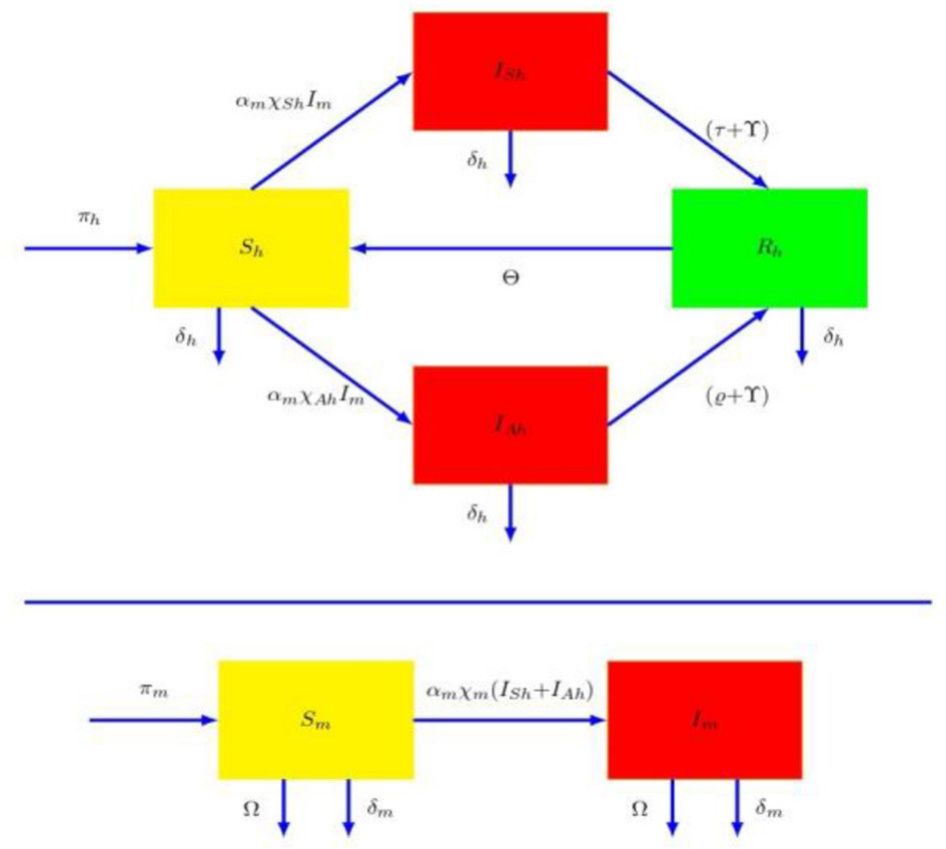
The process diagram in dengue dynamics.

To study the mode of spread of dengue sickness, the human species ($N_{𝔥}$) is subdivided into four classes: susceptible (Ȿ_𝔥_), symptomatic infectious (𝔗_Ȿ_𝔥__), asymptomatic infectious $\left({𝔗}_{A_{𝔥}}\right)$ and recovered human populations (𝔎_𝔥_). We classified female mosquito species $\left(N_{𝔪}\right)$ into Susceptible (Ȿ_𝔪_) and infective mosquitoes (𝔗_𝔪_). A Susceptible individual among as one who is not infected and immune, infected humans are both asymptomatic and symptomatic are those who have acquired Dengue viremia from an infected mosquito populations and are all capable of spreading dengue virus to susceptible mosquitoes. Let we examines π_𝔥_ and π_𝔪_ the acquisition rates of humans and mosquitoes. The proposed model, illustrated in the flowchart, demonstrates the dengue transmission dynamics. Based on Figure 1, we developed the following differential equation.


(5) $$\begin{array}{l} \frac{d{Ȿ}_{𝔥}}{dȶ}=\pi_{𝔥}\;-\alpha_{𝔪}\left(\chi_{{Ȿ}_{𝔥}}+\chi_{A_{𝔥}}\right){Ȿ}_{𝔥}{𝔗}_{𝔪}\;-\;\delta_{𝔥}{Ȿ}_{𝔥}+\theta {𝔎}_{𝔥}\\ \frac{d{𝔗}_{{\text{s}}_{\text{h}}}}{dȶ}=\;\alpha_{𝔪}\chi_{{Ȿ}_{𝔥}}{Ȿ}_{𝔥}{𝔗}_{𝔪}\;-\;\left(\tau +\gamma +\delta_{𝔥}\right){𝔗}_{{Ȿ}_{𝔥}}\\ \frac{{d𝔗}_{A_{𝔥}}}{dȶ}=\;\alpha_{𝔪}\chi_{A_{𝔥}}{Ȿ}_{𝔥}{𝔗}_{𝔪}\;-\;\left(\varrho +\gamma +\delta_{𝔥}\right){𝔗}_{A_{𝔥}}\;\\ \frac{d{𝔎}_{𝔥}}{dȶ}=\;\gamma \left({𝔗}_{{Ȿ}_{𝔥}}+{𝔗}_{A_{𝔥}}\right)-\theta {𝔎}_{𝔥}-\delta_{𝔥}{𝔎}_{𝔥}+\tau {𝔗}_{{Ȿ}_{𝔥}}+\varrho {𝔗}_{A_{𝔥}}\;\\ \frac{{dⱾ}_{𝔪}}{dȶ}=\pi_{𝔪}\;-\alpha_{𝔪}\chi_{𝔪}\left({𝔗}_{{Ȿ}_{𝔥}}+{𝔗}_{A_{𝔥}}\right){Ȿ}_{𝔪}-\delta_{𝔪}{Ȿ}_{𝔪}\;\\ \frac{{d𝔗}_{𝔪}}{dȶ}=\;\alpha_{𝔪}\chi_{𝔪}\left({𝔗}_{{Ȿ}_{𝔥}}+{𝔗}_{A_{𝔥}}\right){Ȿ}_{𝔪}-\delta_{𝔪}{𝔗}_{𝔪} \end{array}$$

Where

α_𝔪_- individual mosquito‘s biting rate

χ_Ȿ_𝔥__- Dissemination to human by mosquitoes, which leads to a symptomatic infectious in humans

$\chi_{A_{𝔥}}$- Dissemination to human by mosquitoes, which leads to a asymptomatic infectious in humans

χ_𝔪_- Viremia dissemination to mosquito by human species

δ_𝔥_- Human Fatality rate

τ - Symptomatic infected human treatment rate

γ - Recovering rate.

θ - Transition rate at which a recovered person becomes defenseless due to loss of immunity

ϱ−*Rates of immunosuppression for asymptomatic victims*

δ_𝔪_- Rate of mosquito natural mortality (an average mosquito life span)

From the basic cases $({Ȿ}_{h}\;,\;T_{{Ȿ}_{h}},\;T_{A_{h}},K_{h},{Ȿ}_{m},\;T_{m})\geq 0.$

In this approach, the aggregate human and mosquito population ratios are provided by


$$\begin{array}{l} N_{𝔥}={Ȿ}_{𝔥}+\;{𝔗}_{{Ȿ}_{𝔥}}+\;{𝔗}_{A_{𝔥}}+{𝔎}_{𝔥}\text{ and }{\;N}_{𝔪}={Ȿ}_{𝔪}+\;{𝔗}_{𝔪}. \end{array}$$

In addition, the area of biologically significance for the aforementioned dengue model is indicated and presented by the covered set


$$\begin{array}{l} \Phi =\{{Ȿ}_{𝔥}\;,\;{𝔗}_{{Ȿ}_{𝔥}},\;{𝔗}_{A_{𝔥}},{𝔎}_{𝔥},{Ȿ}_{𝔪},\;{𝔗}_{𝔪}\in \;{\mathbb{R}}_{+}^{6}:{Ȿ}_{𝔥}+{𝔗}_{{Ȿ}_{𝔥}}+\;{𝔗}_{A_{𝔥}}+{𝔎}_{𝔥}\\ \leq N_{𝔥},{Ȿ}_{𝔪}+{𝔗}_{𝔪}\leq N_{𝔪}\}\; \end{array}$$

A fractional representation of the 𝔄*𝔅ℭ* model as


(6) $$\begin{array}{l} \begin{array}{c} AℬC\\ 0 \end{array}{𝔇}_{ȶ}^{\eta }{Ȿ}_{𝔥}=\pi_{𝔥}\;-\alpha_{𝔪}\left(\chi_{{Ȿ}_{𝔥}}+\chi_{A_{𝔥}}\right){Ȿ}_{𝔥}{𝔗}_{𝔪}\;-\delta_{𝔥}{Ȿ}_{𝔥}+\theta {𝔎}_{𝔥}\;\\ \begin{array}{c} AℬC\\ 0 \end{array}{𝔇}_{ȶ}^{\eta }{𝔗}_{{Ȿ}_{𝔥}}=\alpha_{𝔪}\chi_{{Ȿ}_{𝔥}}{Ȿ}_{𝔥}{𝔗}_{𝔪}\;-\;\left(\tau +\gamma +\delta_{𝔥}\right){𝔗}_{{Ȿ}_{𝔥}}\;\\ \begin{array}{c} AℬC\\ 0 \end{array}{𝔇}_{ȶ}^{\eta }{𝔗}_{A_{𝔥}}=\;\alpha_{𝔪}\chi_{A_{𝔥}}{Ȿ}_{𝔥}{𝔗}_{𝔪}\;-\;\left(\varrho +\gamma +\delta_{𝔥}\right){𝔗}_{A_{𝔥}}\;\\ \begin{array}{c} AℬC\\ 0 \end{array}{𝔇}_{ȶ}^{\eta }{𝔎}_{𝔥}\;=\;\gamma \left({𝔗}_{{Ȿ}_{𝔥}}+{𝔗}_{A_{𝔥}}\right)-\theta {𝔎}_{𝔥}-\delta_{𝔥}{𝔎}_{𝔥}+\tau {𝔗}_{{Ȿ}_{𝔥}}+\varrho {\;𝔗}_{A_{𝔥}}\;\\ \begin{array}{c} AℬC\\ 0 \end{array}{𝔇}_{ȶ}^{\eta }{Ȿ}_{𝔪}=\pi_{𝔪}\;-\;\alpha_{𝔪}\;\chi_{𝔪}\left({𝔗}_{{Ȿ}_{𝔥}}+{\;𝔗}_{A_{𝔥}}\right){Ȿ}_{𝔪}-\delta_{𝔪}{Ȿ}_{𝔪}\;\\ \begin{array}{c} AℬC\\ 0 \end{array}{𝔇}_{ȶ}^{\eta }{𝔗}_{𝔪}=\;\alpha_{𝔪}\chi_{𝔪}\left({𝔗}_{{Ȿ}_{𝔥}}+{\;𝔗}_{A_{𝔥}}\right){Ȿ}_{𝔪}-\delta_{𝔪}{𝔗}_{𝔪}\; \end{array}$$

A fractional derivation of Atangana-Baleanu of order 0 < η < 1 is denoted $\begin{array}{c} AℬC\\ 0 \end{array}{𝔇}_{ȶ}^{\eta }$ in Caputo notation.

## 4 Model analysis

This section examines the validity, singularity and positive variance of the solution of the SIR-SI type model. Additionally, a reliability estimate for Model (Equation 6) has also been developed.

### 4.1 Existence and uniqueness

**Theorem 4.1**.

For each non- negative initial stage $({Ȿ}_{𝔥}\left(0\right),\;{𝔗}_{{Ȿ}_{𝔥}}\left(0\right),\;{\;𝔗}_{A_{𝔥}}\left(0\right)$, ${𝔎}_{𝔥}\left(0\right),{Ȿ}_{𝔪}\left(0\right),\;{𝔗}_{𝔪}\left(0\right))\in {\mathbb{R}}_{+}^{6},$ then there survives a oneness solution of fractional order model (Equation 6).

**Proof**

Let $\Phi =\{{Ȿ}_{𝔥}\;,\;{𝔗}_{{Ȿ}_{𝔥}},\;{\;𝔗}_{A_{𝔥}},{𝔎}_{𝔥},{Ȿ}_{𝔪},\;{𝔗}_{𝔪}\in \;{\mathbb{R}}_{+}^{6}:\max(|{Ȿ}_{𝔥}|$, $\left|{𝔗}_{{Ȿ}_{𝔥}}|,|{\;𝔗}_{A_{𝔥}}|,|{𝔎}_{𝔥}|,|{Ȿ}_{𝔪}|,|{𝔗}_{𝔪}|)\leq \varepsilon \;\right\}$.

Define a mapping

$𝕄\left(x\right)=\left({𝕄}_{1}\left(x\right),{𝕄}_{2}\left(x\right),{𝕄}_{3}\left(x\right),{𝕄}_{4}\left(x\right),{𝕄}_{5}\left(x\right){,𝕄}_{6}\left(x\right)\right)$ and


$$\begin{array}{l} {𝕄}_{1}\left(x\right)=\pi_{𝔥}\;-\alpha_{𝔪}\left(\chi_{{Ȿ}_{𝔥}}+\chi_{A_{𝔥}}\right){Ȿ}_{𝔥}{𝔗}_{𝔪}\;-\delta_{𝔥}{Ȿ}_{𝔥}+\theta {𝔎}_{𝔥}\;\\ {𝕄}_{2}\left(x\right)=\alpha_{𝔪}\chi_{{Ȿ}_{𝔥}}{Ȿ}_{𝔥}{𝔗}_{𝔪}\;-\;\left(\tau +\gamma +\delta_{𝔥}\right){𝔗}_{{Ȿ}_{𝔥}}\;\\ {𝕄}_{3}\left(x\right)=\alpha_{𝔪}\chi_{A_{𝔥}}{Ȿ}_{𝔥}{𝔗}_{𝔪}\;-\;\left(\varrho +\gamma +\delta_{𝔥}\right){𝔗}_{A_{𝔥}}\;\\ {𝕄}_{4}\left(x\right)=\gamma \left({𝔗}_{{Ȿ}_{𝔥}}+{\;𝔗}_{A_{𝔥}}\right)-\theta {𝔎}_{𝔥}-\delta_{𝔥}{𝔎}_{𝔥}+\tau {\;𝔗}_{{Ȿ}_{𝔥}}+\varrho {\;𝔗}_{A_{𝔥}}\;\\ {𝕄}_{5}\left(x\right)=\pi_{𝔪}\;-\alpha_{𝔪}\chi_{𝔪}\left({𝔗}_{{Ȿ}_{𝔥}}+{\;𝔗}_{A_{𝔥}}\right){Ȿ}_{𝔪}-\delta_{𝔪}{Ȿ}_{𝔪}\;\\ {𝕄}_{6}\left(x\right)=\alpha_{𝔪}\chi_{𝔪}\left({𝔗}_{{Ȿ}_{𝔥}}+{\;𝔗}_{A_{𝔥}}\right){Ȿ}_{𝔪}-\delta_{𝔪}{𝔗}_{𝔪}\; \end{array}$$

Where $x=\left({Ȿ}_{𝔥}\;,\;{𝔗}_{{Ȿ}_{𝔥}},\;{\;𝔗}_{A_{𝔥}},{𝔎}_{𝔥},{Ȿ}_{𝔪},\;{𝔗}_{𝔪}\right)\in \;\Phi$

For any $x,\bar{x}\;\in \;\Phi ,$ we have


$$\begin{array}{l} \left||𝕄\left(x\right)-\bar{𝕄\left(x\right)}||=|{𝕄}_{1}\left(x\right)-\bar{{𝕄}_{1}\left(x\right)}|+|{𝕄}_{2}\left(x\right)-\bar{{𝕄}_{2}\left(x\right)}\right|\\ +|{𝕄}_{3}\left(x\right)-\bar{{𝕄}_{3}\left(x\right)}|+|{𝕄}_{4}\left(x\right)-\bar{{𝕄}_{4}\left(x\right)}|\\ +|{𝕄}_{5}\left(x\right)-\bar{{𝕄}_{5}\left(x\right)}|+|{𝕄}_{6}\left(x\right)-\bar{{𝕄}_{6}\left(x\right)}|\\ \leq |\pi_{𝔥}\;-\alpha_{𝔪}\left(\chi_{{Ȿ}_{𝔥}}+\chi_{A_{𝔥}}\right){Ȿ}_{𝔥}{𝔗}_{𝔪}\;-\;\delta_{𝔥}{Ȿ}_{𝔥}+\theta {𝔎}_{𝔥}-\pi_{𝔥}\\ +\alpha_{𝔪}\left(\chi_{{Ȿ}_{𝔥}}+\chi_{A_{𝔥}}\right)\bar{{Ȿ}_{𝔥}}\bar{{𝔗}_{𝔪}}\;+\delta_{𝔥}\bar{{Ȿ}_{𝔥}}\theta \bar{{𝔎}_{𝔥}}|\\ +|\alpha_{𝔪}\chi_{{Ȿ}_{𝔥}}{Ȿ}_{𝔥}{𝔗}_{𝔪}\;-\;\left(\tau +\gamma +\delta_{𝔥}\right){𝔗}_{{Ȿ}_{𝔥}}-\alpha_{𝔪}\chi_{{Ȿ}_{𝔥}}\bar{{Ȿ}_{𝔥}}\bar{{𝔗}_{𝔪}}\\ \;+\left(\tau +\gamma +\delta_{𝔥}\right)\bar{{𝔗}_{{Ȿ}_{𝔥}}}|\\ +|\alpha_{𝔪}\chi_{A_{𝔥}}{Ȿ}_{𝔥}{𝔗}_{𝔪}\;-\;\left(\varrho +\gamma +\delta_{𝔥}\right){\;𝔗}_{A_{𝔥}}-\;\alpha_{𝔪}\chi_{A_{𝔥}}\bar{{Ȿ}_{𝔥}}\bar{{𝔗}_{𝔪}}\;\\ +\left(\varrho +\gamma +\delta_{𝔥}\right)\bar{{\;𝔗}_{A_{𝔥}}}|\;\\ +|\gamma \left({𝔗}_{{Ȿ}_{𝔥}}+{\;𝔗}_{A_{𝔥}}\right)-\theta {𝔎}_{𝔥}-\delta_{𝔥}{𝔎}_{𝔥}+\tau \;{𝔗}_{{Ȿ}_{𝔥}}+\varrho {\;𝔗}_{A_{𝔥}}\\ -\gamma \left(\bar{{𝔗}_{{Ȿ}_{𝔥}}}+\bar{{\;𝔗}_{A_{𝔥}}}\right)+\theta \bar{{𝔎}_{𝔥}}+\delta_{𝔥}\bar{{𝔎}_{𝔥}}-\tau \bar{{𝔗}_{{Ȿ}_{𝔥}}}-\varrho \bar{{\;𝔗}_{A_{𝔥}}}|\\ +|\pi_{𝔪}\;-\alpha_{𝔪}\chi_{𝔪}\left({𝔗}_{{Ȿ}_{𝔥}}+{\;𝔗}_{A_{𝔥}}\right){Ȿ}_{𝔪}-\delta_{𝔪}{Ȿ}_{𝔪}-\pi_{𝔪}+\alpha_{𝔪}\chi_{𝔪}\\ \left(\bar{{𝔗}_{{Ȿ}_{𝔥}}}+\bar{{\;𝔗}_{A_{𝔥}}}\right)\bar{{Ȿ}_{𝔪}}+\delta_{𝔪}\bar{{Ȿ}_{𝔪}}|+|\alpha_{𝔪}\chi_{𝔪}\\ \left({𝔗}_{{Ȿ}_{𝔥}}+{\;𝔗}_{A_{𝔥}}\right){Ȿ}_{𝔪}-\delta_{𝔪}{𝔗}_{𝔪}-\alpha_{𝔪}\chi_{𝔪}\left(\bar{{𝔗}_{{Ȿ}_{𝔥}}}+\bar{{\;𝔗}_{A_{𝔥}}}\right)\;\bar{{Ȿ}_{𝔪}}\;+\delta_{𝔪}\bar{{𝔗}_{𝔪}}|.\;\\ \leq \left[2\alpha_{𝔪}\left(\chi_{{Ȿ}_{𝔥}}+\chi_{A_{𝔥}}\right)P+\delta_{𝔥}\right]|{Ȿ}_{𝔥}\;-\bar{{Ȿ}_{𝔥}}|+\left(2\tau +2\gamma +\delta_{𝔥}\right)\\ |{𝔗}_{{Ȿ}_{𝔥}}-\bar{{𝔗}_{{Ȿ}_{𝔥}}}|+\left(2\varrho +2\gamma +\delta_{𝔥}\right)\;\\ |{\;𝔗}_{A_{𝔥}}-\;\bar{{\;𝔗}_{A_{𝔥}}}|+\left(2\theta {+\delta }_{𝔥}\right)|{𝔎}_{𝔥}-\;\bar{{𝔎}_{𝔥}}|+\left[2\alpha_{𝔪}\left(Q+R\right)\chi_{𝔪}+\delta_{𝔥}\right]\\ |{Ȿ}_{𝔪}\;-\bar{{Ȿ}_{𝔪}}|+\delta_{𝔪}|{𝔗}_{\text{m}}-\bar{{𝔗}_{\text{m}}}|K\leq ||x-\bar{x}\;||. \end{array}$$

Where $K=\max\left[\begin{array}{c} 2\alpha_{𝔪}\left(\chi_{{Ȿ}_{𝔥}}+\chi_{A_{𝔥}}\right)P+\delta_{𝔥},\\ 2\left(\tau +\gamma \right)+\delta_{𝔥},2\left(\varrho +\gamma \right)+\delta_{𝔥},2\theta {+\delta }_{𝔥},\\ 2\alpha_{𝔪}\left(Q+R\right)\chi_{𝔪}+\delta_{𝔥},\delta_{𝔪} \end{array}\right]$

Basically, since 𝕄(x) satisfies the Lipschitz requirement. Model (Equation 6) has a singular solution based on Lemma 1.

### 4.2 Positivity solution

Since system (Equation 6) deals with mosquitoes and populace, all components of system are positive. Following is our discussion:

**Theorem 4.2**.

Let (Ȿ_𝔥_, 𝔗_Ȿ_𝔥__, $\;{𝔗}_{A_{𝔥}}$, 𝔎_𝔥_, Ȿ_𝔪_, 𝔗_𝔪_) > 0 be represent the system (Equation 6) solution for the primary points ${\;Ȿ}_{𝔥}\left(0\right),\;{𝔗}_{{Ȿ}_{𝔥}}\left(0\right),\;{\;𝔗}_{A_{𝔥}}\left(0\right),{𝔎}_{𝔥}\left(0\right),{Ȿ}_{𝔪}\left(0\right),\;{𝔗}_{𝔪}\left(0\right)$ and represents an immutable set

$\Phi =\left\{{Ȿ}_{𝔥}\;,\;{𝔗}_{{Ȿ}_{𝔥}},\;{𝔗}_{A_{𝔥}},\;{𝔎}_{𝔥},{Ȿ}_{𝔪},\;{𝔗}_{𝔪}\in \;{\mathbb{R}}_{+}^{6}:\;N_{𝔥}=\frac{\pi_{𝔥}}{\;\delta_{𝔥}},\;N_{𝔪}=\frac{\pi_{𝔪}}{\delta_{𝔪}}\right\}$, then, all elements of the closed set

Φ is traveling in ${\mathbb{R}}_{+}^{6}$ space is positive invariant.

**Proof**

The given equation is used to construct the Lyapunov function:


$$\begin{array}{l} 𝕃\left(ȶ\right)=\left({𝕃}_{1}\left(ȶ\right),\;{𝕃}_{2}\left(ȶ\right)\right)=\left({Ȿ}_{𝔥}+\;{𝔗}_{{Ȿ}_{𝔥}}+{\;𝔗}_{A_{𝔥}}+{𝔎}_{𝔥},\;{Ȿ}_{𝔪}+{𝔗}_{𝔪}\right)\; \end{array}$$

The function 𝕃(ȶ) satisfies


(7) $$\begin{array}{l} \dot{𝕃\left(ȶ\right)}=(\dot{{𝕃}_{1}\left(ȶ\right),}\;\dot{{𝕃}_{2}\left(ȶ\right),})=\left(\dot{{Ȿ}_{𝔥}}+\dot{{\;𝔗}_{{Ȿ}_{𝔥}}}+\dot{{\;𝔗}_{A_{𝔥}}}+\dot{{𝔎}_{𝔥}},\dot{{Ȿ}_{𝔪}}+\dot{{\;𝔗}_{𝔪}}\;\right)\\ =\left(\pi_{𝔥}-\;\delta_{𝔥}{Ȿ}_{𝔥}-{𝔗}_{{Ȿ}_{𝔥}}\delta_{𝔥}-{\;𝔗}_{A_{𝔥}}\delta_{𝔥}{-\;\delta }_{𝔥}{𝔎}_{𝔥},\pi_{𝔪}-\delta_{𝔪}{Ȿ}_{𝔪}-\delta_{𝔪}{𝔗}_{𝔪}\right)\;\\ =\left(\pi_{𝔥}-\;\delta_{𝔥}{𝕃}_{1},\pi_{𝔪}-\delta_{𝔪}{𝕃}_{2}\right) \end{array}$$

Therefore, it is simple to demonstrate Equation 7 as regards:


(8) $$\begin{array}{l} \{\begin{array}{c} \dot{{𝕃}_{1}\left(ȶ\right)}=\pi_{𝔥}-\;\delta_{𝔥}{𝕃}_{1}\leq 0,\;for\;{𝕃}_{1}\geq \frac{\pi_{𝔥}}{\;\delta_{𝔥}}\\ \dot{{𝕃}_{2}\left(ȶ\right)}=\pi_{𝔪}-\delta_{𝔪}{𝕃}_{2}\leq 0,\;for\;{𝕃}_{2}\geq \frac{\pi_{𝔪}}{\delta_{𝔪}} \end{array} \end{array}$$

Inferring $\dot{𝕃\left(ȶ\right)}\leq 0$ from the above equations, which indicates that *f* is positively stable collection. On the other hand, by solving system (Equation 6)


$$\begin{array}{l} 0\leq \;\left({𝕃}_{1}\left(ȶ\right),{𝕃}_{2}\left(ȶ\right)\right)<\left(\frac{\pi_{𝔥}}{\;\delta_{𝔥}}+{𝕃}_{1}\left(0\right)e^{-\;\delta_{𝔥}t},\frac{\pi_{𝔪}}{\delta_{𝔪}}+{𝕃}_{2}\left(0\right)e^{-\;\delta_{𝔪}t}\right) \end{array}$$

Where 𝕃_1_(0) and 𝕃_2_(0) are the primary states of 𝕃_1_(ȶ) and 𝕃_2_(ȶ) respectively. Therefore, *t* → ∞, $0\leq \;\left({𝕃}_{1}\left(ȶ\right),{𝕃}_{2}\left(ȶ\right)\right)\leq \;\left(\frac{\pi_{𝔥}}{\;\delta_{𝔥}},\frac{\pi_{𝔪}}{\delta_{𝔪}}\right)$ and we can conclusion that Φ is a desirable set.

This establishes the theorem.

### 4.3 Basic reproduction value $R_{0}$

Let $C_{f}=\left({Ȿ}_{𝔥}^{*}\;,\;{𝔗}_{{Ȿ}_{𝔥}}^{*},\;{𝔗}_{A_{𝔥}}^{*},{𝔎}_{𝔥}^{*},{Ȿ}_{𝔪}^{*},\;{𝔗}_{𝔪}^{*}\right)$ be the contagious free equilibrium of Equation 6. We have $C_{f}=\;\left(\frac{\pi_{𝔥}}{\;\delta_{𝔥}},\;0,\;0,\;\frac{\pi_{𝔪}}{\delta_{𝔪}},\;0\right)$. The algorithm of the next iteration matrix is utilized to estimate $R_{0}$. Obviously, the infected compartments are 𝔗_Ȿ_𝔥__, ${\;𝔗}_{A_{𝔥}}$ and 𝔗_𝔪_ as a consequence of Equation 6. There are


(9) $$\begin{array}{l} \begin{array}{c} AℬC\\ 0 \end{array}{𝔇}_{ȶ}^{\eta }{𝔗}_{{Ȿ}_{𝔥}}=\alpha_{𝔪}\chi_{{Ȿ}_{𝔥}}{Ȿ}_{𝔥}{𝔗}_{𝔪}\;-\;\left(\tau +\gamma +\delta_{𝔥}\right){𝔗}_{{Ȿ}_{𝔥}}\;\\ \begin{array}{c} AℬC\\ 0 \end{array}{𝔇}_{ȶ}^{\eta }{\;𝔗}_{A_{𝔥}}=\;\alpha_{𝔪}\chi_{A_{𝔥}}{Ȿ}_{𝔥}{𝔗}_{𝔪}\;-\;\left(\varrho +\gamma +\delta_{𝔥}\right){\;𝔗}_{A_{𝔥}}\;\\ \begin{array}{c} AℬC\\ 0 \end{array}{𝔇}_{ȶ}^{\eta }{𝔗}_{𝔪}=\;\alpha_{𝔪}\chi_{𝔪}\left({𝔗}_{{Ȿ}_{𝔥}}+{\;𝔗}_{A_{𝔥}}\right){Ȿ}_{𝔪}-\delta_{𝔪}{𝔗}_{𝔪}\; \end{array}$$

Then we derive


$$\begin{array}{l} F=\left(\begin{array}{ccc} 0 & 0 & \alpha_{𝔪}\chi_{{Ȿ}_{𝔥}}{Ȿ}_{𝔥}\\ 0 & 0 & \alpha_{𝔪}\chi_{A_{𝔥}}{Ȿ}_{𝔥}\\ \alpha_{𝔪}\chi_{𝔪}{Ȿ}_{𝔪} & \alpha_{𝔪}\chi_{𝔪}{Ȿ}_{𝔪} & 0 \end{array}\right),\\ V=\left(\begin{array}{ccc} \tau +\gamma +\delta_{𝔥} & 0 & 0\\ 0 & \varrho +\gamma +\delta_{𝔥} & 0\\ 0 & 0 & \delta_{𝔪} \end{array}\right) \end{array}$$

The basic reproduction value is given by


$$\begin{array}{l} R_{0}=\;\rho \left({\mathrm{FV}}^{-1}\right)=\;\frac{\pi_{𝔥}\pi_{𝔪}\chi_{𝔪}{\alpha_{𝔪}}^{2}\left(\chi_{{Ȿ}_{𝔥}}+\chi_{A_{𝔥}}\right)}{\delta_{𝔥}{\left(\delta_{𝔪}\right)}^{2}\left(\tau +\gamma +\delta_{𝔥}\right)\left(\varrho +\gamma +\delta_{𝔥}\right)}\; \end{array}$$

Where $\rho \left({\mathrm{FV}}^{-1}\right)$ denote the spectral radius. Surmise that $C_{p}=\left({Ȿ}_{𝔥}^{**},\;{𝔗}_{{Ȿ}_{𝔥}}^{**},\;{𝔗}_{A_{𝔥}}^{**},\;{𝔎}_{𝔥}^{**},\;{Ȿ}_{𝔪}^{**},\;{𝔗}_{𝔪}^{**}\right)$ represents the endemic equilibrium for Equation 6. So that


$$\begin{array}{l} {Ȿ}_{𝔥}^{**}=\frac{\pi_{𝔥}\left(\theta +\delta_{𝔥}\right)u_{1}u_{2}}{\left(\alpha_{𝔪}\left(\chi_{{Ȿ}_{𝔥}}+\chi_{A_{𝔥}}\right){𝔗}_{𝔪}+\delta_{𝔥}\right)u_{1}u_{2}\left(\theta +\delta_{𝔥}\right)-\theta \alpha_{𝔪}{{𝔗}_{𝔪}u}_{4}}\\ {𝔗}_{{Ȿ}_{𝔥}}^{**}=\;\frac{\alpha_{𝔪}\chi_{{Ȿ}_{𝔥}}{Ȿ}_{𝔥}{𝔗}_{𝔪}}{u_{1}}\;\\ {𝔗}_{A_{𝔥}}^{**}=\frac{\alpha_{𝔪}\chi_{A_{𝔥}}{Ȿ}_{𝔥}{𝔗}_{𝔪}}{u_{2}}\;\\ {𝔎}_{𝔥}^{**}=\frac{\alpha_{𝔪}{Ȿ}_{𝔥}{𝔗}_{𝔪}u_{4}}{{\left(\theta +\delta_{𝔥}\right)u}_{1}u_{2}}\;\\ {Ȿ}_{𝔪}^{**}=\frac{u_{1}u_{2}\pi_{𝔪}}{{\alpha_{𝔪}}^{2}\chi_{𝔪}{Ȿ}_{𝔥}{𝔗}_{𝔪}u_{3}+\delta_{𝔪}u_{1}u_{2}}\;\\ {𝔗}_{𝔪}^{**}=\frac{{\left(\theta +\delta_{𝔥}\right)u}_{3}\alpha_{𝔪}\pi_{𝔥}{Ȿ}_{𝔪}\;-\delta_{𝔥}u_{1}u_{2}\delta_{𝔪}}{\left(\chi_{{Ȿ}_{𝔥}}+\chi_{A_{𝔥}}\right)u_{5}-\;\theta u_{4}}\; \end{array}$$

Where $u_{1}=\;\tau +\gamma +\delta_{𝔥},\;u_{2}=\;\varrho +\gamma +\delta_{𝔥},\;u_{3}=\;u_{1}\chi_{A_{𝔥}}+\;u_{2}\chi_{{Ȿ}_{𝔥}},\;u_{4}=\;u_{1}\chi_{A_{𝔥}}\left(\tau +\gamma \right)+\;u_{2}\chi_{{Ȿ}_{𝔥}}\left(\varrho +\gamma \right),$ and *u*_5_ = *u*_1_*u*_2_(θ+δ_𝔥_)δ_𝔪_.

### 4.4 Local stability

In this part, we are covering the analysis of firmness conditions of contagious free equilibrium $C_{f}$ and contagious persistence equilibrium $C_{p}$ points. A steady state analysis of this equilibrium results in the following Theorem 4.3 and Theorem 4.4.

The obtained Jacobian matrix is:


(10) $$\begin{array}{l} ℑ=\left(\begin{array}{cccccc} {-\alpha }_{𝔪}\left(\chi_{{Ȿ}_{𝔥}}+\chi_{A_{𝔥}}\right){𝔗}_{𝔪}\;-\delta_{𝔥} & 0 & 0 & \theta & 0 & {-\alpha }_{𝔪}\left(\chi_{{Ȿ}_{𝔥}}+\chi_{A_{𝔥}}\right){Ȿ}_{𝔥}\\ \alpha_{𝔪}\chi_{{Ȿ}_{𝔥}}{𝔗}_{𝔪} & -\left(\tau +\gamma +\delta_{𝔥}\right) & 0 & 0 & 0 & \alpha_{𝔪}\chi_{{Ȿ}_{𝔥}}{Ȿ}_{𝔥}\\ \alpha_{𝔪}\chi_{A_{𝔥}}{𝔗}_{𝔪} & 0 & -\left(\varrho +\gamma +\delta_{𝔥}\right) & 0 & 0 & \alpha_{𝔪}\chi_{A_{𝔥}}{Ȿ}_{𝔥}\\ 0 & \tau +\gamma & \varrho +\gamma & -\left(\theta +\delta_{𝔥}\right) & 0 & 0\\ 0 & {-\alpha }_{𝔪}\chi_{𝔪}{Ȿ}_{𝔪} & -\alpha_{𝔪}\chi_{𝔪}{Ȿ}_{𝔪} & 0 & -\left(\alpha_{𝔪}\chi_{𝔪}\left({𝔗}_{{Ȿ}_{𝔥}}+{𝔗}_{A_{𝔥}}\right)+\delta_{𝔪}\right) & 0\\ 0 & \alpha_{𝔪}\chi_{𝔪}{Ȿ}_{𝔪} & \alpha_{𝔪}\chi_{𝔪}{Ȿ}_{𝔪} & 0 & \alpha_{𝔪}\chi_{𝔪}\left({𝔗}_{{Ȿ}_{𝔥}}+{𝔗}_{A_{𝔥}}\right) & -\delta_{𝔪} \end{array}\right) \end{array}$$

**Theorem 4.3**.

If $R_{0}<1,$ the non - contagious equilibrium ${C\;}_{f}$ is locally stable.

**Proof**

The structure (Equation 6) in the Jacobian matrix of $C_{f}$ follows


(11) $$\begin{array}{l} ℑ\left(C_{f}\right)=\left(\begin{array}{cccccc} \;-\delta_{𝔥} & 0 & 0 & \theta & 0 & {-\alpha }_{𝔪}\left(\chi_{{Ȿ}_{𝔥}}+\chi_{A_{𝔥}}\right)\frac{\pi_{𝔥}}{\delta_{𝔥}}\\ 0 & -\left(\tau +\gamma +\delta_{𝔥}\right) & 0 & 0 & 0 & \alpha_{𝔪}\chi_{{Ȿ}_{𝔥}}\frac{\pi_{𝔥}}{\delta_{𝔥}}\\ 0 & 0 & -\left(\varrho +\gamma +\delta_{𝔥}\right) & 0 & 0 & \alpha_{𝔪}\chi_{A_{𝔥}}\frac{\pi_{𝔥}}{\delta_{𝔥}}\\ 0 & \tau +\gamma & \varrho +\gamma & -\left(\theta +\delta_{𝔥}\right) & 0 & 0\\ 0 & {-\alpha }_{𝔪}\chi_{𝔪}\frac{\pi_{𝔪}}{\delta_{𝔪}} & {-\alpha }_{𝔪}\chi_{𝔪}\frac{\pi_{𝔪}}{\delta_{𝔪}} & 0 & \delta_{𝔪} & 0\\ 0 & \alpha_{𝔪}\chi_{𝔪}\frac{\pi_{𝔪}}{\delta_{𝔪}} & \alpha_{𝔪}\chi_{𝔪}\frac{\pi_{𝔪}}{\delta_{𝔪}} & 0 & 0 & -\delta_{𝔪} \end{array}\right) \end{array}$$

To determine the eigenvalue from the above-described matrix $\det\left(ℑ\left(C_{f}\right)-\lambda 𝔗\right)=\;0$

We obtain the Eigen values λ_1_ = −δ_𝔥_, λ_2_ = −(τ+γ+δ_𝔥_), λ_3_ = δ_𝔪_, λ_4_ = θ+δ_𝔥_ and the characteristic relation is


$$\begin{array}{l} \;\lambda^{2}+\left(\varrho +\gamma +\delta_{𝔥}+\delta_{𝔪}\right)\;\lambda +\delta_{𝔪}\left(\tau +\gamma +\delta_{𝔥}\right)\left(\varrho +\gamma +\delta_{𝔥}\right)\\ \left[1-R_{0}\right]=0\; \end{array}$$

When $R_{0}<1,$ it is obvious that λ_5_ < 1 and λ_6_ < 1, all the Eigen values satisfy the condition $\left|arg(\lambda_{i})\right|>\frac{\eta \pi }{2},\;i=1,2,\ldots ,6.$ the without contagious equilibrium $C_{f}$ is locally asymptotically stable.

**Theorem 4.4**.

If $R_{0}>1$, the equilibrium point $C_{p}$ is locally stable, then system (Equation 6) has ubiquitous contagion.

Proof

Jacobian matrix evaluated in static equilibrium:


(12) $$\begin{array}{l} \det\left(ℑ\left(C_{p}\right)-\lambda 𝔗\right)=0\; \end{array}$$

We obtain the Eigen values are λ_1_ = (θ+δ_𝔥_), $\lambda_{2}=\left(\alpha_{𝔪}\chi_{𝔪}\left({𝔗}_{{Ȿ}_{𝔥}}^{**}+{𝔗}_{A_{𝔥}}^{**}\right)+\;\delta_{𝔪}\right)$,

$\lambda_{3}=\alpha_{𝔪}\left(\chi_{{Ȿ}_{𝔥}}+\chi_{A_{𝔥}}\right){𝔗}_{𝔪}^{**}+\delta_{𝔥}$ and the characteristic relation


(13) $$\begin{array}{l} \lambda^{3}+a_{1}\lambda^{2}+a_{2}\lambda +a_{3}=0\; \end{array}$$

Where


$$\begin{array}{l} a_{1}=\left(\tau +\varrho +2\left(\gamma +\delta_{𝔥}\right)\right)\;\\ a_{2}=\left(\varrho +\gamma +\delta_{𝔥}+\delta_{𝔪}\right)\left[\left(\tau +\gamma +\delta_{𝔥}\right)-{\alpha_{𝔪}}^{2}\left(\chi_{{Ȿ}_{𝔥}}+\chi_{A_{𝔥}}\right)\chi_{𝔪}{Ȿ}_{𝔥}^{**}{Ȿ}_{𝔪}^{**}\right]\;\\ a_{3}=\left(\tau +\gamma +\delta_{𝔥}\right)\left[\left(\varrho +\gamma +\delta_{𝔥}\right)\delta_{𝔪}-{\alpha_{𝔪}}^{2}\left(\chi_{{Ȿ}_{𝔥}}+\chi_{A_{𝔥}}\right)\chi_{𝔪}{Ȿ}_{𝔥}^{**}{Ȿ}_{𝔪}^{**}\right]\; \end{array}$$

By using Routh-Hurwitz Criteria (22, 23), if the following provisions are handling


$$\begin{array}{l} a_{1}>0,a_{2}>0,a_{3}>0\text{ and }a_{1}a_{2}-a_{3}>\;0. \end{array}$$

Then $C_{p}$ is approximately stable locally. The evidence is conclusive.

### 4.5 Global stability

**Theorem 4.5**.

If $R_{0}<1,$ the point of without contagious equilibrium $C_{f\;}$ is global stability on Φ.

**Proof**

Create a Lyapunov function 𝕍_1_(ȶ),


(14) $$\begin{array}{l} {\;𝕍}_{1}\left(ȶ\right)=\left({Ȿ}_{𝔥}-{Ȿ}_{𝔥}^{*}\ln{Ȿ}_{𝔥}\right)+{𝔗}_{{Ȿ}_{𝔥}}+{\;𝔗}_{A_{𝔥}}+{𝔎}_{𝔥}+\left({Ȿ}_{𝔪}-{Ȿ}_{𝔪}^{*}\ln{Ȿ}_{𝔪}\right)\;+\;{𝔗}_{𝔪} \end{array}$$

Calculating the fractional order derivatives of 𝕍_1_(ȶ) in the solution direction of Equation 6, from Lemma 2, we obtain


$$\begin{array}{l} \begin{array}{c} AℬC\\ 0 \end{array}{𝔇}_{ȶ}^{\eta }{\;𝕍}_{1}\left(ȶ\right)\leq \left(1-\frac{{Ȿ}_{𝔥}^{*}}{{Ȿ}_{𝔥}}\right)\begin{array}{c} AℬC\\ 0 \end{array}{𝔇}_{ȶ}^{\eta }{Ȿ}_{𝔥}\\ +\begin{array}{c} AℬC\\ 0 \end{array}{𝔇}_{ȶ}^{\eta }{𝔗}_{{Ȿ}_{𝔥}}+\begin{array}{c} AℬC\\ 0 \end{array}{𝔇}_{ȶ}^{\eta }{\;𝔗}_{A_{𝔥}}+\begin{array}{c} AℬC\\ 0 \end{array}{𝔇}_{ȶ}^{\eta }{𝔎}_{𝔥}\\ +\left(1-\frac{{Ȿ}_{𝔪}^{*}}{{Ȿ}_{𝔪}}\right)\begin{array}{c} AℬC\\ 0 \end{array}{𝔇}_{ȶ}^{\eta }{Ȿ}_{𝔪}+\begin{array}{c} AℬC\\ 0 \end{array}{𝔇}_{ȶ}^{\eta }{𝔗}_{𝔪}\;\\ \leq \left(1-\frac{{Ȿ}_{𝔥}^{*}}{{Ȿ}_{𝔥}}\right)\left(\pi_{𝔥}\;-\alpha_{𝔪}\left(\chi_{{Ȿ}_{𝔥}}+\chi_{A_{𝔥}}\right){Ȿ}_{𝔥}{𝔗}_{𝔪}-\delta_{𝔥}{Ȿ}_{𝔥}+\theta {𝔎}_{𝔥}\right)\\ +\left(\alpha_{𝔪}\chi_{{Ȿ}_{𝔥}}{Ȿ}_{𝔥}{𝔗}_{𝔪}\;-\;\left(\tau +\gamma +\delta_{𝔥}\right){𝔗}_{{Ȿ}_{𝔥}}\right)\;\\ +\left(\alpha_{𝔪}\chi_{A_{𝔥}}{Ȿ}_{𝔥}{𝔗}_{𝔪}\;-\;\left(\varrho +\gamma +\delta_{𝔥}\right){\;𝔗}_{A_{𝔥}}\right)\;+(\gamma \left({𝔗}_{{Ȿ}_{𝔥}}+{\;𝔗}_{A_{𝔥}}\right)\\ -\theta {𝔎}_{𝔥}-\delta_{𝔥}{𝔎}_{𝔥}+\tau \;{𝔗}_{{Ȿ}_{𝔥}}+\varrho {\;𝔗}_{A_{𝔥}})\;\\ +\left(1-\frac{{Ȿ}_{𝔪}^{*}}{{Ȿ}_{𝔪}}\right)\left(\pi_{𝔪}\;-\alpha_{𝔪}\chi_{𝔪}\left({𝔗}_{{Ȿ}_{𝔥}}+{\;𝔗}_{A_{𝔥}}\right){Ȿ}_{𝔪}-\delta_{𝔪}{Ȿ}_{𝔪}\right)\\ +\left(\alpha_{𝔪}\chi_{𝔪}\left({𝔗}_{{Ȿ}_{𝔥}}+{\;𝔗}_{A_{𝔥}}\right){Ȿ}_{𝔪}-\delta_{𝔪}{𝔗}_{𝔪}\right)\;\\ \leq \pi_{𝔥}\left(1-\frac{{Ȿ}_{𝔥}^{*}}{{Ȿ}_{𝔥}}\right)+\;\alpha_{𝔪}\chi_{{Ȿ}_{𝔥}}{Ȿ}_{𝔥}^{*}{𝔗}_{𝔪}+\alpha_{𝔪}\chi_{A_{𝔥}}{Ȿ}_{𝔥}^{*}{𝔗}_{𝔪}\\ -\delta_{𝔥}{Ȿ}_{𝔥}+\delta_{𝔥}{Ȿ}_{𝔥}^{*}+\theta {𝔎}_{𝔥}\left(1-\frac{{Ȿ}_{𝔥}^{*}}{{Ȿ}_{𝔥}}\right)-\delta_{𝔥}{𝔗}_{{Ȿ}_{𝔥}}\\ -\delta_{𝔥}{𝔗}_{A_{𝔥}}-\theta {𝔎}_{𝔥}-\delta_{𝔥}{𝔎}_{𝔥}+\pi_{𝔪}\left(1-\frac{{Ȿ}_{𝔪}^{*}}{{Ȿ}_{𝔪}}\right)+\alpha_{𝔪}\chi_{𝔪}\\ \left({𝔗}_{{Ȿ}_{𝔥}}+{\;𝔗}_{A_{𝔥}}\right){Ȿ}_{𝔪}^{*}-\delta_{𝔪}\left(1-\frac{{Ȿ}_{𝔪}^{*}}{{Ȿ}_{𝔪}}\right)-\delta_{𝔪}{𝔗}_{𝔪}\; \end{array}$$

Substituting the reaction of without contagious free $C_{f}=\;\left(\frac{\pi_{𝔥}}{\;\delta_{𝔥}},\;0,\;0,\;\frac{\pi_{𝔪}}{\delta_{𝔪}},\;0\right)$, we obtain:


(15) $$\begin{array}{l} \begin{array}{c} AℬC\\ 0 \end{array}{𝔇}_{ȶ}^{\eta }{\;𝕍}_{1}\left(ȶ\right)\leq \left(\pi_{𝔥}+\delta_{𝔥}\right)\left(2-\frac{{Ȿ}_{𝔥}^{*}}{{Ȿ}_{𝔥}}-\frac{{Ȿ}_{𝔥}}{{Ȿ}_{𝔥}^{*}}\right)+\left(\pi_{𝔪}-\delta_{𝔪}\right)\left(2-\frac{{Ȿ}_{𝔪}^{*}}{{Ȿ}_{𝔪}}-\frac{{Ȿ}_{𝔪}}{{Ȿ}_{𝔪}^{*}}\right) \end{array}$$

It is clear that each term in Equation 15 must be negative. We have $\begin{array}{c} AℬC\\ 0 \end{array}{𝔇}_{ȶ}^{\eta }{\;𝕍}_{1}\left(ȶ\right)\leq 0$ due to LaSalle's invariance principle (24), the function $\begin{array}{c} AℬC\\ 0 \end{array}{𝔇}_{ȶ}^{\eta }{\;𝕍}_{1}\left(ȶ\right)$ is required to be negative finite.

The maximally invariant sets ${Ȿ}_{𝔥}=\;{Ȿ}_{𝔥}^{*}$, ${Ȿ}_{𝔪}={Ȿ}_{𝔪}^{*}$ which is singleton $C_{f}=\left({Ȿ}_{𝔥}^{*}\;,\;{𝔗}_{{Ȿ}_{𝔥}}^{*},\;{𝔗}_{A_{𝔥}}^{*},{𝔎}_{𝔥}^{*},{Ȿ}_{𝔪}^{*},\;{𝔗}_{𝔪}^{*}\right)$ contains the limit set for each solution. This demonstrates $C_{f\;}$ is globally asymptotically stable on Φ.

**Theorem 4.6**.

When $R_{0}>1,$ the positive contagious equalization level of system (Equation 6) arises and is globally stable on Φ.

**Proof**

Let's create a lyapunov function of the following form


(16) $$\begin{array}{l} {\;𝕍}_{2}\left(ȶ\right)=\left({Ȿ}_{𝔥}-{Ȿ}_{𝔥}^{**}\ln{Ȿ}_{𝔥}\right)+\left({𝔗}_{{Ȿ}_{𝔥}}-{𝔗}_{{Ȿ}_{𝔥}}^{**}\ln{𝔗}_{{Ȿ}_{𝔥}}\right)\\ +\left({\;𝔗}_{A_{𝔥}}-{𝔗}_{A_{𝔥}}^{**}\ln{\;𝔗}_{A_{𝔥}}\right)\\ +\left({𝔎}_{𝔥}-{𝔎}_{𝔥}^{**}\ln{𝔎}_{𝔥}\right)+\left({Ȿ}_{𝔪}-{Ȿ}_{𝔪}^{**}\ln{Ȿ}_{𝔪}\right)+\left({𝔗}_{𝔪}-{𝔗}_{𝔪}^{**}\ln{𝔗}_{𝔪}\right)\;\\ \begin{array}{c} AℬC\\ 0 \end{array}{𝔇}_{ȶ}^{\eta }{\;𝕍}_{2}\left(ȶ\right)\leq \left(1-\frac{{Ȿ}_{𝔥}^{**}}{{Ȿ}_{𝔥}}\right)\begin{array}{c} AℬC\\ 0 \end{array}{𝔇}_{ȶ}^{\eta }{Ȿ}_{𝔥}\\ +\left(1-\frac{{𝔗}_{{Ȿ}_{𝔥}}^{**}}{{𝔗}_{{Ȿ}_{𝔥}}}\right)\begin{array}{c} AℬC\\ 0 \end{array}{𝔇}_{ȶ}^{\eta }{𝔗}_{{Ȿ}_{𝔥}}+\left(1-\frac{{𝔗}_{A_{𝔥}}^{**}}{{\;𝔗}_{A_{𝔥}}}\right)\begin{array}{c} AℬC\\ 0 \end{array}{𝔇}_{ȶ}^{\eta }{\;𝔗}_{A_{𝔥}}+\;\\ \left(1-\frac{{𝔎}_{𝔥}^{**}}{{𝔎}_{𝔥}}\right)\begin{array}{c} AℬC\\ 0 \end{array}{𝔇}_{ȶ}^{\eta }{𝔎}_{𝔥}\\ +\left(1-\frac{{Ȿ}_{𝔪}^{**}}{{Ȿ}_{𝔪}}\right)\begin{array}{c} AℬC\\ 0 \end{array}{𝔇}_{ȶ}^{\eta }{Ȿ}_{𝔪}+\left(1-\frac{{𝔗}_{𝔪}^{**}}{{𝔗}_{𝔪}}\right)\begin{array}{c} AℬC\\ 0 \end{array}{𝔇}_{ȶ}^{\eta }{𝔗}_{𝔪}\;\\ \leq \left(1-\frac{{Ȿ}_{𝔥}^{**}}{{Ȿ}_{𝔥}}\right)\left(\pi_{𝔥}\;-\alpha_{𝔪}\left(\chi_{{Ȿ}_{𝔥}}+\chi_{A_{𝔥}}\right){Ȿ}_{𝔥}{𝔗}_{𝔪}\;-\delta_{𝔥}{Ȿ}_{𝔥}+\theta {𝔎}_{𝔥}\right)\\ +\left(1-\frac{{𝔗}_{{Ȿ}_{𝔥}}^{**}}{{𝔗}_{{Ȿ}_{𝔥}}}\right)\left(\alpha_{𝔪}\chi_{{Ȿ}_{𝔥}}{Ȿ}_{𝔥}{𝔗}_{𝔪}\;-\left(\tau +\gamma +\delta_{𝔥}\right){𝔗}_{{Ȿ}_{𝔥}}\right)\;\\ +\left(1-\frac{{𝔗}_{A_{𝔥}}^{**}}{{\;𝔗}_{A_{𝔥}}}\right)\left(\alpha_{𝔪}\chi_{A_{𝔥}}{Ȿ}_{𝔥}{𝔗}_{𝔪}-\left(\varrho +\gamma +\delta_{𝔥}\right){\;𝔗}_{A_{𝔥}}\right)+\left(1-\frac{{𝔎}_{𝔥}^{**}}{{𝔎}_{𝔥}}\right)\\ \left(\gamma \left({𝔗}_{{Ȿ}_{𝔥}}+{𝔗}_{A_{𝔥}}\right)-\theta {𝔎}_{𝔥}-\delta_{𝔥}{𝔎}_{𝔥}+\tau {𝔗}_{{Ȿ}_{𝔥}}+\;\varrho {\;𝔗}_{A_{𝔥}}\right)\;\\ +\left(1-\frac{{Ȿ}_{𝔪}^{**}}{{Ȿ}_{𝔪}}\right)\left(\pi_{𝔪}\;-\alpha_{𝔪}\chi_{𝔪}\left({𝔗}_{{Ȿ}_{𝔥}}+{\;𝔗}_{A_{𝔥}}\right){Ȿ}_{𝔪}-\delta_{𝔪}{Ȿ}_{𝔪}\right)+\left(1-\frac{{𝔗}_{𝔪}^{**}}{{𝔗}_{𝔪}}\right)\\ \left(\alpha_{𝔪}\chi_{𝔪}\left({𝔗}_{{Ȿ}_{𝔥}}+{\;𝔗}_{A_{𝔥}}\right){Ȿ}_{𝔪}-\delta_{𝔪}{𝔗}_{𝔪}\right)\;\\ \leq \pi_{𝔥}\left(1-\frac{{Ȿ}_{𝔥}^{**}}{{Ȿ}_{𝔥}}\right)+\alpha_{𝔪}\left(\chi_{{Ȿ}_{𝔥}}+\chi_{A_{𝔥}}\right){Ȿ}_{𝔥}^{**}{𝔗}_{𝔪}\;-\delta_{𝔥}{Ȿ}_{𝔥}\left(1-\frac{{Ȿ}_{𝔥}^{**}}{{Ȿ}_{𝔥}}\right)\\ +\theta {𝔎}_{𝔥}\frac{{Ȿ}_{𝔥}^{**}}{{Ȿ}_{𝔥}}+{𝔗}_{{Ȿ}_{𝔥}}^{**}\left(\tau +\gamma \right)\;-\delta_{𝔥}{𝔗}_{{Ȿ}_{𝔥}}\left(1-\frac{{𝔗}_{{Ȿ}_{𝔥}}^{**}}{{𝔗}_{{Ȿ}_{𝔥}}}\right)-\alpha_{𝔪}{Ȿ}_{𝔥}\\ \left(\chi_{{Ȿ}_{𝔥}}\frac{{𝔗}_{{Ȿ}_{𝔥}}^{**}}{{𝔗}_{{Ȿ}_{𝔥}}}+\chi_{A_{𝔥}}\frac{{𝔗}_{A_{𝔥}}^{**}}{{\;𝔗}_{A_{𝔥}}}\right){𝔗}_{𝔪}+\left(\varrho +\gamma \right){𝔗}_{A_{𝔥}}^{**}\\ -\delta_{𝔥}{\;𝔗}_{A_{𝔥}}\left(1-\frac{{𝔗}_{A_{𝔥}}^{**}}{{\;𝔗}_{A_{𝔥}}}\right)-\gamma \left({𝔗}_{{Ȿ}_{𝔥}}+{𝔗}_{A_{𝔥}}\right)\\ \frac{{𝔎}_{𝔥}^{**}}{{𝔎}_{𝔥}}+\theta {𝔎}_{𝔥}^{**}-\delta_{𝔥}{𝔎}_{𝔥}\left(1-\frac{{𝔎}_{𝔥}^{**}}{{𝔎}_{𝔥}}\right)+\left(\tau {𝔗}_{{Ȿ}_{𝔥}}+\varrho {\;𝔗}_{A_{𝔥}}\right)\frac{{𝔎}_{𝔥}^{**}}{{𝔎}_{𝔥}}\\ +\pi_{𝔪}\left(1-\frac{{Ȿ}_{𝔪}^{**}}{{Ȿ}_{𝔪}}\right)-\delta_{𝔪}\left(1-\frac{{Ȿ}_{𝔪}^{**}}{{Ȿ}_{𝔪}}\right)-\delta_{𝔪}\left(1-\frac{{𝔗}_{𝔪}^{**}}{{𝔗}_{𝔪}}\right)\\ +\alpha_{𝔪}\chi_{𝔪}\left({𝔗}_{{Ȿ}_{𝔥}}+{\;𝔗}_{A_{𝔥}}\right)\left({Ȿ}_{𝔪}^{**}+{Ȿ}_{𝔪}\frac{{𝔗}_{𝔪}^{**}}{{𝔗}_{𝔪}}\right)\;\\ \begin{array}{c} AℬC\\ 0 \end{array}{𝔇}_{ȶ}^{\eta }{\;𝕍}_{2}\left(ȶ\right)\leq \left(\pi_{𝔥}-\delta_{𝔥}+\theta {𝔎}_{𝔥}\right)\\ \left(2-\frac{{Ȿ}_{𝔥}^{**}}{{Ȿ}_{𝔥}}-\frac{{Ȿ}_{𝔥}}{{Ȿ}_{𝔥}^{**}}\right)+\left(\tau +\gamma -\delta_{𝔥}\right)\left(2-\frac{{𝔗}_{{Ȿ}_{𝔥}}^{**}}{{𝔗}_{{Ȿ}_{𝔥}}}-\frac{{𝔗}_{{Ȿ}_{𝔥}}}{{𝔗}_{{Ȿ}_{𝔥}}^{**}}\right)\\ +\left(\varrho +\gamma -\delta_{𝔥}\right)\;\\ \left(2-\frac{{𝔗}_{A_{𝔥}}^{**}}{{\;𝔗}_{A_{𝔥}}}-\frac{{\;𝔗}_{A_{𝔥}}}{{𝔗}_{A_{𝔥}}^{**}}\right)+\left(\gamma +\delta_{𝔥}-\theta \right)\left(2-\frac{{𝔎}_{𝔥}^{**}}{{𝔎}_{𝔥}}-\frac{{𝔎}_{𝔥}}{{𝔎}_{𝔥}^{**}}\right)\\ +\left(\pi_{𝔪}-\delta_{𝔪}\right)\left(2-\frac{{Ȿ}_{𝔪}^{**}}{{Ȿ}_{𝔪}}\frac{{Ȿ}_{𝔪}}{{Ȿ}_{𝔪}^{**}}\right)+\delta_{𝔪}\left(2-\frac{{𝔗}_{𝔪}^{**}}{{𝔗}_{𝔪}}-\frac{{𝔗}_{𝔪}}{{𝔗}_{𝔪}^{**}}\right)\; \end{array}$$

Hence, the condition in Equation 16 ensures

$\begin{array}{c} AℬC\\ 0 \end{array}{𝔇}_{ȶ}^{\eta }{\;𝕍}_{2}\left(ȶ\right)\leq 0$ for all $\left({Ȿ}_{𝔥}^{**}\;,\;{𝔗}_{{Ȿ}_{𝔥}}^{**},\;{𝔗}_{A_{𝔥}}^{**},{𝔎}_{𝔥}^{**},S_{𝔪}^{**},\;{𝔗}_{𝔪}^{**}\right)\in \Phi \;$ and strict the quality holds for $S_{𝔥}={Ȿ}_{𝔥}={Ȿ}_{𝔥}^{**},{𝔗}_{S_{𝔥}}=\;{𝔗}_{S_{𝔥}}^{**},$ ${𝔗}_{A_{𝔥}}=\;{𝔗}_{A_{𝔥}}^{**},{𝔎}_{𝔥}=\;{𝔎}_{𝔥}^{**},{Ȿ}_{𝔪}={Ȿ}_{𝔪}^{**}$ and ${𝔗}_{𝔪}={𝔗}_{𝔪}^{**}.$ therefore the equilibrium point $C_{p}$ becomes globally stable on Φ.

## 5 Optimum control approach

In this portion, we will discuss how to optimize the problem and analyze the performance of the control function. Consolidation of optimal controlling problem a dynamics of control system can be described as system (Equation 6).


(17) $$\begin{array}{l} \begin{array}{c} AℬC\\ 0 \end{array}{𝔇}_{ȶ}^{\eta }{Ȿ}_{𝔥}=\pi_{𝔥}\;-\alpha_{𝔪}\left(\chi_{{Ȿ}_{𝔥}}+\chi_{A_{𝔥}}\right)S_{𝔥}{𝔗}_{𝔪}\;-\;\delta_{𝔥}S_{𝔥}+\theta {𝔎}_{𝔥}-{𝕌}_{1}\left(ȶ\right)S_{𝔥}\;\\ \begin{array}{c} AℬC\\ 0 \end{array}{𝔇}_{ȶ}^{\eta }{𝔗}_{{Ȿ}_{𝔥}}=\alpha_{𝔪}\chi_{{Ȿ}_{𝔥}}S_{𝔥}{𝔗}_{𝔪}\;-\left(\tau +\gamma +\delta_{𝔥}\right){𝔗}_{S_{𝔥}}\;\\ \begin{array}{c} AℬC\\ 0 \end{array}{𝔇}_{ȶ}^{\eta }{𝔗}_{A_{𝔥}}=\;\alpha_{𝔪}\chi_{A_{𝔥}}{Ȿ}_{𝔥}{𝔗}_{𝔪}\;-\left(\varrho +\gamma +\delta_{𝔥}\right){𝔗}_{A_{𝔥}}\;\\ \begin{array}{c} AℬC\\ 0 \end{array}{𝔇}_{ȶ}^{\eta }{Ȿ}_{𝔪}=\pi_{𝔪}-\alpha_{𝔪}\chi_{𝔪}\left({𝔗}_{{Ȿ}_{𝔥}}+{𝔗}_{A_{𝔥}}\right){Ȿ}_{𝔪}-\delta_{𝔪}{Ȿ}_{𝔪}-{𝕌}_{2}\left(ȶ\right){Ȿ}_{𝔪}\;\\ \begin{array}{c} AℬC\\ 0 \end{array}{𝔇}_{ȶ}^{\eta }{𝔗}_{𝔪}=\;\alpha_{𝔪}\chi_{𝔪}\left({𝔗}_{{Ȿ}_{𝔥}}+{𝔗}_{A_{𝔥}}\right){Ȿ}_{𝔪}-\delta_{𝔪}{𝔗}_{𝔪}-{𝕌}_{2}\left(ȶ\right){𝔗}_{𝔪} \end{array}$$

Where

𝕌_1_− Self-precaution (long sleeved pants and shorts, increase immune system, consultation at

the neatest health care) minimizes the susceptible individuals.

𝕌_2_− Use of chemical insecticide sprays destroying the susceptible and infected mosquito cases

The optimal solution being minimized could be expressed as:


(18) $$\begin{array}{l} \mathbb{C}\left({𝕌}_{1},{𝕌}_{2}\right)=\int_{0}^{{ȶ}_{f}}\left(a{Ȿ}_{𝔥}+b{𝔗}_{{Ȿ}_{𝔥}}+c{𝔗}_{A_{𝔥}}+d{Ȿ}_{𝔪}+e{𝔗}_{𝔪}+f{{𝕌}_{1}}^{2}+g{{𝕌}_{2}}^{2}\right)dȶ \end{array}$$

To reduce the cost of two controls 𝕌_1_ and 𝕌_2_ the objective is reduced ${\;Ȿ}_{𝔥}\;,\;{𝔗}_{S_{𝔥}},\;{𝔗}_{A_{𝔥}}$ and *S*_𝔪_, 𝔗_𝔪_.

Therefore, we need to obtain optimal controls ${𝕌}_{1}^{*}$ and ${𝕌}_{2}^{*}$


(19) $$\begin{array}{l} \mathbb{C}\left({𝕌}_{1}^{*},{𝕌}_{2}^{*}\right)=\min_{{𝕌}_{1},{𝕌}_{2}}\left\{\mathbb{C}\left({𝕌}_{1},{𝕌}_{2}\right)|{𝕌}_{1},{𝕌}_{2}\in \Phi \right\} \end{array}$$

A set of constraints $\Phi =\left\{\left({𝕌}_{1},\;{𝕌}_{2}\right)|{𝕌}_{i}\;:\left[0,{ȶ}_{f}\right]\to \left[0,\infty \right)lebesque\;quantifiable\;i=1,2\;\right\}$.

The expense of minimizing ${Ȿ}_{𝔥},\;{𝔗}_{{Ȿ}_{𝔥}},\;{𝔗}_{A_{𝔥}}$, Ȿ_𝔪_ and 𝔗_𝔪_ is represented by the term $a{Ȿ}_{𝔥},\;b{𝔗}_{{Ȿ}_{𝔥}},\;c{𝔗}_{A_{𝔥}},\;d{Ȿ}_{𝔪}$ and $e{𝔗}_{𝔪}$ respectively. Likewise, $f{𝕌}_{1}^{2},g{𝕌}_{2}^{2}$ represents the cost for controls 𝕌_1_, 𝕌_2_. The most prevalent PMP can be used to find the adequacy condition required for the control system to be satisfied. Equations 17, 19 can be transformed into the following point-wise Hamiltonian ℍ for (𝕌_1_, 𝕌_2_) regression problem using the aforesaid principle.


(20) $$\begin{array}{l} ℍ=\left\{a{Ȿ}_{𝔥}+b{𝔗}_{S_{𝔥}}+c{𝔗}_{A_{𝔥}}+dS_{𝔪}+e{𝔗}_{𝔪}+f{{𝕌}_{1}}^{2}+g{{𝕌}_{2}}^{2}\right\}\\ +\lambda_{{Ȿ}_{𝔥}}\left\{\pi_{𝔥}-\alpha_{𝔪}\left(\chi_{S_{𝔥}}+\chi_{A_{𝔥}}\right){Ȿ}_{𝔥}{𝔗}_{𝔪}-\delta_{𝔥}{Ȿ}_{𝔥}+\theta {𝔎}_{𝔥}-{𝕌}_{1}\left(ȶ\right){Ȿ}_{𝔥}\right\}\\ +\;\lambda_{{𝔗}_{{Ȿ}_{𝔥}}}\left\{\alpha_{𝔪}\chi_{{Ȿ}_{𝔥}}S_{𝔥}{𝔗}_{𝔪}-\left(\tau +\gamma +\delta_{𝔥}\right){𝔗}_{S_{𝔥}}\right\}\\ +\lambda_{{𝔗}_{A_{𝔥}}}\left\{\alpha_{𝔪}\chi_{A_{𝔥}}{Ȿ}_{𝔥}{𝔗}_{𝔪}\;-\left(\varrho +\gamma +\delta_{𝔥}\right){𝔗}_{A_{𝔥}}\right\}\\ +\;\lambda_{{Ȿ}_{𝔪}}\left\{\pi_{𝔪}-\alpha_{𝔪}\chi_{𝔪}\left({𝔗}_{S_{𝔥}}+{𝔗}_{A_{𝔥}}\right){Ȿ}_{𝔪}-\delta_{𝔪}S_{𝔪}-{𝕌}_{2}\left(ȶ\right)S_{𝔪}\right\}\\ +\lambda_{{𝔗}_{𝔪}}\left\{\alpha_{𝔪}\chi_{𝔪}\left({𝔗}_{{Ȿ}_{𝔥}}+{𝔗}_{A_{𝔥}}\right){Ȿ}_{𝔪}-\delta_{𝔪}{𝔗}_{𝔪}-{𝕌}_{2}\left(ȶ\right){𝔗}_{𝔪}\right\}\; \end{array}$$

Where $\lambda_{{Ȿ}_{𝔥}},\lambda_{{𝔗}_{{Ȿ}_{𝔥}}},\;\lambda_{{𝔗}_{A_{𝔥}}},\;\lambda_{S_{m}}$ and λ_𝔗_𝔪__ are the ad-joint variable or co-state variable.


(21) $$\begin{array}{l} \frac{d\lambda_{{Ȿ}_{𝔥}}}{dȶ}=\frac{\partial ℍ}{\partial {Ȿ}_{𝔥}}=a+\lambda_{{Ȿ}_{𝔥}}\left\{-\alpha_{𝔪}\left(\chi_{S_{𝔥}}+\chi_{A_{𝔥}}\right){𝔗}_{𝔪}-\delta_{𝔥}\right\}\\ -\left(\lambda_{{𝔗}_{{Ȿ}_{𝔥}}}+\lambda_{{𝔗}_{A_{𝔥}}}\right)\alpha_{𝔪}\left(\chi_{{Ȿ}_{𝔥}}+\chi_{A_{𝔥}}\right){𝔗}_{𝔪}\;\\ \frac{d\lambda_{{𝔗}_{{Ȿ}_{𝔥}}}}{dȶ}=\frac{\partial ℍ}{\partial {𝔗}_{{Ȿ}_{𝔥}}}=b-\lambda_{{Ȿ}_{𝔥}}{𝕌}_{1}\left(ȶ\right)+\lambda_{{𝔗}_{{Ȿ}_{𝔥}}}\left\{-\left(\tau +\gamma +\delta_{𝔥}\right)\right\}\\ +\lambda_{{𝔗}_{𝔪}}\alpha_{𝔪}\chi_{𝔪}{Ȿ}_{𝔪}\;\\ \frac{d\lambda_{{𝔗}_{A_{𝔥}}}}{dȶ}=\frac{\partial ℍ}{\partial {𝔗}_{A_{𝔥}}}=c-\lambda_{{Ȿ}_{𝔥}}{𝕌}_{1}\left(ȶ\right)\\ +\lambda_{{𝔗}_{A_{𝔥}}}\left\{-\left(\varrho +\gamma +\delta_{𝔥}\right)\right\}+\lambda_{{𝔗}_{𝔪}}\alpha_{𝔪}\chi_{𝔪}{Ȿ}_{𝔪}\\ \frac{d\lambda_{{Ȿ}_{𝔪}}}{dȶ}=\frac{\partial ℍ}{\partial {Ȿ}_{𝔪}}\\ =d+\lambda_{{Ȿ}_{𝔪}}\left\{-\alpha_{𝔪}\chi_{𝔪}\left({𝔗}_{{Ȿ}_{𝔥}}+{𝔗}_{A_{𝔥}}\right)-\delta_{𝔪}-{𝕌}_{2}\left(ȶ\right)\right\}\\ +\lambda_{{𝔗}_{𝔪}}\left(\alpha_{𝔪}\chi_{𝔪}\left({𝔗}_{{Ȿ}_{𝔥}}+{𝔗}_{A_{𝔥}}\right)\right)\;\\ \frac{d\lambda_{{𝔗}_{𝔪}}}{dȶ}=\frac{\partial ℍ}{\partial {𝔗}_{𝔪}}=e+\lambda_{{𝔗}_{𝔪}}\{-\delta_{𝔪}-{𝕌}_{2}\left(ȶ\right)\}\\ -\left(\lambda_{{𝔗}_{{Ȿ}_{𝔥}}}+\lambda_{{𝔗}_{A_{𝔥}}}\right)\alpha_{𝔪}\left(\chi_{{Ȿ}_{𝔥}}+\chi_{A_{𝔥}}\right)S_{𝔥}+\lambda_{S_{𝔥}}\\ \left\{-\alpha_{𝔪}\left(\chi_{{Ȿ}_{𝔥}}+\chi_{A_{𝔥}}\right){Ȿ}_{𝔥}\;\right\} \end{array}$$

The conditions for transversality are

$\lambda_{{Ȿ}_{𝔥}}\left({ȶ}_{f}\right)=\lambda_{{𝔗}_{{Ȿ}_{𝔥}}}\left(ȶ\right)=\lambda_{{𝔗}_{A_{𝔥}}}\left({ȶ}_{f}\right)=\;\lambda_{{Ȿ}_{𝔪}}\left({ȶ}_{f}\right)=\lambda_{{𝔗}_{𝔪}}\left({ȶ}_{f}\right)=\;0.$

For $0<{𝕌}_{i}<1,\;i=1\;,2.$ From the interior of controls, we have:


(22) $$\begin{array}{l} \frac{\partial ℍ}{\partial {𝕌}_{1}}=2f{𝕌}_{1}-\lambda_{{Ȿ}_{𝔥}}\left({𝔗}_{S_{𝔥}}+{𝔗}_{A_{𝔥}}\right)\;\\ \frac{\partial ℍ}{\partial {𝕌}_{2}}=2g{𝕌}_{2}-\lambda_{{Ȿ}_{𝔪}}{Ȿ}_{𝔪}-\lambda_{{𝔗}_{𝔪}}{𝔗}_{𝔪}\; \end{array}$$

From where:


(23) $$\begin{array}{l} {𝕌}_{1}=\;\frac{\lambda_{{Ȿ}_{𝔥}}\left({𝔗}_{{Ȿ}_{𝔥}}+{𝔗}_{A_{𝔥}}\right)}{2f}\;\\ {𝕌}_{2}=\frac{\lambda_{{Ȿ}_{𝔪}}{Ȿ}_{𝔪}+\lambda_{{𝔗}_{𝔪}}{𝔗}_{𝔪}}{2g} \end{array}$$

### 5.1 Utilization of optimal solutions

**Theorem 5.1**. $\left({𝕌}_{1}^{*},{𝕌}_{2}^{*}\right)$ is a control factor can reduce over 𝕌 provided by


(24) $$\begin{array}{l} {𝕌}_{1}^{*}=max\;\left\{0,min\left\{1,{\frac{1}{2f}\lambda }_{{Ȿ}_{𝔥}}\left({𝔗}_{{Ȿ}_{𝔥}}+{𝔗}_{A_{𝔥}}\right)\right\}\right\}\;\\ {𝕌}_{2}^{*}=max\left\{0,min\left\{1,\frac{1}{2g}\lambda_{{Ȿ}_{𝔪}}{Ȿ}_{𝔪}+\lambda_{{𝔗}_{𝔪}}{𝔗}_{𝔪}\right\}\right\}\; \end{array}$$

Where $\lambda_{{Ȿ}_{𝔥}},\lambda_{{𝔗}_{{Ȿ}_{𝔥}}},\;\lambda_{{𝔗}_{A_{𝔥}}},\;\lambda_{{Ȿ}_{𝔪}}$ and λ_𝔗_𝔪__ are co-state variable that satisfy the condition (Equations 17–24) in addition, the transversality characteristic that follows


$$\begin{array}{l} \lambda_{{Ȿ}_{𝔥}}\left({ȶ}_{f}\right)=\lambda_{{𝔗}_{{Ȿ}_{𝔥}}}\left({ȶ}_{f}\right)=\lambda_{{𝔗}_{A_{𝔥}}}\left({ȶ}_{f}\right)=\;\lambda_{{Ȿ}_{𝔪}}\left({ȶ}_{f}\right)=\lambda_{{𝔗}_{𝔪}}\left({ȶ}_{f}\right)=0.\; \end{array}$$

$$\begin{array}{l} {𝕌}_{1}^{*}=\{\begin{array}{c} 0\;if\;{𝕌}_{1}\leq 0,\;\\ {𝕌}_{1}\;if\;0<{𝕌}_{1}<1,\\ 1\;if\;{𝕌}_{1}\geq 0. \end{array} \end{array}$$

And


(25) $$\begin{array}{l} {𝕌}_{2}^{*}=\{\begin{array}{c} 0\;if\;{𝕌}_{2}\leq 0\;,\\ {𝕌}_{2}\;if\;0<{𝕌}_{2}<1,\\ 1\;if\;{𝕌}_{2}\geq 0. \end{array} \end{array}$$

**Proof**

To demonstrate the survival of optimal control solutions, the configuration of the Lipschitz criterion of the system and the convexity of the integral in Equation 21 are related and state variable that constrains 𝕌_1_ and 𝕌_2_ to the boundary of the state solution. So we employ PMP and get the following:


(26) $$\begin{array}{l} \begin{array}{c} AℬC\\ 0 \end{array}{𝔇}_{ȶ}^{\eta }\lambda_{{Ȿ}_{𝔥}}\left(ȶ\right)=\frac{\partial ℍ}{\partial {Ȿ}_{𝔥}};\\ \begin{array}{c} AℬC\\ 0 \end{array}{𝔇}_{ȶ}^{\eta }\lambda_{{𝔗}_{{Ȿ}_{𝔥}}}\left(ȶ\right)=\frac{\partial ℍ}{\partial {𝔗}_{{Ȿ}_{𝔥}}};\begin{array}{c} AℬC\\ 0 \end{array}{𝔇}_{ȶ}^{\eta }\lambda_{{𝔗}_{A_{𝔥}}}\left(ȶ\right)=\frac{\partial ℍ}{\partial {𝔗}_{A_{𝔥}}};\;\\ \begin{array}{c} AℬC\\ 0 \end{array}{𝔇}_{ȶ}^{\eta }\lambda_{{Ȿ}_{𝔪}}\left(ȶ\right)=\frac{\partial ℍ}{\partial {Ȿ}_{𝔪}};\begin{array}{c} AℬC\\ 0 \end{array}{𝔇}_{ȶ}^{\eta }\lambda_{{𝔗}_{𝔪}}\left(ȶ\right)=\frac{\partial ℍ}{\partial {𝔗}_{𝔪}}; \end{array}$$

with,

$\lambda_{{Ȿ}_{𝔥}}\left({ȶ}_{f}\right)=\lambda_{{𝔗}_{{Ȿ}_{𝔥}}}\left({ȶ}_{f}\right)=\lambda_{{𝔗}_{A_{𝔥}}}\left({ȶ}_{f}\right)=\;\lambda_{{Ȿ}_{𝔪}}\left({ȶ}_{f}\right)=\lambda_{{𝔗}_{𝔪}}\left({ȶ}_{f}\right)=\;0.$

The Hamilton can be differentiated with regard to achieve the conditional optimum:


(27) $$\begin{array}{l} \frac{\partial ℍ}{\partial {𝕌}_{1}}=0,\frac{\partial ℍ}{\partial {𝕌}_{2\;}}=0.\; \end{array}$$

The ad-joint system (Equations 20, 21) derived from Equation 17, the optimum system (Equation 23) is accessible from Equation 24. The optimal method is the constrained system (Equation 17) and its initial state is ad-joint the system includes (Equation 20), and condition for intersection.

## 6 Adams-Bash forth method

Here, we formulate the system of Equation 6 a recently invented numerical approach, the Adams-Bash forth method (24). The framework (Equation 6) can be used to test the essential theorem from fractional calculus,


$$\begin{array}{l} {Ȿ}_{𝔥}\left(ȶ\right)\;={Ȿ}_{𝔥}\left(0\right)+\frac{1-\eta }{\mathrm{ABC}\left(\eta \right)}{𝕂}_{1}(ȶ,\;{Ȿ}_{𝔥}\left(ȶ\right),{𝔗}_{{Ȿ}_{𝔥}}\left(ȶ\right),\;{\;𝔗}_{A_{𝔥}}\left(ȶ\right),\\ {𝔎}_{𝔥}\left(ȶ\right),\;{Ȿ}_{𝔪}\left(ȶ\right),{𝔗}_{𝔪}\left(ȶ\right))+\;\\ \frac{\eta }{\mathrm{ABC}\left(\eta \right)\lceil \eta }\int_{0}^{ȶ}{𝕂}_{1}(\varpi ,\;{Ȿ}_{𝔥}\left(\varpi \right),{𝔗}_{S_{𝔥}}\left(\varpi \right),\\ {𝔗}_{A_{𝔥}}\left(\varpi \right), \end{array}$$

(28) $$\begin{array}{l} {𝔎}_{𝔥}\left(\varpi \right),{Ȿ}_{𝔪}\left(\varpi \right),{𝔗}_{𝔪}\left(\varpi \right)){\left(ȶ-\varpi \right)}^{\eta -1}d\varpi \end{array}$$

$$\begin{array}{l} {𝔗}_{{Ȿ}_{𝔥}}\left(ȶ\right)={𝔗}_{{Ȿ}_{𝔥}}\left(0\right)+\frac{1-\eta }{\mathrm{ABC}\left(\eta \right)}{𝕂}_{2}(ȶ,\;{Ȿ}_{𝔥}\left(ȶ\right),{𝔗}_{{Ȿ}_{𝔥}}\left(ȶ\right),\;{\;𝔗}_{A_{𝔥}}\left(ȶ\right),\\ {𝔎}_{𝔥}\left(ȶ\right),\;{Ȿ}_{𝔪}\left(ȶ\right),{𝔗}_{𝔪}\left(ȶ\right))+\;\\ \frac{\eta }{\mathrm{ABC}\left(\eta \right)\lceil \eta }\int_{0}^{ȶ}{𝕂}_{2}(\varpi ,\;{Ȿ}_{𝔥}\left(\varpi \right),{𝔗}_{{Ȿ}_{𝔥}}\left(\varpi \right),\;{\;𝔗}_{A_{𝔥}}\left(\varpi \right),{𝔎}_{𝔥} \end{array}$$

(29) $$\begin{array}{l} \left(\varpi \right),{Ȿ}_{𝔪}\left(\varpi \right),{𝔗}_{𝔪}\left(\varpi \right)){\left(ȶ-\varpi \right)}^{\eta -1}d\varpi \end{array}$$

$$\begin{array}{l} {\;𝔗}_{A_{𝔥}}\left(ȶ\right)\;={\;𝔗}_{A_{𝔥}}\left(0\right)+\frac{1-\eta }{\mathrm{ABC}\left(\eta \right)}{𝕂}_{3}(ȶ,\;{Ȿ}_{𝔥}\left(ȶ\right),{𝔗}_{{Ȿ}_{𝔥}}\left(ȶ\right),\;{\;𝔗}_{A_{𝔥}}\left(ȶ\right),\\ {𝔎}_{𝔥}\left(ȶ\right),\;{Ȿ}_{𝔪}\left(ȶ\right),{𝔗}_{𝔪}\left(ȶ\right))+\\ \frac{\eta }{\mathrm{ABC}\left(\eta \right)\lceil \eta }\int_{0}^{ȶ}{𝕂}_{3}(\varpi ,\;{Ȿ}_{𝔥}\left(\varpi \right),\\ T_{{Ȿ}_{h}}\left(\varpi \right),\;T_{A_{h}}\left(\varpi \right),K_{h}\left(\varpi \right), \end{array}$$

(30) $$\begin{array}{l} {Ȿ}_{𝔪}\left(\varpi \right),{𝔗}_{𝔪}\left(\varpi \right)){\left(ȶ-\varpi \right)}^{\eta -1}d\varpi \end{array}$$

(31) $$\begin{array}{l} {𝔎}_{𝔥}\left(ȶ\right)\;=\;{𝔎}_{𝔥}\left(0\right)+\frac{1-\eta }{\mathrm{ABC}\left(\eta \right)}{𝕂}_{4}(ȶ,\;{Ȿ}_{𝔥}\left(ȶ\right),{𝔗}_{{Ȿ}_{𝔥}}\left(ȶ\right),\\ {\;𝔗}_{A_{𝔥}}\left(ȶ\right),{𝔎}_{𝔥}\left(ȶ\right),\;{Ȿ}_{𝔪}\left(ȶ\right),{𝔗}_{𝔪}\left(ȶ\right))+\;\\ \frac{\eta }{\mathrm{ABC}\left(\eta \right)\lceil \eta }\int_{0}^{ȶ}{𝕂}_{4}(\varpi ,\;{Ȿ}_{𝔥}\left(\varpi \right),{𝔗}_{S_{𝔥}}\left(\varpi \right),\\ {𝔗}_{A_{𝔥}}\left(\varpi \right),\\ {𝔎}_{𝔥}\left(\varpi \right),{Ȿ}_{𝔪}\left(\varpi \right),{𝔗}_{𝔪}\left(\varpi \right)){\left(ȶ-\varpi \right)}^{\eta -1}d\varpi \end{array}$$

$$\begin{array}{l} {Ȿ}_{𝔪}\left(ȶ\right)\;=\;{Ȿ}_{𝔪}\left(0\right)+\frac{1-\eta }{\mathrm{ABC}\left(\eta \right)}{𝕂}_{5}(ȶ,\;{Ȿ}_{𝔥}\left(ȶ\right),\\ {𝔗}_{S_{𝔥}}\left(ȶ\right),\;{\;𝔗}_{A_{𝔥}}\left(ȶ\right),{𝔎}_{𝔥}\left(ȶ\right),{Ȿ}_{𝔪}\left(ȶ\right),{𝔗}_{𝔪}\left(ȶ\right))+\;\\ \frac{\eta }{\mathrm{ABC}\left(\eta \right)\lceil \eta }\int_{0}^{ȶ}{𝕂}_{5}(\varpi ,\;{Ȿ}_{𝔥}\left(\varpi \right),{𝔗}_{S_{𝔥}}\left(\varpi \right),\\ {\;𝔗}_{A_{𝔥}}\left(\varpi \right), \end{array}$$

(32) $$\begin{array}{l} {𝔎}_{𝔥}\left(\varpi \right),{Ȿ}_{𝔪}\left(\varpi \right),{𝔗}_{𝔪}\left(\varpi \right)){\left(ȶ-\varpi \right)}^{\eta -1}d\varpi \end{array}$$

(33) $$\begin{array}{l} {𝔗}_{𝔪}\left(ȶ\right)\;=\;{𝔗}_{𝔪}\left(0\right)+\frac{1-\eta }{ABC\left(\eta \right)}{𝕂}_{6}(ȶ,\;{Ȿ}_{𝔥}\left(ȶ\right),{𝔗}_{{Ȿ}_{𝔥}}\left(ȶ\right),\\ {\;𝔗}_{A_{𝔥}}\left(ȶ\right),{𝔎}_{𝔥}\left(ȶ\right),\;{Ȿ}_{𝔪}\left(ȶ\right),{𝔗}_{𝔪}\left(ȶ\right))+\;\\ \frac{\eta }{\mathrm{ABC}\left(\eta \right)\lceil \eta }\int_{0}^{ȶ}{𝕂}_{6}(\varpi ,\;{Ȿ}_{𝔥}\left(\varpi \right),{𝔗}_{{Ȿ}_{𝔥}}\left(\varpi \right),\\ {\;𝔗}_{A_{𝔥}}\left(\varpi \right),{𝔎}_{𝔥}\left(\varpi \right),{Ȿ}_{𝔪}\left(\varpi \right),{𝔗}_{𝔪}\left(\varpi \right)){\left(ȶ-\varpi \right)}^{\eta -1}d\varpi \; \end{array}$$

Where,


(34) $$\begin{array}{l} \{\begin{array}{c} {𝕂}_{1}\left(ȶ,\;{Ȿ}_{𝔥}\left(ȶ\right),{𝔗}_{{Ȿ}_{𝔥}}\left(ȶ\right),\;{\;𝔗}_{A_{𝔥}}\left(ȶ\right),{𝔎}_{𝔥}\left(ȶ\right),\;{Ȿ}_{𝔪}\left(ȶ\right),{𝔗}_{𝔪}\left(ȶ\right)\right)\\ =\pi_{𝔥}\;-\alpha_{𝔪}\left(\chi_{{Ȿ}_{𝔥}}+\chi_{A_{𝔥}}\right)S_{𝔥}{𝔗}_{𝔪}\;-\;\delta_{𝔥}{Ȿ}_{𝔥}+\theta {𝔎}_{𝔥}\\ {𝕂}_{2}\left(ȶ,\;S_{𝔥}\left(ȶ\right),{𝔗}_{S_{𝔥}}\left(ȶ\right),\;{\;𝔗}_{A_{𝔥}}\left(ȶ\right),{𝔎}_{𝔥}\left(ȶ\right),\;S_{𝔪}\left(ȶ\right),{𝔗}_{𝔪}\left(ȶ\right)\right)\\ =\alpha_{𝔪}\chi_{{Ȿ}_{𝔥}}{Ȿ}_{𝔥}{𝔗}_{𝔪}\;-\left(\tau +\gamma +\delta_{𝔥}\right){𝔗}_{S_{𝔥}}\\ {𝕂}_{3}\left(ȶ,\;S_{𝔥}\left(ȶ\right),{𝔗}_{S_{𝔥}}\left(ȶ\right),\;{\;𝔗}_{A_{𝔥}}\left(ȶ\right),{𝔎}_{𝔥}\left(ȶ\right),\;S_{𝔪}\left(ȶ\right),{𝔗}_{𝔪}\left(ȶ\right)\right)\\ =\alpha_{𝔪}\chi_{A_{𝔥}}{Ȿ}_{𝔥}{𝔗}_{𝔪}\;-\left(\varrho +\gamma +\delta_{𝔥}\right){\;𝔗}_{A_{𝔥}}\\ {𝕂}_{4}\left(ȶ,\;S_{𝔥}\left(ȶ\right),{𝔗}_{S_{𝔥}}\left(ȶ\right),\;{\;𝔗}_{A_{𝔥}}\left(ȶ\right),{𝔎}_{𝔥}\left(ȶ\right),\;S_{𝔪}\left(ȶ\right),{𝔗}_{𝔪}\left(ȶ\right)\right)\\ =\gamma \left({𝔗}_{{Ȿ}_{𝔥}}+{\;𝔗}_{A_{𝔥}}\right)-\theta {𝔎}_{𝔥}-\delta_{𝔥}{𝔎}_{𝔥}+\tau {𝔗}_{{Ȿ}_{𝔥}}+\varrho {\;𝔗}_{A_{𝔥}}\\ {𝕂}_{5}\left(ȶ,\;{Ȿ}_{𝔥}\left(ȶ\right),{𝔗}_{{Ȿ}_{𝔥}}\left(ȶ\right),\;{\;𝔗}_{A_{𝔥}}\left(ȶ\right),{𝔎}_{𝔥}\left(ȶ\right),\;{Ȿ}_{𝔪}\left(ȶ\right),{𝔗}_{𝔪}\left(ȶ\right)\right)\\ =\pi_{𝔪}-\alpha_{𝔪}\chi_{𝔪}\left({𝔗}_{{Ȿ}_{𝔥}}+{\;𝔗}_{A_{𝔥}}\right){Ȿ}_{𝔪}-\Omega {Ȿ}_{𝔪}-\delta_{𝔪}{Ȿ}_{𝔪}\\ {𝕂}_{6}\left(ȶ,\;S_{𝔥}\left(ȶ\right),{𝔗}_{S_{𝔥}}\left(ȶ\right),\;{\;𝔗}_{A_{𝔥}}\left(ȶ\right),{𝔎}_{𝔥}\left(ȶ\right),\;S_{𝔪}\left(ȶ\right),{𝔗}_{𝔪}\left(ȶ\right)\right)\\ =\alpha_{𝔪}\chi_{𝔪}{\left({𝔗}_{{Ȿ}_{𝔥}}+{\;𝔗}_{A_{𝔥}}\right)Ȿ}_{m}-\left(\Omega +\delta_{𝔪}\right){𝔗}_{𝔪} \end{array} \end{array}$$

The following structure is obtained at time *t*_𝔫+ 1_,


$$\begin{array}{l} {Ȿ}_{𝔥}\left({ȶ}_{𝔫+1}\right)\;={Ȿ}_{𝔥}\left(0\right)+\frac{1-\eta }{\mathrm{ABC}\left(\eta \right)}{𝕂}_{1}\\ \left({ȶ}_{𝔫},\;{Ȿ}_{{𝔥}_{𝔫}},{𝔗}_{{Ȿ}_{{𝔥}_{𝔫}}},\;{\;𝔗}_{A_{{𝔥}_{𝔫}}},{𝔎}_{{𝔥}_{𝔫}},\;{Ȿ}_{{𝔪}_{𝔫}},{𝔗}_{{𝔪}_{𝔫}}\right)+\;\\ \frac{\eta }{\mathrm{ABC}\left(\eta \right)\lceil \eta }\int_{0}^{{ȶ}_{𝔫+1}}{𝕂}_{1}(ȶ,\;{Ȿ}_{𝔥}\left(ȶ\right),{𝔗}_{{Ȿ}_{𝔥}}\left(ȶ\right),\;{\;𝔗}_{A_{𝔥}}\left(ȶ\right),{𝔎}_{𝔥}\left(ȶ\right), \end{array}$$

(35) $$\begin{array}{l} {Ȿ}_{𝔪}\left(ȶ\right),{𝔗}_{𝔪}\left(ȶ\right)){\left({ȶ}_{𝔫+1}-t\right)}^{\eta -1}dȶ \end{array}$$

$$\begin{array}{l} {𝔗}_{{Ȿ}_{𝔥}}\left({ȶ}_{𝔫+1}\right)\;={𝔗}_{{Ȿ}_{𝔥}}\left(0\right)+\frac{1-\eta }{\mathrm{ABC}\left(\eta \right)}\\ {𝕂}_{2}\left({ȶ}_{𝔫},\;{Ȿ}_{{𝔥}_{𝔫}},{𝔗}_{{Ȿ}_{{𝔥}_{𝔫}}},\;{\;𝔗}_{A_{{𝔥}_{𝔫}}},{𝔎}_{{𝔥}_{𝔫}},\;{Ȿ}_{{𝔪}_{𝔫}},{𝔗}_{{𝔪}_{𝔫}}\right)+\;\\ \frac{\eta }{\mathrm{ABC}\left(\eta \right)\lceil \eta }\int_{0}^{{ȶ}_{𝔫+1}}{𝕂}_{2}(ȶ,\;{Ȿ}_{𝔥}\left(ȶ\right),{𝔗}_{{Ȿ}_{𝔥}}\left(ȶ\right),\;{\;𝔗}_{A_{𝔥}}\left(ȶ\right),{𝔎}_{𝔥}\left(ȶ\right), \end{array}$$

(36) $$\begin{array}{l} {Ȿ}_{𝔪}\left(ȶ\right),{𝔗}_{𝔪}\left(ȶ\right)){\left({ȶ}_{𝔫+1}-t\right)}^{\eta -1}dȶ \end{array}$$

$$\begin{array}{l} {𝔗}_{A_{𝔥}}\left({ȶ}_{𝔫+1}\right)\;={\;𝔗}_{A_{𝔥}}\left(0\right)+\frac{1-\eta }{\mathrm{ABC}\left(\eta \right)}\\ {𝕂}_{3}\left({ȶ}_{𝔫},\;{Ȿ}_{{𝔥}_{𝔫}},{𝔗}_{{Ȿ}_{{𝔥}_{𝔫}}},\;{\;𝔗}_{A_{{𝔥}_{𝔫}}},{𝔎}_{{𝔥}_{𝔫}},\;{Ȿ}_{{𝔪}_{𝔫}},{𝔗}_{{𝔪}_{𝔫}}\right)+\;\\ \frac{\eta }{\mathrm{ABC}\left(\eta \right)\lceil \eta }\int_{0}^{{ȶ}_{𝔫+1}}{𝕂}_{3}(ȶ,\;{Ȿ}_{𝔥}\left(ȶ\right),{𝔗}_{{Ȿ}_{𝔥}}\left(ȶ\right),\;{\;𝔗}_{A_{𝔥}}\left(ȶ\right),{𝔎}_{𝔥}\left(ȶ\right), \end{array}$$

(37) $$\begin{array}{l} {Ȿ}_{𝔪}\left(ȶ\right),{𝔗}_{𝔪}\left(ȶ\right)){\left({ȶ}_{𝔫+1}-t\right)}^{\eta -1}dȶ\; \end{array}$$

$$\begin{array}{l} {𝔎}_{𝔥}\left({ȶ}_{𝔫+1}\right)\;=\;{𝔎}_{𝔥}\left(0\right)+\frac{1-\eta }{\mathrm{ABC}\left(\eta \right)}\\ {𝕂}_{4}\left({ȶ}_{𝔫},\;{Ȿ}_{{𝔥}_{𝔫}},{𝔗}_{{Ȿ}_{{𝔥}_{𝔫}}},\;{\;𝔗}_{A_{{𝔥}_{𝔫}}},{𝔎}_{{𝔥}_{𝔫}},\;{Ȿ}_{{𝔪}_{𝔫}},{𝔗}_{{𝔪}_{𝔫}}\right)+\;\\ \frac{\eta }{\mathrm{ABC}\left(\eta \right)\lceil \eta }\int_{0}^{{ȶ}_{𝔫+1}}{𝕂}_{4}(ȶ,\;{Ȿ}_{𝔥}\left(ȶ\right),{𝔗}_{{Ȿ}_{𝔥}}\left(ȶ\right),\;{\;𝔗}_{A_{𝔥}}\left(ȶ\right),{𝔎}_{𝔥}\left(ȶ\right), \end{array}$$

(38) $$\begin{array}{l} {Ȿ}_{𝔪}\left(ȶ\right),{𝔗}_{𝔪}\left(ȶ\right)){\left({ȶ}_{𝔫+1}-t\right)}^{\eta -1}dȶ\; \end{array}$$

$$\begin{array}{l} {Ȿ}_{𝔪}\left({ȶ}_{𝔫+1}\right)\;=\;{Ȿ}_{𝔪}\left(0\right)+\frac{1-\eta }{\mathrm{ABC}\left(\eta \right)}\\ {𝕂}_{5}\left({ȶ}_{𝔫},\;{Ȿ}_{{𝔥}_{𝔫}},{𝔗}_{{Ȿ}_{{𝔥}_{𝔫}}},\;{\;𝔗}_{A_{{𝔥}_{𝔫}}},{𝔎}_{{𝔥}_{𝔫}},\;{Ȿ}_{{𝔪}_{𝔫}},{𝔗}_{{𝔪}_{𝔫}}\right)+\;\\ \frac{\eta }{\mathrm{ABC}\left(\eta \right)\lceil \eta }\int_{0}^{{ȶ}_{𝔫+1}}{𝕂}_{5}(ȶ,\;{Ȿ}_{𝔥}\left(ȶ\right),{𝔗}_{{Ȿ}_{𝔥}}\left(ȶ\right),\;{\;𝔗}_{A_{𝔥}}\left(ȶ\right),{𝔎}_{𝔥}\left(ȶ\right), \end{array}$$

(39) $$\begin{array}{l} {Ȿ}_{𝔪}\left(ȶ\right),{𝔗}_{𝔪}\left(ȶ\right)){\left({ȶ}_{𝔫+1}-t\right)}^{\eta -1}dȶ \end{array}$$

$$\begin{array}{l} {𝔗}_{𝔪}\left({ȶ}_{𝔫+1}\right)\;=\;{𝔗}_{𝔪}\left(0\right)+\frac{1-\eta }{\mathrm{ABC}\left(\eta \right)}\\ {𝕂}_{6}\left({ȶ}_{𝔫},\;{Ȿ}_{{𝔥}_{𝔫}},{𝔗}_{{Ȿ}_{{𝔥}_{𝔫}}},\;{\;𝔗}_{A_{{𝔥}_{𝔫}}},{𝔎}_{{𝔥}_{𝔫}},\;{Ȿ}_{{𝔪}_{𝔫}},{𝔗}_{{𝔪}_{𝔫}}\right)+\;\\ \frac{\eta }{\mathrm{ABC}\left(\eta \right)\lceil \eta }\int_{0}^{{ȶ}_{𝔫+1}}{𝕂}_{6}(ȶ,\;{Ȿ}_{𝔥}\left(ȶ\right),{𝔗}_{{Ȿ}_{𝔥}}\left(ȶ\right),\;{\;𝔗}_{A_{𝔥}}\left(ȶ\right),{𝔎}_{𝔥}\left(ȶ\right), \end{array}$$

(40) $$\begin{array}{l} {Ȿ}_{𝔪}\left(ȶ\right),{𝔗}_{𝔪}\left(ȶ\right)){\left({ȶ}_{𝔫+1}-t\right)}^{\eta -1}dȶ\; \end{array}$$

While, at *t*_𝔫_ we have


$$\begin{array}{l} {Ȿ}_{𝔥}\left({ȶ}_{𝔫}\right)\;=S_{𝔥}\left(0\right)+\frac{1-\eta }{\mathrm{ABC}\left(\eta \right)}{𝕂}_{1}({ȶ}_{𝔫-1},\;{Ȿ}_{{𝔥}_{𝔫-1}},{𝔗}_{{Ȿ}_{{𝔥}_{𝔫-1}}},\;{\;𝔗}_{A_{{𝔥}_{𝔫-1}}},\\ {𝔎}_{{𝔥}_{𝔫-1}},\;{Ȿ}_{{𝔪}_{𝔫-1}},{𝔗}_{{𝔪}_{𝔫-1}})+\\ \frac{\eta }{\mathrm{ABC}\left(\eta \right)\lceil \eta }\int_{0}^{{ȶ}_{𝔫}}{𝕂}_{1}(ȶ,\;{Ȿ}_{𝔥}\left(ȶ\right),{𝔗}_{{Ȿ}_{𝔥}}\left(ȶ\right),\;{\;𝔗}_{A_{𝔥}}\left(ȶ\right),{𝔎}_{𝔥}\left(ȶ\right), \end{array}$$

(41) $$\begin{array}{l} {Ȿ}_{𝔪}\left(ȶ\right),{𝔗}_{𝔪}\left(ȶ\right)){\left({ȶ}_{𝔫}-t\right)}^{\eta -1}dȶ \end{array}$$

$$\begin{array}{l} {𝔗}_{{Ȿ}_{𝔥}}\left({ȶ}_{𝔫}\right)\;={𝔗}_{{Ȿ}_{𝔥}}\left(0\right)+\frac{1-\eta }{\mathrm{ABC}\left(\eta \right)}{𝕂}_{2}({ȶ}_{𝔫-1},\;{Ȿ}_{{𝔥}_{𝔫-1}},{𝔗}_{{Ȿ}_{{𝔥}_{𝔫-1}}},\;{\;𝔗}_{A_{{𝔥}_{𝔫-1}}},\\ {𝔎}_{{𝔥}_{𝔫-1}},\;{Ȿ}_{{𝔪}_{𝔫-1}},{𝔗}_{{𝔪}_{𝔫-1}})\;\\ +\frac{\eta }{\mathrm{ABC}\left(\eta \right)\lceil \eta }\int_{0}^{{ȶ}_{𝔫}}{𝕂}_{2}(ȶ,\;{Ȿ}_{𝔥}\left(ȶ\right),{𝔗}_{{Ȿ}_{𝔥}}\left(ȶ\right),\;{\;𝔗}_{A_{𝔥}}\left(ȶ\right),{𝔎}_{𝔥}\left(ȶ\right), \end{array}$$

(42) $$\begin{array}{l} {Ȿ}_{𝔪}\left(ȶ\right),{𝔗}_{𝔪}\left(ȶ\right)){\left({ȶ}_{𝔫}-t\right)}^{\eta -1}dȶ \end{array}$$

$$\begin{array}{l} {𝔗}_{A_{𝔥}}\left({ȶ}_{𝔫}\right)\;={\;𝔗}_{A_{𝔥}}\left(0\right)+\frac{1-\eta }{\mathrm{ABC}\left(\eta \right)}{𝕂}_{3}({ȶ}_{𝔫-1},\;{Ȿ}_{{𝔥}_{𝔫-1}},{𝔗}_{{Ȿ}_{{𝔥}_{𝔫-1}}},\\ {\;𝔗}_{A_{{𝔥}_{𝔫-1}}},{𝔎}_{{𝔥}_{𝔫-1}},\;{Ȿ}_{{𝔪}_{𝔫-1}},{𝔗}_{{𝔪}_{𝔫-1}})\\ +\frac{\eta }{\mathrm{ABC}\left(\eta \right)\lceil \eta }\int_{0}^{{ȶ}_{𝔫}}{𝕂}_{3}(ȶ,\;{Ȿ}_{𝔥}\left(ȶ0\right),{𝔗}_{{Ȿ}_{𝔥}}\left(ȶ\right),\;{\;𝔗}_{A_{𝔥}}\left(ȶ\right),{𝔎}_{𝔥}\left(ȶ\right), \end{array}$$

(43) $$\begin{array}{l} {Ȿ}_{𝔪}\left(ȶ\right),{𝔗}_{𝔪}\left(ȶ\right)){\left({ȶ}_{𝔫}-t\right)}^{\eta -1}dȶ \end{array}$$

$$\begin{array}{l} {𝔎}_{𝔥}\left({ȶ}_{𝔫}\right)\;=\;{𝔎}_{𝔥}\left(0\right)+\frac{1-\eta }{\mathrm{ABC}\left(\eta \right)}\\ {𝕂}_{4}\left({ȶ}_{𝔫-1},\;{Ȿ}_{{𝔥}_{𝔫-1}},{𝔗}_{{Ȿ}_{{𝔥}_{𝔫-1}}},\;{\;𝔗}_{A_{{𝔥}_{𝔫-1}}},{𝔎}_{{𝔥}_{𝔫-1}},{Ȿ}_{{𝔪}_{𝔫-1}},{𝔗}_{{𝔪}_{𝔫-1}}\right)\;\\ +\frac{\eta }{\mathrm{ABC}\left(\eta \right)\lceil \eta }\int_{0}^{{ȶ}_{𝔫}}{𝕂}_{4}(ȶ,\;{Ȿ}_{𝔥}\left(ȶ\right),{𝔗}_{{Ȿ}_{𝔥}}\left(ȶ\right),\;{\;𝔗}_{A_{𝔥}}\left(ȶ\right),{𝔎}_{𝔥}\left(ȶ\right), \end{array}$$

(44) $$\begin{array}{l} {Ȿ}_{𝔪}\left(ȶ\right),{𝔗}_{𝔪}\left(ȶ\right)){\left({ȶ}_{𝔫}-t\right)}^{\eta -1}dȶ\; \end{array}$$

$$\begin{array}{l} {Ȿ}_{𝔪}\left({ȶ}_{𝔫}\right)\;=\;{Ȿ}_{𝔪}\left(0\right)+\frac{1-\eta }{\mathrm{ABC}\left(\eta \right)}\\ {𝕂}_{5}\left({ȶ}_{𝔫-1},\;{Ȿ}_{{𝔥}_{𝔫-1}},{𝔗}_{{Ȿ}_{{𝔥}_{𝔫-1}}},\;{\;𝔗}_{A_{{𝔥}_{𝔫-1}}},{𝔎}_{{𝔥}_{𝔫-1}},\;{Ȿ}_{{𝔪}_{𝔫-1}},{𝔗}_{{𝔪}_{𝔫-1}}\right)\;\\ +\frac{\eta }{\mathrm{ABC}\left(\eta \right)\lceil \eta }\int_{0}^{{ȶ}_{𝔫}}{𝕂}_{5}(ȶ,\;{Ȿ}_{𝔥}\left(ȶ\right),{𝔗}_{{Ȿ}_{𝔥}}\left(ȶ\right),\;{\;𝔗}_{A_{𝔥}}\left(ȶ\right),{𝔎}_{𝔥}\left(ȶ\right), \end{array}$$

(45) $$\begin{array}{l} {Ȿ}_{𝔪}\left(ȶ\right),{𝔗}_{𝔪}\left(ȶ\right)){\left({ȶ}_{𝔫}-t\right)}^{\eta -1}dȶ\; \end{array}$$

$$\begin{array}{l} {𝔗}_{𝔪}\left({ȶ}_{𝔫}\right)\;={𝔗}_{𝔪}\left(0\right)+\frac{1-\eta }{\mathrm{ABC}\left(\eta \right)}\\ {𝕂}_{6}\left({ȶ}_{𝔫-1},\;{Ȿ}_{{𝔥}_{𝔫-1}},{𝔗}_{{Ȿ}_{{𝔥}_{𝔫-1}}},\;{\;𝔗}_{A_{{𝔥}_{𝔫-1}}},{𝔎}_{{𝔥}_{𝔫-1}},\;{Ȿ}_{{𝔪}_{𝔫-1}},{𝔗}_{{𝔪}_{𝔫-1}}\right)\;\\ +\frac{\eta }{\mathrm{ABC}\left(\eta \right)\lceil \eta }\int_{0}^{{ȶ}_{𝔫}}{𝕂}_{6}(ȶ,\;{Ȿ}_{𝔥}\left(ȶ\right),{𝔗}_{{Ȿ}_{𝔥}}\left(ȶ\right),\;{\;𝔗}_{A_{𝔥}}\left(ȶ\right),{𝔎}_{𝔥}\left(ȶ\right), \end{array}$$

(46) $$\begin{array}{l} {Ȿ}_{𝔪}\left(ȶ\right),{𝔗}_{𝔪}\left(ȶ\right)){\left({ȶ}_{𝔫}-t\right)}^{\eta -1}dȶ\; \end{array}$$

By subtracting Ȿ_𝔥_(ȶ_𝔫_) from Ȿ_𝔥_(ȶ_𝔫+1_), 𝔗_Ȿ_𝔥__(ȶ_𝔫_) from 𝔗_Ȿ_𝔥__(ȶ_𝔫+1_), ${\;𝔗}_{A_{𝔥}}\left({ȶ}_{𝔫}\right)$ from ${\;𝔗}_{A_{𝔥}}\left({ȶ}_{𝔫+1}\right)$, 𝔎_𝔥_(ȶ_𝔫_) from 𝔎_𝔥_(ȶ_𝔫+1_), Ȿ_𝔪_(ȶ_𝔫_) from *S*_𝔪_(ȶ_𝔫+1_) and 𝔗_𝔪_(ȶ_𝔫_) from 𝔗_𝔪_(ȶ_𝔫+1_), we get the following


$$\begin{array}{l} {Ȿ}_{𝔥}\left({ȶ}_{𝔫+1}\right)=S_{𝔥}\left({ȶ}_{𝔫}\right)+\frac{1-\eta }{\mathrm{ABC}\left(\eta \right)}\\ \left[\begin{array}{c} {𝕂}_{1}\left({ȶ}_{𝔫},\;{Ȿ}_{{𝔥}_{𝔫}},{𝔗}_{{Ȿ}_{{𝔥}_{𝔫}}},\;{\;𝔗}_{A_{{𝔥}_{𝔫}}},{𝔎}_{{𝔥}_{𝔫}},\;{Ȿ}_{{𝔪}_{𝔫}},{𝔗}_{{𝔪}_{𝔫}}\right)-\\ {𝕂}_{1}\left({ȶ}_{𝔫-1},\;{Ȿ}_{{𝔥}_{𝔫-1}},{𝔗}_{{Ȿ}_{{𝔥}_{𝔫-1}}},\;{\;𝔗}_{A_{{𝔥}_{𝔫-1}}},{𝔎}_{{𝔥}_{𝔫-1}},\;S_{{𝔪}_{𝔫-1}},{𝔗}_{{𝔪}_{𝔫-1}}\right) \end{array}\right]+\;\\ \frac{\eta }{\mathrm{ABC}\left(\eta \right)\lceil \eta }\int_{0}^{{ȶ}_{𝔫+1}}{𝕂}_{1}(ȶ,\;{Ȿ}_{𝔥}\left(ȶ\right),{𝔗}_{{Ȿ}_{𝔥}}\left(ȶ\right),\;{\;𝔗}_{A_{𝔥}}\left(ȶ\right),\\ {𝔎}_{𝔥}\left(ȶ\right),\;{Ȿ}_{𝔪}\left(ȶ\right),{𝔗}_{𝔪}\left(ȶ\right)){\left({ȶ}_{𝔫+1}-t\right)}^{\eta -1}dȶ-\;\\ \frac{\eta }{\mathrm{ABC}\left(\eta \right)\lceil \eta }\int_{0}^{{ȶ}_{𝔫}}{𝕂}_{1}(ȶ,\;{Ȿ}_{𝔥}\left(ȶ\right),{𝔗}_{{Ȿ}_{𝔥}}\left(ȶ\right),\;{\;𝔗}_{A_{𝔥}}\left(ȶ\right),{𝔎}_{𝔥}\left(ȶ\right), \end{array}$$

(47) $$\begin{array}{l} {Ȿ}_{𝔪}\left(ȶ\right),{𝔗}_{𝔪}\left(ȶ\right)){\left({ȶ}_{𝔫}-t\right)}^{\eta -1}dȶ \end{array}$$

$$\begin{array}{l} {𝔗}_{{Ȿ}_{𝔥}}\left({ȶ}_{𝔫+1}\right)={𝔗}_{{Ȿ}_{𝔥}}\left({ȶ}_{𝔫}\right)+\frac{1-\eta }{\mathrm{ABC}\left(\eta \right)}\\ \left[\begin{array}{c} {𝕂}_{2}\left({ȶ}_{𝔫},\;{Ȿ}_{{𝔥}_{𝔫}},{𝔗}_{{Ȿ}_{{𝔥}_{𝔫}}},\;{\;𝔗}_{A_{{𝔥}_{𝔫}}},{𝔎}_{{𝔥}_{𝔫}},\;{Ȿ}_{{𝔪}_{𝔫}},{𝔗}_{{𝔪}_{𝔫}}\right)-\\ {𝕂}_{2}\left({ȶ}_{𝔫-1},\;{Ȿ}_{{𝔥}_{𝔫-1}},{𝔗}_{{Ȿ}_{{𝔥}_{𝔫-1}}},\;{\;𝔗}_{A_{{𝔥}_{𝔫-1}}},{𝔎}_{{𝔥}_{𝔫-1}},\;S_{{𝔪}_{𝔫-1}},{𝔗}_{{𝔪}_{𝔫-1}}\right) \end{array}\right]\;\\ +\frac{\eta }{\mathrm{ABC}\left(\eta \right)\lceil \eta }\int_{0}^{{ȶ}_{𝔫+1}}{𝕂}_{2}(ȶ,\;{Ȿ}_{𝔥}\left(ȶ\right),{𝔗}_{{Ȿ}_{𝔥}}\left(ȶ\right),\;{\;𝔗}_{A_{𝔥}}\left(ȶ\right),{𝔎}_{𝔥}\left(ȶ\right),\\ {Ȿ}_{𝔪}\left(ȶ\right),{𝔗}_{𝔪}\left(ȶ\right)){\left({ȶ}_{𝔫+1}-t\right)}^{\eta -1}dȶ-\;\\ \frac{\eta }{\mathrm{ABC}\left(\eta \right)\lceil \eta }\int_{0}^{{ȶ}_{𝔫}}{𝕂}_{2}(ȶ,\;{Ȿ}_{𝔥}\left(ȶ\right),{𝔗}_{{Ȿ}_{𝔥}}\left(ȶ\right),\;{\;𝔗}_{A_{𝔥}}\left(ȶ\right),{𝔎}_{𝔥}\left(ȶ\right), \end{array}$$

(48) $$\begin{array}{l} {Ȿ}_{𝔪}\left(ȶ\right),{𝔗}_{𝔪}\left(ȶ\right)){\left({ȶ}_{𝔫}-t\right)}^{\eta -1}dȶ \end{array}$$

$$\begin{array}{l} {𝔗}_{A_{𝔥}}\left({ȶ}_{𝔫+1}\right)={\;𝔗}_{A_{𝔥}}\left({ȶ}_{𝔫}\right)+\frac{1-\eta }{\mathrm{ABC}\left(\eta \right)}\\ \left[\begin{array}{c} {𝕂}_{3}\left({ȶ}_{𝔫},\;{Ȿ}_{{𝔥}_{𝔫}},{𝔗}_{{Ȿ}_{{𝔥}_{𝔫}}},\;{\;𝔗}_{A_{{𝔥}_{𝔫}}},{𝔎}_{{𝔥}_{𝔫}},\;{Ȿ}_{{𝔪}_{𝔫}},{𝔗}_{{𝔪}_{𝔫}}\right)-\\ {𝕂}_{3}\left({ȶ}_{𝔫-1},\;{Ȿ}_{{𝔥}_{𝔫-1}},{𝔗}_{{Ȿ}_{{𝔥}_{𝔫-1}}},\;{\;𝔗}_{A_{{𝔥}_{𝔫-1}}},{𝔎}_{{𝔥}_{𝔫-1}},\;S_{{𝔪}_{𝔫-1}},{𝔗}_{{𝔪}_{𝔫-1}}\right) \end{array}\right]+\;\\ \frac{\eta }{\mathrm{ABC}\left(\eta \right)\lceil \eta }\int_{0}^{{ȶ}_{𝔫+1}}{𝕂}_{3}(ȶ,\;{Ȿ}_{𝔥}\left(ȶ\right),{𝔗}_{{Ȿ}_{𝔥}}\left(ȶ\right),\;{\;𝔗}_{A_{𝔥}}\left(ȶ\right),{𝔎}_{𝔥}\left(ȶ\right),\\ {Ȿ}_{𝔪}\left(ȶ\right),{𝔗}_{𝔪}\left(ȶ\right)){\left({ȶ}_{𝔫+1}-t\right)}^{\eta -1}dȶ-\;\\ \frac{\eta }{\mathrm{ABC}\left(\eta \right)\lceil \eta }\int_{0}^{{ȶ}_{𝔫}}{𝕂}_{3}(ȶ,\;{Ȿ}_{𝔥}\left(ȶ\right),{𝔗}_{{Ȿ}_{𝔥}}\left(ȶ\right),\;{\;𝔗}_{A_{𝔥}}\left(ȶ\right),{𝔎}_{𝔥}\left(ȶ\right), \end{array}$$

(49) $$\begin{array}{l} {Ȿ}_{𝔪}\left(ȶ\right),{𝔗}_{𝔪}\left(ȶ\right)){\left({ȶ}_{𝔫}-t\right)}^{\eta -1}dȶ \end{array}$$

$$\begin{array}{l} {𝔎}_{𝔥}\left({ȶ}_{𝔫+1}\right)={𝔎}_{𝔥}\left({ȶ}_{𝔫}\right)+\frac{1-\eta }{\mathrm{ABC}\left(\eta \right)}\\ \left[\begin{array}{c} {𝕂}_{4}\left({ȶ}_{𝔫},\;{Ȿ}_{{𝔥}_{𝔫}},{𝔗}_{{Ȿ}_{{𝔥}_{𝔫}}},\;{\;𝔗}_{A_{{𝔥}_{𝔫}}},{𝔎}_{{𝔥}_{𝔫}},\;{Ȿ}_{{𝔪}_{𝔫}},{𝔗}_{{𝔪}_{𝔫}}\right)\\ -{𝕂}_{4}\left({ȶ}_{𝔫-1},\;{Ȿ}_{{𝔥}_{𝔫-1}},{𝔗}_{{Ȿ}_{{𝔥}_{𝔫-1}}},\;{\;𝔗}_{A_{{𝔥}_{𝔫-1}}},{𝔎}_{{𝔥}_{𝔫-1}},\;S_{{𝔪}_{𝔫-1}},{𝔗}_{{𝔪}_{𝔫-1}}\right) \end{array}\right]\;\\ +\frac{\eta }{\mathrm{ABC}\left(\eta \right)\lceil \eta }\int_{0}^{{ȶ}_{𝔫+1}}{𝕂}_{4}\left(ȶ,\;{Ȿ}_{𝔥}(ȶ\right),{𝔗}_{{Ȿ}_{𝔥}}\left(ȶ\right),\;{\;𝔗}_{A_{𝔥}}\left(ȶ\right),{𝔎}_{𝔥}\left(ȶ\right),\\ {Ȿ}_{𝔪}\left(ȶ\right),{𝔗}_{𝔪}\left(ȶ\right)){\left({ȶ}_{𝔫+1}-t\right)}^{\eta -1}dȶ-\;\\ +\frac{\eta }{\mathrm{ABC}\left(\eta \right)\lceil \eta }\int_{0}^{{ȶ}_{𝔫}}{𝕂}_{4}(ȶ,\;{Ȿ}_{𝔥}\left(ȶ\right),{𝔗}_{{Ȿ}_{𝔥}}\left(ȶ\right),\;{\;𝔗}_{A_{𝔥}}\left(ȶ\right),{𝔎}_{𝔥}\left(ȶ\right), \end{array}$$

(50) $$\begin{array}{l} {Ȿ}_{𝔪}\left(ȶ\right),{𝔗}_{𝔪}\left(ȶ\right)){\left({ȶ}_{𝔫}-t\right)}^{\eta -1}dȶ \end{array}$$

$$\begin{array}{l} {Ȿ}_{𝔪}\left({ȶ}_{𝔫+1}\right)=S_{𝔪}\left({ȶ}_{𝔫}\right)+\frac{1-\eta }{\mathrm{ABC}\left(\eta \right)}\\ \left[\begin{array}{c} {𝕂}_{5}\left({ȶ}_{𝔫},\;{Ȿ}_{{𝔥}_{𝔫}},{𝔗}_{{Ȿ}_{{𝔥}_{𝔫}}},\;{\;𝔗}_{A_{{𝔥}_{𝔫}}},{𝔎}_{{𝔥}_{𝔫}},\;{Ȿ}_{{𝔪}_{𝔫}},{𝔗}_{{𝔪}_{𝔫}}\right)\\ -{𝕂}_{5}\left({ȶ}_{𝔫-1},\;{Ȿ}_{{𝔥}_{𝔫-1}},{𝔗}_{{Ȿ}_{{𝔥}_{𝔫-1}}},\;{\;𝔗}_{A_{{𝔥}_{𝔫-1}}},{𝔎}_{{𝔥}_{𝔫-1}},\;S_{{𝔪}_{𝔫-1}},{𝔗}_{{𝔪}_{𝔫-1}}\right) \end{array}\right]+\;\\ \frac{\eta }{\mathrm{ABC}\left(\eta \right)\lceil \eta }\int_{0}^{{ȶ}_{𝔫+1}}{𝕂}_{6}(ȶ,\;{Ȿ}_{𝔥}\left(ȶ\right),{𝔗}_{{Ȿ}_{𝔥}}\left(ȶ\right),\;{\;𝔗}_{A_{𝔥}}\left(ȶ\right),{𝔎}_{𝔥}\left(ȶ\right),\\ {Ȿ}_{𝔪}\left(ȶ\right),{𝔗}_{𝔪}\left(ȶ\right)){\left({ȶ}_{𝔫+1}-t\right)}^{\eta -1}dȶ-\;\\ +\frac{\eta }{\mathrm{ABC}\left(\eta \right)\lceil \eta }\int_{0}^{{ȶ}_{𝔫}}{𝕂}_{6}(ȶ,\;{Ȿ}_{𝔥}\left(ȶ\right),{𝔗}_{{Ȿ}_{𝔥}}\left(ȶ\right),\;{\;𝔗}_{A_{𝔥}}\left(ȶ\right),{𝔎}_{𝔥}\left(ȶ\right), \end{array}$$

(51) $$\begin{array}{l} {Ȿ}_{𝔪}\left(ȶ\right),{𝔗}_{𝔪}\left(ȶ\right)){\left({ȶ}_{𝔫}-t\right)}^{\eta -1}dȶ \end{array}$$

$$\begin{array}{l} {𝔗}_{𝔪}\left({ȶ}_{𝔫+1}\right)={𝔗}_{𝔪}\left({ȶ}_{𝔫}\right)+\frac{1-\eta }{\mathrm{ABC}\left(\eta \right)}\\ \left[\begin{array}{c} {𝕂}_{6}\left({ȶ}_{𝔫},\;{Ȿ}_{{𝔥}_{𝔫}},{𝔗}_{{Ȿ}_{{𝔥}_{𝔫}}},\;{\;𝔗}_{A_{{𝔥}_{𝔫}}},{𝔎}_{{𝔥}_{𝔫}},\;{Ȿ}_{{𝔪}_{𝔫}},{𝔗}_{{𝔪}_{𝔫}}\right)-\\ {𝕂}_{6}\left({ȶ}_{𝔫-1},\;{Ȿ}_{{𝔥}_{𝔫-1}},{𝔗}_{{Ȿ}_{{𝔥}_{𝔫-1}}},\;{\;𝔗}_{A_{{𝔥}_{𝔫-1}}},{𝔎}_{{𝔥}_{𝔫-1}},\;S_{{𝔪}_{𝔫-1}},{𝔗}_{{𝔪}_{𝔫-1}}\right) \end{array}\right]+\;\\ \frac{\eta }{\mathrm{ABC}\left(\eta \right)\lceil \eta }\int_{0}^{{ȶ}_{𝔫+1}}{𝕂}_{6}(ȶ,\;{Ȿ}_{𝔥}\left(ȶ\right),{𝔗}_{{Ȿ}_{𝔥}}\left(ȶ\right),\;{\;𝔗}_{A_{𝔥}}\left(ȶ\right),{𝔎}_{𝔥}\left(ȶ\right),\\ {Ȿ}_{𝔪}\left(ȶ\right),{𝔗}_{𝔪}\left(ȶ\right)){\left({ȶ}_{𝔫+1}-t\right)}^{\eta -1}dȶ-\;\\ +\frac{\eta }{\mathrm{ABC}\left(\eta \right)\lceil \eta }\int_{0}^{{ȶ}_{𝔫}}{𝕂}_{6}(ȶ,\;{Ȿ}_{𝔥}\left(ȶ\right),{𝔗}_{{Ȿ}_{𝔥}}\left(ȶ\right),\;{\;𝔗}_{A_{𝔥}}\left(ȶ\right),{𝔎}_{𝔥}\left(ȶ\right), \end{array}$$

(52) $$\begin{array}{l} {Ȿ}_{𝔪}\left(ȶ\right),{𝔗}_{𝔪}\left(ȶ\right)){\left({ȶ}_{𝔫}-t\right)}^{\eta -1}dȶ \end{array}$$

The Equations 47–52 become


$$\begin{array}{l} {Ȿ}_{𝔥}\left({ȶ}_{𝔫+1}\right)=S_{𝔥}\left({ȶ}_{𝔫}\right)+\frac{1-\eta }{\mathrm{ABC}\left(\eta \right)}\\ \left[\begin{array}{c} {𝕂}_{1}({ȶ}_{𝔫},\;{Ȿ}_{{𝔥}_{𝔫}},{𝔗}_{{Ȿ}_{{𝔥}_{𝔫}}},\;{\;𝔗}_{A_{{𝔥}_{𝔫}}},{𝔎}_{{𝔥}_{𝔫}},\\ {Ȿ}_{{𝔪}_{𝔫}},{𝔗}_{{𝔪}_{𝔫}}(-\\ {𝕂}_{1}\left({ȶ}_{𝔫-1},\;{Ȿ}_{{𝔥}_{𝔫-1}},{𝔗}_{{Ȿ}_{{𝔥}_{𝔫-1}}},\;{\;𝔗}_{A_{{𝔥}_{𝔫-1}}},{𝔎}_{{𝔥}_{𝔫-1}},\;S_{{𝔪}_{𝔫-1}},{𝔗}_{{𝔪}_{𝔫-1}}\right) \end{array}\right] \end{array}$$

(53) $$\begin{array}{l} +{𝔸}_{\eta ,1}^{1}-{𝔸}_{\eta ,2}^{1} \end{array}$$

$$\begin{array}{l} {𝔗}_{{Ȿ}_{𝔥}}\left({ȶ}_{𝔫+1}\right)={𝔗}_{{Ȿ}_{𝔥}}\left({ȶ}_{𝔫}\right)+\frac{1-\eta }{\mathrm{ABC}\left(\eta \right)}\\ \left[\begin{array}{c} {𝕂}_{2}({ȶ}_{𝔫},\;{Ȿ}_{{𝔥}_{𝔫}},{𝔗}_{{Ȿ}_{{𝔥}_{𝔫}}},\;{\;𝔗}_{A_{{𝔥}_{𝔫}}},{𝔎}_{{𝔥}_{𝔫}},\\ {Ȿ}_{{𝔪}_{𝔫}},{𝔗}_{{𝔪}_{𝔫}}(-\\ {𝕂}_{2}({ȶ}_{𝔫-1},\;{Ȿ}_{{𝔥}_{𝔫-1}},{𝔗}_{{Ȿ}_{{𝔥}_{𝔫-1}}},\;{\;𝔗}_{A_{{𝔥}_{𝔫-1}}},{𝔎}_{{𝔥}_{𝔫-1}},\\ {Ȿ}_{{𝔪}_{𝔫-1}},{𝔗}_{{𝔪}_{𝔫-1}}( \end{array}\right] \end{array}$$

(54) $$\begin{array}{l} +{𝔸}_{\eta ,1}^{2}-{𝔸}_{\eta ,2}^{2} \end{array}$$

$$\begin{array}{l} {𝔗}_{A_{𝔥}}\left({ȶ}_{𝔫+1}\right)+{𝔗}_{A_{𝔥}}\left({ȶ}_{𝔫}\right)+\frac{1-\eta }{\mathrm{ABC}\left(\eta \right)}\\ \left[\begin{array}{c} {𝕂}_{3}\left({ȶ}_{𝔫},{Ȿ}_{{𝔥}_{𝔫}},{𝔗}_{{Ȿ}_{{𝔥}_{𝔫}}},\;{\;𝔗}_{A_{{𝔥}_{𝔫}}},{𝔎}_{{𝔥}_{𝔫}},\;{Ȿ}_{{𝔪}_{𝔫}},{𝔗}_{{𝔪}_{𝔫}}\right)-\\ {𝕂}_{3}\left({ȶ}_{𝔫-1},\;{Ȿ}_{{𝔥}_{𝔫-1}},{𝔗}_{{Ȿ}_{{𝔥}_{𝔫-1}}},\;{\;𝔗}_{A_{{𝔥}_{𝔫-1}}},{𝔎}_{{𝔥}_{𝔫-1}},\;S_{{𝔪}_{𝔫-1}},{𝔗}_{{𝔪}_{𝔫-1}}\right) \end{array}\right] \end{array}$$

(55) $$\begin{array}{l} +{𝔸}_{\eta ,1}^{3}-\;{𝔸}_{\eta ,2}^{3} \end{array}$$

$$\begin{array}{l} {𝔎}_{𝔥}\left({ȶ}_{𝔫+1}\right)={𝔎}_{𝔥}\left({ȶ}_{𝔫}\right)+\frac{1-\eta }{\mathrm{ABC}\left(\eta \right)}\\ \left[\begin{array}{c} {𝕂}_{4}\left({ȶ}_{𝔫},\;{Ȿ}_{{𝔥}_{𝔫}},{𝔗}_{{Ȿ}_{{𝔥}_{𝔫}}},\;{\;𝔗}_{A_{{𝔥}_{𝔫}}},{𝔎}_{{𝔥}_{𝔫}},\;{Ȿ}_{{𝔪}_{𝔫}},{𝔗}_{{𝔪}_{𝔫}}\right)-\\ {𝕂}_{4}\left({ȶ}_{𝔫-1},\;{Ȿ}_{{𝔥}_{𝔫-1}},{𝔗}_{{Ȿ}_{{𝔥}_{𝔫-1}}},\;{\;𝔗}_{A_{{𝔥}_{𝔫-1}}},{𝔎}_{{𝔥}_{𝔫-1}},\;S_{{𝔪}_{𝔫-1}},{𝔗}_{{𝔪}_{𝔫-1}}\right) \end{array}\right] \end{array}$$

(56) $$\begin{array}{l} +{𝔸}_{\eta ,1}^{4}-{𝔸}_{\eta ,2}^{4}\; \end{array}$$

$$\begin{array}{l} {Ȿ}_{𝔪}\left({ȶ}_{𝔫+1}\right)=S_{𝔪}\left({ȶ}_{𝔫}\right)+\frac{1-\eta }{\mathrm{ABC}\left(\eta \right)}\\ \left[\begin{array}{c} {𝕂}_{5}\left({ȶ}_{𝔫},\;{Ȿ}_{{𝔥}_{𝔫}},{𝔗}_{{Ȿ}_{{𝔥}_{𝔫}}},\;{\;𝔗}_{A_{{𝔥}_{𝔫}}},{𝔎}_{{𝔥}_{𝔫}},\;{Ȿ}_{{𝔪}_{𝔫}},{𝔗}_{{𝔪}_{𝔫}}\right)-\\ {𝕂}_{5}\left({ȶ}_{𝔫-1},\;{Ȿ}_{{𝔥}_{𝔫-1}},{𝔗}_{{Ȿ}_{{𝔥}_{𝔫-1}}},\;{\;𝔗}_{A_{{𝔥}_{𝔫-1}}},{𝔎}_{{𝔥}_{𝔫-1}},\;S_{{𝔪}_{𝔫-1}},{𝔗}_{{𝔪}_{𝔫-1}}\right) \end{array}\right] \end{array}$$

(57) $$\begin{array}{l} +{𝔸}_{\eta ,1}^{5}-{𝔸}_{\eta ,2}^{5}\; \end{array}$$

$$\begin{array}{l} {𝔗}_{𝔪}\left({ȶ}_{𝔫+1}\right)={𝔗}_{𝔪}\left({ȶ}_{𝔫}\right)+\frac{1-\eta }{\mathrm{ABC}\left(\eta \right)}\\ \left[\begin{array}{c} {𝕂}_{6}\left({ȶ}_{𝔫},\;{Ȿ}_{{𝔥}_{𝔫}},{𝔗}_{{Ȿ}_{{𝔥}_{𝔫}}},\;{\;𝔗}_{A_{{𝔥}_{𝔫}}},{𝔎}_{{𝔥}_{𝔫}},\;{Ȿ}_{{𝔪}_{𝔫}},{𝔗}_{{𝔪}_{𝔫}}\right)-\\ {𝕂}_{6}\left({ȶ}_{𝔫-1},\;{Ȿ}_{{𝔥}_{𝔫-1}},{𝔗}_{{Ȿ}_{{𝔥}_{𝔫-1}}},\;{\;𝔗}_{A_{{𝔥}_{𝔫-1}}},{𝔎}_{{𝔥}_{𝔫-1}},\;S_{{𝔪}_{𝔫-1}},{𝔗}_{{𝔪}_{𝔫-1}}\right) \end{array}\right] \end{array}$$

(58) $$\begin{array}{l} +{𝔸}_{\eta ,1}^{6}-\;{𝔸}_{\eta ,2}^{6} \end{array}$$

Where


$$\begin{array}{l} {𝔸}_{\eta ,1}^{1}=\frac{\eta }{\mathrm{ABC}\left(\eta \right)\lceil \eta }\int_{0}^{{ȶ}_{𝔫+1}}{𝕂}_{1}(ȶ,\;{Ȿ}_{𝔥}\left(ȶ\right),{𝔗}_{{Ȿ}_{𝔥}}\left(ȶ\right),\;{\;𝔗}_{A_{𝔥}}\left(ȶ\right), \end{array}$$

(59) $$\begin{array}{l} {𝔎}_{𝔥}\left(ȶ\right),\;{Ȿ}_{𝔪}\left(ȶ\right),{𝔗}_{𝔪}\left(ȶ\right)){\left({ȶ}_{𝔫+1}-t\right)}^{\eta -1}dȶ \end{array}$$

$$\begin{array}{l} {𝔸}_{\eta ,1}^{2}=\frac{\eta }{\mathrm{ABC}\left(\eta \right)\lceil \eta }\int_{\;0}^{{ȶ}_{𝔫+1}}{𝕂}_{2}(ȶ,\;{Ȿ}_{𝔥}\left(ȶ\right),{𝔗}_{{Ȿ}_{𝔥}}\left(ȶ\right),\;{\;𝔗}_{A_{𝔥}}\left(ȶ\right), \end{array}$$

(60) $$\begin{array}{l} {𝔎}_{𝔥}\left(ȶ\right),\;{Ȿ}_{𝔪}\left(ȶ),{𝔗}_{𝔪}\left(ȶ\right)\right){\left({ȶ}_{𝔫+1}-t\right)}^{\eta -1}dȶ \end{array}$$

$$\begin{array}{l} {𝔸}_{\eta ,1}^{3}=\frac{\eta }{\mathrm{ABC}\left(\eta \right)\lceil \eta }\int_{0}^{{ȶ}_{𝔫+1}}{𝕂}_{3}(ȶ,\;{Ȿ}_{𝔥}\left(ȶ\right),{𝔗}_{{Ȿ}_{𝔥}}\left(ȶ\right),\;{\;𝔗}_{A_{𝔥}}\left(ȶ\right), \end{array}$$

(61) $$\begin{array}{l} {𝔎}_{𝔥}\left(ȶ\right),\;{Ȿ}_{𝔪}\left(ȶ\right),{𝔗}_{𝔪}\left(ȶ\right)){\left({ȶ}_{𝔫+1}-t\right)}^{\eta -1}dȶ\; \end{array}$$

$$\begin{array}{l} {𝔸}_{\eta ,1}^{4}=\frac{\eta }{\mathrm{ABC}\left(\eta \right)\lceil \eta }\int_{0}^{{ȶ}_{𝔫+1}}{𝕂}_{4}(ȶ,\;{Ȿ}_{𝔥}\left(ȶ\right),{𝔗}_{{Ȿ}_{𝔥}}\left(ȶ\right),\;{\;𝔗}_{A_{𝔥}}\left(ȶ\right), \end{array}$$

(62) $$\begin{array}{l} {𝔎}_{𝔥}\left(ȶ\right),\;{Ȿ}_{𝔪}\left(ȶ\right),{𝔗}_{𝔪}\left(ȶ\right)){\left({ȶ}_{𝔫+1}-t\right)}^{\eta -1}dȶ\; \end{array}$$

$$\begin{array}{l} {𝔸}_{\eta ,1}^{5}=\frac{\eta }{\mathrm{ABC}\left(\eta \right)\lceil \eta }\int_{0}^{{ȶ}_{𝔫+1}}{𝕂}_{5}(ȶ,\;{Ȿ}_{𝔥}\left(ȶ\right),{𝔗}_{{Ȿ}_{𝔥}}\left(ȶ\right),\;{\;𝔗}_{A_{𝔥}}\left(ȶ\right), \end{array}$$

(63) $$\begin{array}{l} {𝔎}_{𝔥}\left(ȶ\right),\;{Ȿ}_{𝔪}\left(ȶ\right),{𝔗}_{𝔪}\left(ȶ\right)){\left({ȶ}_{𝔫+1}-t\right)}^{\eta -1}dȶ \end{array}$$

$$\begin{array}{l} {𝔸}_{\eta ,1}^{6}=\frac{\eta }{\mathrm{ABC}\left(\eta \right)\lceil \eta }\int_{0}^{{ȶ}_{𝔫+1}}{𝕂}_{6}(ȶ,\;{Ȿ}_{𝔥}\left(ȶ\right),{𝔗}_{{Ȿ}_{𝔥}}\left(ȶ\right),\;{\;𝔗}_{A_{𝔥}}\left(ȶ\right), \end{array}$$

(64) $$\begin{array}{l} {𝔎}_{𝔥}\left(ȶ\right),\;{Ȿ}_{𝔪}\left(ȶ\right),{𝔗}_{𝔪}\left(ȶ\right)){\left({ȶ}_{𝔫+1}-t\right)}^{\eta -1}dȶ\; \end{array}$$

and


$$\begin{array}{l} {𝔸}_{\eta ,2}^{1}=\frac{\eta }{\mathrm{ABC}\left(\eta \right)\lceil \eta }\int_{0}^{{ȶ}_{𝔫}}{𝕂}_{1}(ȶ,\;{Ȿ}_{𝔥}\left(ȶ\right),{𝔗}_{{Ȿ}_{𝔥}}\left(ȶ\right),\;{\;𝔗}_{A_{𝔥}}\left(ȶ\right), \end{array}$$

(65) $$\begin{array}{l} {𝔎}_{𝔥}\left(ȶ\right),\;{Ȿ}_{𝔪}\left(ȶ\right),{𝔗}_{𝔪}\left(ȶ\right)){\left({ȶ}_{𝔫}-t\right)}^{\eta -1}dȶ\; \end{array}$$

$$\begin{array}{l} {𝔸}_{\eta ,2}^{2}=\frac{\eta }{\mathrm{ABC}\left(\eta \right)\lceil \eta }\int_{0}^{{ȶ}_{𝔫}}{𝕂}_{2}(ȶ,\;{Ȿ}_{𝔥}\left(ȶ\right),{𝔗}_{{Ȿ}_{𝔥}}\left(ȶ\right),\;{\;𝔗}_{A_{𝔥}}\left(ȶ\right), \end{array}$$

(66) $$\begin{array}{l} {𝔎}_{𝔥}\left(ȶ\right),\;{Ȿ}_{𝔪}\left(ȶ\right),{𝔗}_{𝔪}\left(ȶ\right)){\left({ȶ}_{𝔫}-t\right)}^{\eta -1}dȶ\; \end{array}$$

$$\begin{array}{l} {𝔸}_{\eta ,2}^{3}=\frac{\eta }{\mathrm{ABC}\left(\eta \right)\lceil \eta }\int_{0}^{{ȶ}_{𝔫}}{𝕂}_{3}(ȶ,\;{Ȿ}_{𝔥}\left(ȶ\right),{𝔗}_{{Ȿ}_{𝔥}}\left(ȶ\right),\;{\;𝔗}_{A_{𝔥}}\left(ȶ\right), \end{array}$$

(67) $$\begin{array}{l} {𝔎}_{𝔥}\left(ȶ\right),\;{Ȿ}_{𝔪}\left(ȶ\right),{𝔗}_{𝔪}\left(ȶ\right)){\left({ȶ}_{𝔫}-t\right)}^{\eta -1}dȶ\; \end{array}$$

$$\begin{array}{l} {𝔸}_{\eta ,2}^{4}=\frac{\eta }{\mathrm{ABC}\left(\eta \right)\lceil \eta }\int_{0}^{{ȶ}_{𝔫}}{𝕂}_{4}(ȶ,\;{Ȿ}_{𝔥}\left(ȶ\right),{𝔗}_{{Ȿ}_{𝔥}}\left(ȶ\right),\;{\;𝔗}_{A_{𝔥}}\left(ȶ𝕃\right), \end{array}$$

(68) $$\begin{array}{l} {𝔎}_{𝔥}\left(ȶ\right),\;{Ȿ}_{𝔪}\left(ȶ\right),{𝔗}_{𝔪}\left(ȶ\right)){\left({ȶ}_{𝔫}-t\right)}^{\eta -1}d\; \end{array}$$

$$\begin{array}{l} {𝔸}_{\eta ,2}^{5}=\frac{\eta }{\mathrm{ABC}\left(\eta \right)\lceil \eta }\int_{0}^{{ȶ}_{𝔫}}{𝕂}_{5}(ȶ,\;{Ȿ}_{𝔥}\left(ȶ\right),{𝔗}_{{Ȿ}_{𝔥}}\left(ȶ\right),\;{\;𝔗}_{A_{𝔥}}\left(ȶ\right), \end{array}$$

(69) $$\begin{array}{l} {𝔎}_{𝔥}\left(ȶ\right),\;{Ȿ}_{𝔪}\left(ȶ\right),{𝔗}_{𝔪}\left(ȶ\right)){\left({ȶ}_{𝔫}-t\right)}^{\eta -1}dȶ\; \end{array}$$

$$\begin{array}{l} {𝔸}_{\eta ,2}^{6}=\frac{\eta }{\mathrm{ABC}\left(\eta \right)\lceil \eta }\int_{0}^{{ȶ}_{𝔫}}{𝕂}_{6}(ȶ,\;{Ȿ}_{𝔥}\left(ȶ\right),{𝔗}_{{Ȿ}_{𝔥}}\left(ȶ\right),\;{\;𝔗}_{A_{𝔥}}\left(ȶ\right), \end{array}$$

(70) $$\begin{array}{l} {𝔎}_{𝔥}\left(ȶ\right),\;{Ȿ}_{𝔪}\left(ȶ\right),{𝔗}_{𝔪}\left(ȶ\right)){\left({ȶ}_{𝔫}-t\right)}^{\eta -1}dȶ\; \end{array}$$

Now, approximating ${𝔸}_{\eta ,1}^{1}\;,\;{𝔸}_{\eta ,1}^{2},\;{𝔸}_{\eta ,1}^{3},\;{𝔸}_{\eta ,1}^{4},\;{𝔸}_{\eta ,1}^{5},\;{𝔸}_{\eta ,1}^{6}$ and ${𝔸}_{\eta ,2}^{1}\;,\;{𝔸}_{\eta ,2}^{2},\;{𝔸}_{\eta ,2}^{3},\;{𝔸}_{\eta ,2}^{4},\;{𝔸}_{\eta ,2}^{5},\;{𝔸}_{\eta ,2}^{6}$ with the help of Lagrange's polynomials


$$\begin{array}{l} \mathbb{P}\left(ȶ\right)\approx \frac{ȶ\;-\;t_{𝔫-1}}{{ȶ}_{𝔫}-\;t_{𝔫-1}}f\left({ȶ}_{𝔫},y_{𝔫}\right)+\frac{ȶ\;-\;t_{𝔫}}{{ȶ}_{𝔫-1}\;-\;t_{𝔫}}f\left({ȶ}_{𝔫-1},y_{𝔫-1}\right),\; \end{array}$$

(71) $$\begin{array}{l} =\frac{ȶ\;-t_{𝔫-1}}{𝔥}f\left({ȶ}_{𝔫},y_{𝔫-1}\right)+\frac{ȶ\;-\;t_{𝔫}}{𝔥}f\left({ȶ}_{𝔫-1},y_{𝔫-1}\right)\; \end{array}$$

Now, only consider the Equation 59 to evaluate under the Equation 71, that is given as


$$\begin{array}{l} {𝔸}_{\eta ,1}^{1}=\frac{\eta }{\mathrm{ABC}\left(\eta \right)\lceil \eta }\int_{0}^{{ȶ}_{𝔫+1}}{\left({ȶ}_{𝔫+1}-t\right)}^{\eta -1}\\ \left[\begin{array}{c} \frac{ȶ-t_{𝔫-1}}{𝔥}{𝕂}_{1}\left({ȶ}_{𝔫},\;{Ȿ}_{{𝔥}_{𝔫}},{𝔗}_{{Ȿ}_{{𝔥}_{𝔫}}},\;{\;𝔗}_{A_{{𝔥}_{𝔫}}},{𝔎}_{{𝔥}_{𝔫}},\;{Ȿ}_{{𝔪}_{𝔫}},{𝔗}_{{𝔪}_{𝔫}}\right)+\\ \frac{ȶ-t_{𝔫}}{𝔥}{𝕂}_{1}\left({ȶ}_{𝔫-1},\;{Ȿ}_{{𝔥}_{𝔫-1}},{𝔗}_{S_{{𝔥}_{𝔫-1}}},\;{\;𝔗}_{A_{{𝔥}_{𝔫-1}}},{𝔎}_{{𝔥}_{𝔫-1}},\;S_{{𝔪}_{𝔫-1}},{𝔗}_{{𝔪}_{𝔫-1}}\right) \end{array}\right]dȶ\\ {𝔸}_{\eta ,1}^{1}=\frac{\eta {𝕂}_{1}\left({ȶ}_{𝔫},\;{Ȿ}_{{𝔥}_{𝔫}},{𝔗}_{{Ȿ}_{{𝔥}_{𝔫}}},\;{\;𝔗}_{A_{{𝔥}_{𝔫}}},{𝔎}_{{𝔥}_{𝔫}},\;{Ȿ}_{{𝔪}_{𝔫}},{𝔗}_{{𝔪}_{𝔫}}\right)}{\mathrm{ABC}\left(\eta \right)\lceil \eta 𝔥}\\ \left[\int_{0}^{{ȶ}_{𝔫+1}}{\left({ȶ}_{𝔫+1}-t\right)}^{\eta -1}{𝕂}_{1}\left(ȶ-t_{𝔫-1}\right)\right]dȶ-\\ {𝕂}_{1}\left({ȶ}_{𝔫-1},\;{Ȿ}_{{𝔥}_{𝔫-1}},{𝔗}_{{Ȿ}_{{𝔥}_{𝔫-1}}},\;{\;𝔗}_{A_{{𝔥}_{𝔫-1}}},{𝔎}_{{𝔥}_{𝔫-1}},\;{Ȿ}_{{𝔪}_{𝔫-1}},{𝔗}_{{𝔪}_{𝔫-1}}\right)\\ \times \frac{\eta }{\mathrm{ABC}\left(\eta \right)\lceil \eta 𝔥}\\ \left[\int_{0}^{{ȶ}_{𝔫+1}}{\left({ȶ}_{𝔫+1}-t\right)}^{\eta -1}{𝕂}_{1}\left(ȶ-t_{𝔫-1}\right)\right]dȶ\;\\ {𝔸}_{\eta ,1}^{1}=\frac{\eta {𝕂}_{1}\left({ȶ}_{𝔫},\;{Ȿ}_{{𝔥}_{𝔫}},{𝔗}_{{Ȿ}_{{𝔥}_{𝔫}}},\;{\;𝔗}_{A_{{𝔥}_{𝔫}}},{𝔎}_{{𝔥}_{𝔫}},\;{Ȿ}_{{𝔪}_{𝔫}},{𝔗}_{{𝔪}_{𝔫}}\right)}{\mathrm{ABC}\left(\eta \right)\lceil \eta 𝔥}\left[\frac{2𝔥t_{𝔫+1}^{\eta }}{\eta }-\frac{{ȶ}_{𝔫+1}^{\eta +1}}{\eta +1}\right]-\;\\ {𝕂}_{1}\left({ȶ}_{𝔫-1},\;{Ȿ}_{{𝔥}_{𝔫-1}},{𝔗}_{{Ȿ}_{{𝔥}_{𝔫-1}}},\;{\;𝔗}_{A_{{𝔥}_{𝔫-1}}},{𝔎}_{{𝔥}_{𝔫-1}},\;{Ȿ}_{{𝔪}_{𝔫-1}},{𝔗}_{{𝔪}_{𝔫-1}}\right) \end{array}$$

(72) $$\begin{array}{l} \times \frac{\eta }{\mathrm{ABC}\left(\eta \right)\lceil \eta 𝔥}\left[\frac{𝔥t_{𝔫+1}^{\eta }}{\eta }-\frac{{ȶ}_{𝔫+1}^{\eta +1}}{\eta +1}\right]\; \end{array}$$

Similarly, for ${𝔸}_{\eta ,1}^{2}\ldots {𝔸}_{\eta ,1}^{6}$


$$\begin{array}{l} {𝔸}_{\eta ,1}^{2}=\frac{\eta {𝕂}_{2}\left({ȶ}_{𝔫},\;{Ȿ}_{{𝔥}_{𝔫}},{𝔗}_{{Ȿ}_{{𝔥}_{𝔫}}},\;{\;𝔗}_{A_{{𝔥}_{𝔫}}},{𝔎}_{{𝔥}_{𝔫}},\;{Ȿ}_{{𝔪}_{𝔫}},{𝔗}_{{𝔪}_{𝔫}}\right)}{\mathrm{ABC}\left(\eta \right)\lceil \eta 𝔥}\\ \left[\frac{2𝔥t_{𝔫+1}^{\eta }}{\eta }-\frac{{ȶ}_{𝔫+1}^{\eta +1}}{\eta +1}\right]\;-\\ {𝕂}_{2}\left({ȶ}_{𝔫-1},\;{Ȿ}_{{𝔥}_{𝔫-1}},{𝔗}_{{Ȿ}_{{𝔥}_{𝔫-1}}},\;{\;𝔗}_{A_{{𝔥}_{𝔫-1}}},{𝔎}_{{𝔥}_{𝔫-1}},\;{Ȿ}_{{𝔪}_{𝔫-1}},{𝔗}_{{𝔪}_{𝔫-1}}\right) \end{array}$$

(73) $$\begin{array}{l} \times \frac{\eta }{\mathrm{ABC}\left(\eta \right)\lceil \eta 𝔥}\left[\frac{𝔥t_{𝔫+1}^{\eta }}{\eta }-\frac{{ȶ}_{𝔫+1}^{\eta +1}}{\eta +1}\right] \end{array}$$

$$\begin{array}{l} {𝔸}_{\eta ,1}^{3}=\frac{\eta {𝕂}_{3}\left({ȶ}_{𝔫},\;{Ȿ}_{{𝔥}_{𝔫}},{𝔗}_{{Ȿ}_{{𝔥}_{𝔫}}},\;{\;𝔗}_{A_{{𝔥}_{𝔫}}},{𝔎}_{{𝔥}_{𝔫}},\;{Ȿ}_{{𝔪}_{𝔫}},{𝔗}_{{𝔪}_{𝔫}}\right)}{\mathrm{ABC}\left(\eta \right)\lceil \eta 𝔥}\\ \left[\frac{2𝔥t_{𝔫+1}^{\eta }}{\eta }-\frac{{ȶ}_{𝔫+1}^{\eta +1}}{\eta +1}\right]\;-\\ {𝕂}_{3}\left({ȶ}_{𝔫-1},\;{Ȿ}_{{𝔥}_{𝔫-1}},{𝔗}_{{Ȿ}_{{𝔥}_{𝔫-1}}},\;{\;𝔗}_{A_{{𝔥}_{𝔫-1}}},{𝔎}_{{𝔥}_{𝔫-1}},\;{Ȿ}_{{𝔪}_{𝔫-1}},{𝔗}_{{𝔪}_{𝔫-1}}\right) \end{array}$$

(74) $$\begin{array}{l} \times \frac{\eta }{\mathrm{ABC}\left(\eta \right)\lceil \eta 𝔥}\left[\frac{𝔥t_{𝔫+1}^{\eta }}{\eta }-\frac{{ȶ}_{𝔫+1}^{\eta +1}}{\eta +1}\right] \end{array}$$

$$\begin{array}{l} {𝔸}_{\eta ,1}^{4}=\frac{\eta {𝕂}_{4}\left({ȶ}_{𝔫},\;{Ȿ}_{{𝔥}_{𝔫}},{𝔗}_{{Ȿ}_{{𝔥}_{𝔫}}},\;{\;𝔗}_{A_{{𝔥}_{𝔫}}},{𝔎}_{{𝔥}_{𝔫}},\;{Ȿ}_{{𝔪}_{𝔫}},{𝔗}_{{𝔪}_{𝔫}}\right)}{\mathrm{ABC}\left(\eta \right)\lceil \eta 𝔥}\\ \left[\frac{2𝔥t_{𝔫+1}^{\eta }}{\eta }-\frac{{ȶ}_{𝔫+1}^{\eta +1}}{\eta +1}\right]\;-\\ {𝕂}_{4}\left({ȶ}_{𝔫-1},\;{Ȿ}_{{𝔥}_{𝔫-1}},{𝔗}_{{Ȿ}_{{𝔥}_{𝔫-1}}},\;{\;𝔗}_{A_{{𝔥}_{𝔫-1}}},{𝔎}_{{𝔥}_{𝔫-1}},\;{Ȿ}_{{𝔪}_{𝔫-1}},{𝔗}_{{𝔪}_{𝔫-1}}\right) \end{array}$$

(75) $$\begin{array}{l} \times \frac{\eta }{\mathrm{ABC}\left(\eta \right)\lceil \eta 𝔥}\left[\frac{𝔥t_{𝔫+1}^{\eta }}{\eta }-\frac{{ȶ}_{𝔫+1}^{\eta +1}}{\eta +1}\right] \end{array}$$

$$\begin{array}{l} {𝔸}_{\eta ,1}^{5}=\frac{\eta {𝕂}_{5}\left({ȶ}_{𝔫},\;{Ȿ}_{{𝔥}_{𝔫}},{𝔗}_{{Ȿ}_{{𝔥}_{𝔫}}},\;{\;𝔗}_{A_{{𝔥}_{𝔫}}},{𝔎}_{{𝔥}_{𝔫}},\;{Ȿ}_{{𝔪}_{𝔫}},{𝔗}_{{𝔪}_{𝔫}}\right)}{\mathrm{ABC}\left(\eta \right)\lceil \eta 𝔥}\\ \left[\frac{2𝔥t_{𝔫+1}^{\eta }}{\eta }-\frac{{ȶ}_{𝔫+1}^{\eta +1}}{\eta +1}\right]\;-\\ {𝕂}_{5}\left({ȶ}_{𝔫-1},\;{Ȿ}_{{𝔥}_{𝔫-1}},{𝔗}_{{Ȿ}_{{𝔥}_{𝔫-1}}},\;{\;𝔗}_{A_{{𝔥}_{𝔫-1}}},{𝔎}_{{𝔥}_{𝔫-1}},\;{Ȿ}_{{𝔪}_{𝔫-1}},{𝔗}_{{𝔪}_{𝔫-1}}\right) \end{array}$$

(76) $$\begin{array}{l} \times \frac{\eta }{\mathrm{ABC}\left(\eta \right)\lceil \eta 𝔥}\left[\frac{𝔥t_{𝔫+1}^{\eta }}{\eta }-\frac{{ȶ}_{𝔫+1}^{\eta +1}}{\eta +1}\right] \end{array}$$

$$\begin{array}{l} {𝔸}_{\eta ,1}^{6}=\frac{\eta {𝕂}_{6}\left({ȶ}_{𝔫},\;{Ȿ}_{{𝔥}_{𝔫}},{𝔗}_{{Ȿ}_{{𝔥}_{𝔫}}},\;{\;𝔗}_{A_{{𝔥}_{𝔫}}},{𝔎}_{{𝔥}_{𝔫}},\;{Ȿ}_{{𝔪}_{𝔫}},{𝔗}_{{𝔪}_{𝔫}}\right)}{\mathrm{ABC}\left(\eta \right)\lceil \eta 𝔥}\\ \left[\frac{2𝔥t_{𝔫+1}^{\eta }}{\eta }-\frac{{ȶ}_{𝔫+1}^{\eta +1}}{\eta +1}\right]\;-\\ {𝕂}_{6}\left({ȶ}_{𝔫-1},\;{Ȿ}_{{𝔥}_{𝔫-1}},{𝔗}_{{Ȿ}_{{𝔥}_{𝔫-1}}},\;{\;𝔗}_{A_{{𝔥}_{𝔫-1}}},{𝔎}_{{𝔥}_{𝔫-1}},\;{Ȿ}_{{𝔪}_{𝔫-1}},{𝔗}_{{𝔪}_{𝔫-1}}\right) \end{array}$$

(77) $$\begin{array}{l} \times \frac{\eta }{\mathrm{ABC}\left(\eta \right)\lceil \eta 𝔥}\left[\frac{𝔥t_{𝔫+1}^{\eta }}{\eta }-\frac{{ȶ}_{𝔫+1}^{\eta +1}}{\eta +1}\right] \end{array}$$

And, from ${𝔸}_{\eta ,2}^{1}$ to ${𝔸}_{\eta ,2}^{6}$ are given as


$$\begin{array}{l} {𝔸}_{\eta ,2}^{1}=\frac{\eta {𝕂}_{1}\left({ȶ}_{𝔫},\;{Ȿ}_{{𝔥}_{𝔫}},{𝔗}_{{Ȿ}_{{𝔥}_{𝔫}}},\;{\;𝔗}_{A_{{𝔥}_{𝔫}}},{𝔎}_{{𝔥}_{𝔫}},\;{Ȿ}_{{𝔪}_{𝔫}},{𝔗}_{{𝔪}_{𝔫}}\right)}{\mathrm{ABC}\left(\eta \right)\lceil \eta 𝔥}\\ \left[\frac{𝔥t_{𝔫}^{\eta }}{\eta }-\frac{{ȶ}_{𝔫}^{\eta +1}}{\eta +1}\right]-\;\\ {𝕂}_{1}\left({ȶ}_{𝔫-1},\;{Ȿ}_{{𝔥}_{𝔫-1}},{𝔗}_{{Ȿ}_{{𝔥}_{𝔫-1}}},\;{\;𝔗}_{A_{{𝔥}_{𝔫-1}}},{𝔎}_{{𝔥}_{𝔫-1}},\;{Ȿ}_{{𝔪}_{𝔫-1}},{𝔗}_{{𝔪}_{𝔫-1}}\right) \end{array}$$

(78) $$\begin{array}{l} \times \frac{1}{\mathrm{ABC}\left(\eta \right)\lceil \eta 𝔥}\; \end{array}$$

$$\begin{array}{l} {𝔸}_{\eta ,2}^{2}=\frac{\eta {𝕂}_{2}\left({ȶ}_{𝔫},\;{Ȿ}_{{𝔥}_{𝔫}},{𝔗}_{{Ȿ}_{{𝔥}_{𝔫}}},\;{\;𝔗}_{A_{{𝔥}_{𝔫}}},{𝔎}_{{𝔥}_{𝔫}},\;{Ȿ}_{{𝔪}_{𝔫}},{𝔗}_{{𝔪}_{𝔫}}\right)}{\mathrm{ABC}\left(\eta \right)\lceil \eta 𝔥}\\ \left[\frac{𝔥t_{𝔫}^{\eta }}{\eta }-\frac{{ȶ}_{𝔫}^{\eta +1}}{\eta +1}\right]\;-\\ {𝕂}_{2}\left({ȶ}_{𝔫-1},\;{Ȿ}_{{𝔥}_{𝔫-1}},{𝔗}_{{Ȿ}_{{𝔥}_{𝔫-1}}},\;{\;𝔗}_{A_{{𝔥}_{𝔫-1}}},{𝔎}_{{𝔥}_{𝔫-1}},\;{Ȿ}_{{𝔪}_{𝔫-1}},{𝔗}_{{𝔪}_{𝔫-1}}\right) \end{array}$$

(79) $$\begin{array}{l} \times \frac{1}{\mathrm{ABC}\left(\eta \right)\lceil \eta 𝔥}\; \end{array}$$

$$\begin{array}{l} {𝔸}_{\eta ,2}^{3}=\frac{\eta {𝕂}_{3}\left({ȶ}_{𝔫},\;{Ȿ}_{{𝔥}_{𝔫}},{𝔗}_{{Ȿ}_{{𝔥}_{𝔫}}},\;{\;𝔗}_{A_{{𝔥}_{𝔫}}},{𝔎}_{{𝔥}_{𝔫}},\;{Ȿ}_{{𝔪}_{𝔫}},{𝔗}_{{𝔪}_{𝔫}}\right)}{\mathrm{ABC}\left(\eta \right)\lceil \eta 𝔥}\\ \left[\frac{𝔥t_{𝔫}^{\eta }}{\eta }-\frac{{ȶ}_{𝔫}^{\eta +1}}{\eta +1}\right]\;-\\ {𝕂}_{3}\left({ȶ}_{𝔫-1},\;{Ȿ}_{{𝔥}_{𝔫-1}},{𝔗}_{{Ȿ}_{{𝔥}_{𝔫-1}}},\;{\;𝔗}_{A_{{𝔥}_{𝔫-1}}},{𝔎}_{{𝔥}_{𝔫-1}},\;{Ȿ}_{{𝔪}_{𝔫-1}},{𝔗}_{{𝔪}_{𝔫-1}}\right) \end{array}$$

(80) $$\begin{array}{l} \times \frac{1}{\mathrm{ABC}\left(\eta \right)\lceil \eta 𝔥}\; \end{array}$$

$$\begin{array}{l} {𝔸}_{\eta ,2}^{4}=\frac{\eta {𝕂}_{4}\left({ȶ}_{𝔫},\;{Ȿ}_{{𝔥}_{𝔫}},{𝔗}_{{Ȿ}_{{𝔥}_{𝔫}}},\;{\;𝔗}_{A_{{𝔥}_{𝔫}}},{𝔎}_{{𝔥}_{𝔫}},\;{Ȿ}_{{𝔪}_{𝔫}},{𝔗}_{{𝔪}_{𝔫}}\right)}{\mathrm{ABC}\left(\eta \right)\lceil \eta 𝔥}\\ \left[\frac{𝔥t_{𝔫}^{\eta }}{\eta }-\frac{{ȶ}_{𝔫}^{\eta +1}}{\eta +1}\right]\;-\\ {𝕂}_{4}\left({ȶ}_{𝔫-1},\;{Ȿ}_{{𝔥}_{𝔫-1}},{𝔗}_{{Ȿ}_{{𝔥}_{𝔫-1}}},\;{\;𝔗}_{A_{{𝔥}_{𝔫-1}}},{𝔎}_{{𝔥}_{𝔫-1}},\;{Ȿ}_{{𝔪}_{𝔫-1}},{𝔗}_{{𝔪}_{𝔫-1}}\right) \end{array}$$

(81) $$\begin{array}{l} \times \frac{1}{\mathrm{ABC}\left(\eta \right)\lceil \eta 𝔥}\; \end{array}$$

$$\begin{array}{l} {𝔸}_{\eta ,2}^{5}=\frac{\eta {𝕂}_{5}\left({ȶ}_{𝔫},\;{Ȿ}_{{𝔥}_{𝔫}},{𝔗}_{{Ȿ}_{{𝔥}_{𝔫}}},\;{\;𝔗}_{A_{{𝔥}_{𝔫}}},{𝔎}_{{𝔥}_{𝔫}},\;{Ȿ}_{{𝔪}_{𝔫}},{𝔗}_{{𝔪}_{𝔫}}\right)}{\mathrm{ABC}\left(\eta \right)\lceil \eta 𝔥}\\ \left[\frac{𝔥t_{𝔫}^{\eta }}{\eta }-\frac{{ȶ}_{𝔫}^{\eta +1}}{\eta +1}\right]\;-\\ {𝕂}_{5}\left({ȶ}_{𝔫-1},\;{Ȿ}_{{𝔥}_{𝔫-1}},{𝔗}_{{Ȿ}_{{𝔥}_{𝔫-1}}},\;{\;𝔗}_{A_{{𝔥}_{𝔫-1}}},{𝔎}_{{𝔥}_{𝔫-1}},\;{Ȿ}_{{𝔪}_{𝔫-1}},{𝔗}_{{𝔪}_{𝔫-1}}\right) \end{array}$$

(82) $$\begin{array}{l} \times \frac{1}{\mathrm{ABC}\left(\eta \right)\lceil \eta 𝔥}\; \end{array}$$

$$\begin{array}{l} {𝔸}_{\eta ,2}^{6}=\frac{\eta {𝕂}_{6}\left({ȶ}_{𝔫},\;{Ȿ}_{{𝔥}_{𝔫}},{𝔗}_{{Ȿ}_{{𝔥}_{𝔫}}},\;{\;𝔗}_{A_{{𝔥}_{𝔫}}},{𝔎}_{{𝔥}_{𝔫}},\;{Ȿ}_{{𝔪}_{𝔫}},{𝔗}_{{𝔪}_{𝔫}}\right)}{\mathrm{ABC}\left(\eta \right)\lceil \eta 𝔥}\\ \left[\frac{𝔥t_{𝔫}^{\eta }}{\eta }-\frac{{ȶ}_{𝔫}^{\eta +1}}{\eta +1}\right]\;-\\ {𝕂}_{6}\left({ȶ}_{𝔫-1},\;{Ȿ}_{{𝔥}_{𝔫-1}},{𝔗}_{{Ȿ}_{{𝔥}_{𝔫-1}}},\;{\;𝔗}_{A_{{𝔥}_{𝔫-1}}},{𝔎}_{{𝔥}_{𝔫-1}},\;{Ȿ}_{{𝔪}_{𝔫-1}},{𝔗}_{{𝔪}_{𝔫-1}}\right) \end{array}$$

(83) $$\begin{array}{l} \times \frac{1}{\mathrm{ABC}\left(\eta \right)\lceil \eta 𝔥}\; \end{array}$$

Finally, using Equations 72–77, and Equations 78–82 in Equations 53–58 therefore, we obtain the numerical solution of model (Equation 6), as a result


$$\begin{array}{l} {Ȿ}_{𝔥}\left({ȶ}_{𝔫+1}\right)=S_{𝔥}\left({ȶ}_{𝔫}\right)+\frac{1-\eta }{\mathrm{ABC}\left(\eta \right)}\\ \left[\begin{array}{c} {𝕂}_{1}\left({ȶ}_{𝔫},\;{Ȿ}_{{𝔥}_{𝔫}},{𝔗}_{{Ȿ}_{{𝔥}_{𝔫}}},\;{\;𝔗}_{A_{{𝔥}_{𝔫}}},{𝔎}_{{𝔥}_{𝔫}},\;{Ȿ}_{{𝔪}_{𝔫}},{𝔗}_{{𝔪}_{𝔫}}\right)-\\ {𝕂}_{1}\left({ȶ}_{𝔫-1},\;{Ȿ}_{{𝔥}_{𝔫-1}},{𝔗}_{{Ȿ}_{{𝔥}_{𝔫-1}}},\;{\;𝔗}_{A_{{𝔥}_{𝔫-1}}},{𝔎}_{{𝔥}_{𝔫-1}},\;S_{{𝔪}_{𝔫-1}},{𝔗}_{{𝔪}_{𝔫-1}}\right) \end{array}\right]\;\\ +\frac{\eta {𝕂}_{1}\left({ȶ}_{𝔫},\;{Ȿ}_{{𝔥}_{𝔫}},{𝔗}_{{Ȿ}_{{𝔥}_{𝔫}}},\;{\;𝔗}_{A_{{𝔥}_{𝔫}}},{𝔎}_{{𝔥}_{𝔫}},\;{Ȿ}_{{𝔪}_{𝔫}},{𝔗}_{{𝔪}_{𝔫}}\right)}{\mathrm{ABC}\left(\eta \right)\lceil \eta 𝔥}\left[\frac{2𝔥t_{𝔫+1}^{\eta }}{\eta }-\frac{{ȶ}_{𝔫+1}^{\eta +1}}{\eta +1}\right]\;\\ -{𝕂}_{1}\left({ȶ}_{𝔫-1},\;{Ȿ}_{{𝔥}_{𝔫-1}},{𝔗}_{{Ȿ}_{{𝔥}_{𝔫-1}}},\;{\;𝔗}_{A_{{𝔥}_{𝔫-1}}},{𝔎}_{{𝔥}_{𝔫-1}},\;{Ȿ}_{{𝔪}_{𝔫-1}},{𝔗}_{{𝔪}_{𝔫-1}}\right)\;\\ \times \frac{\eta }{\mathrm{ABC}\left(\eta \right)\lceil \eta 𝔥}\left[\frac{𝔥t_{𝔫+1}^{\eta }}{\eta }-\frac{{ȶ}_{𝔫+1}^{\eta +1}}{\eta +1}\right]\\ -\frac{\eta {𝕂}_{1}\left({ȶ}_{𝔫},\;{Ȿ}_{𝔥}\left({ȶ}_{𝔫}\right),{𝔗}_{S_{𝔥}}\left({ȶ}_{𝔫}\right),\;{\;𝔗}_{A_{𝔥}}\left({ȶ}_{𝔫}\right),{𝔎}_{𝔥}\left({ȶ}_{𝔫}\right),\;S_{𝔪}\left({ȶ}_{𝔫}\right),{𝔗}_{𝔪}\left({ȶ}_{𝔫}\right)\right)}{\mathrm{ABC}\left(\eta \right)\lceil \eta 𝔥}\\ \left[\frac{𝔥t_{𝔫}^{\eta }}{\eta }-\frac{{ȶ}_{𝔫}^{\eta +1}}{\eta +1}\right]\;-\\ {𝕂}_{1}\left({ȶ}_{𝔫-1},\;{Ȿ}_{{𝔥}_{𝔫-1}},{𝔗}_{{Ȿ}_{{𝔥}_{𝔫-1}}},\;{\;𝔗}_{A_{{𝔥}_{𝔫-1}}},{𝔎}_{{𝔥}_{𝔫-1}},\;{Ȿ}_{{𝔪}_{𝔫-1}},{𝔗}_{{𝔪}_{𝔫-1}}\right) \end{array}$$

(84) $$\begin{array}{l} \times \frac{1}{\mathrm{ABC}\left(\eta \right)\lceil \eta 𝔥}\; \end{array}$$

$$\begin{array}{l} {𝔗}_{{Ȿ}_{𝔥}}\left({ȶ}_{𝔫+1}\right)={𝔗}_{{Ȿ}_{𝔥}}\left({ȶ}_{𝔫}\right)+\frac{1-\eta }{\mathrm{ABC}\left(\eta \right)}\\ \left[\begin{array}{c} {𝕂}_{2}\left({ȶ}_{𝔫},\;{Ȿ}_{{𝔥}_{𝔫}},{𝔗}_{{Ȿ}_{{𝔥}_{𝔫}}},\;{\;𝔗}_{A_{{𝔥}_{𝔫}}},{𝔎}_{{𝔥}_{𝔫}},\;{Ȿ}_{{𝔪}_{𝔫}},{𝔗}_{{𝔪}_{𝔫}}\right)-\\ {𝕂}_{2}\left({ȶ}_{𝔫-1},\;{Ȿ}_{{𝔥}_{𝔫-1}},{𝔗}_{{Ȿ}_{{𝔥}_{𝔫-1}}},\;{\;𝔗}_{A_{{𝔥}_{𝔫-1}}},{𝔎}_{{𝔥}_{𝔫-1}},\;S_{{𝔪}_{𝔫-1}},{𝔗}_{{𝔪}_{𝔫-1}}\right) \end{array}\right]\;\\ +\frac{\eta {𝕂}_{2}\left({ȶ}_{𝔫},\;{Ȿ}_{{𝔥}_{𝔫}},{𝔗}_{{Ȿ}_{{𝔥}_{𝔫}}},\;{\;𝔗}_{A_{{𝔥}_{𝔫}}},{𝔎}_{{𝔥}_{𝔫}},\;{Ȿ}_{{𝔪}_{𝔫}},{𝔗}_{{𝔪}_{𝔫}}\right)}{\mathrm{ABC}\left(\eta \right)\lceil \eta 𝔥}\left[\frac{2𝔥t_{𝔫+1}^{\eta }}{\eta }-\frac{{ȶ}_{𝔫+1}^{\eta +1}}{\eta +1}\right]\\ -{𝕂}_{2}\left({ȶ}_{𝔫-1},\;{Ȿ}_{{𝔥}_{𝔫-1}},{𝔗}_{{Ȿ}_{{𝔥}_{𝔫-1}}},\;{\;𝔗}_{A_{{𝔥}_{𝔫-1}}},{𝔎}_{{𝔥}_{𝔫-1}},\;{Ȿ}_{{𝔪}_{𝔫-1}},{𝔗}_{{𝔪}_{𝔫-1}}\right)\\ \times \frac{\eta }{\mathrm{ABC}\left(\eta \right)\lceil \eta 𝔥}\left[\frac{𝔥t_{𝔫+1}^{\eta }}{\eta }-\frac{{ȶ}_{𝔫+1}^{\eta +1}}{\eta +1}\right]\;\\ -\frac{\eta {𝕂}_{2}\left({ȶ}_{𝔫},\;{Ȿ}_{{𝔥}_{𝔫}},{𝔗}_{{Ȿ}_{{𝔥}_{𝔫}}},\;{\;𝔗}_{A_{{𝔥}_{𝔫}}},{𝔎}_{{𝔥}_{𝔫}},\;{Ȿ}_{{𝔪}_{𝔫}},{𝔗}_{{𝔪}_{𝔫}}\right)}{\mathrm{ABC}\left(\eta \right)\lceil \eta 𝔥}\left[\frac{𝔥t_{𝔫}^{\eta }}{\eta }-\frac{{ȶ}_{𝔫}^{\eta +1}}{\eta +1}\right]\;-\;\\ {𝕂}_{2}\left({ȶ}_{𝔫-1},\;{Ȿ}_{{𝔥}_{𝔫-1}},{𝔗}_{{Ȿ}_{{𝔥}_{𝔫-1}}},\;{\;𝔗}_{A_{{𝔥}_{𝔫-1}}},{𝔎}_{{𝔥}_{𝔫-1}},\;{Ȿ}_{{𝔪}_{𝔫-1}},{𝔗}_{{𝔪}_{𝔫-1}}\right) \end{array}$$

(85) $$\begin{array}{l} \times \frac{1}{\mathrm{ABC}\left(\eta \right)\lceil \eta 𝔥}\; \end{array}$$

$$\begin{array}{l} {\;𝔗}_{A_{𝔥}}\left({ȶ}_{𝔫+1}\right)={\;𝔗}_{A_{𝔥}}\left({ȶ}_{𝔫}\right)+\frac{1-\eta }{\mathrm{ABC}\left(\eta \right)}\\ \left[\begin{array}{c} {𝕂}_{3}\left({ȶ}_{𝔫},\;{Ȿ}_{{𝔥}_{𝔫}},{𝔗}_{{Ȿ}_{{𝔥}_{𝔫}}},\;{\;𝔗}_{A_{{𝔥}_{𝔫}}},{𝔎}_{{𝔥}_{𝔫}},\;{Ȿ}_{{𝔪}_{𝔫}},{𝔗}_{{𝔪}_{𝔫}}\right)\\ -{𝕂}_{3}\left({ȶ}_{𝔫-1},\;{Ȿ}_{{𝔥}_{𝔫-1}},{𝔗}_{{Ȿ}_{{𝔥}_{𝔫-1}}},\;{\;𝔗}_{A_{{𝔥}_{𝔫-1}}},{𝔎}_{{𝔥}_{𝔫-1}},\;S_{{𝔪}_{𝔫-1}},{𝔗}_{{𝔪}_{𝔫-1}}\right) \end{array}\right]\;\\ +\frac{\eta {𝕂}_{3}\left({ȶ}_{𝔫},\;{Ȿ}_{{𝔥}_{𝔫}},{𝔗}_{{Ȿ}_{{𝔥}_{𝔫}}},\;{\;𝔗}_{A_{{𝔥}_{𝔫}}},{𝔎}_{{𝔥}_{𝔫}},\;{Ȿ}_{{𝔪}_{𝔫}},{𝔗}_{{𝔪}_{𝔫}}\right)}{\mathrm{ABC}\left(\eta \right)\lceil \eta 𝔥} \end{array}$$

$$\begin{array}{l} \left[\frac{2𝔥t_{𝔫+1}^{\eta }}{\eta }-\frac{{ȶ}_{𝔫+1}^{\eta +1}}{\eta +1}\right]\;-\\ {𝕂}_{3}\left({ȶ}_{𝔫-1},\;{Ȿ}_{{𝔥}_{𝔫-1}},{𝔗}_{{Ȿ}_{{𝔥}_{𝔫-1}}},\;{\;𝔗}_{A_{{𝔥}_{𝔫-1}}},{𝔎}_{{𝔥}_{𝔫-1}},\;{Ȿ}_{{𝔪}_{𝔫-1}},{𝔗}_{{𝔪}_{𝔫-1}}\right)\\ \times \frac{\eta }{\mathrm{ABC}\left(\eta \right)\lceil \eta 𝔥}\left[\frac{𝔥t_{𝔫+1}^{\eta }}{\eta }-\frac{{ȶ}_{𝔫+1}^{\eta +1}}{\eta +1}\right]\;\\ -\frac{\eta {𝕂}_{3}\left({ȶ}_{𝔫},\;{Ȿ}_{{𝔥}_{𝔫}},{𝔗}_{{Ȿ}_{{𝔥}_{𝔫}}},\;{\;𝔗}_{A_{{𝔥}_{𝔫}}},{𝔎}_{{𝔥}_{𝔫}},\;{Ȿ}_{{𝔪}_{𝔫}},{𝔗}_{{𝔪}_{𝔫}}\right)}{\mathrm{ABC}\left(\eta \right)\lceil \eta 𝔥}\left[\frac{𝔥t_{𝔫}^{\eta }}{\eta }-\frac{{ȶ}_{𝔫}^{\eta +1}}{\eta +1}\right]\;-\;\\ {𝕂}_{3}\left({ȶ}_{𝔫-1},\;{Ȿ}_{{𝔥}_{𝔫-1}},{𝔗}_{{Ȿ}_{{𝔥}_{𝔫-1}}},\;{\;𝔗}_{A_{{𝔥}_{𝔫-1}}},{𝔎}_{{𝔥}_{𝔫-1}},\;{Ȿ}_{{𝔪}_{𝔫-1}},{𝔗}_{{𝔪}_{𝔫-1}}\right) \end{array}$$

(86) $$\begin{array}{l} \times \frac{1}{\mathrm{ABC}\left(\eta \right)\lceil \eta 𝔥} \end{array}$$

$$\begin{array}{l} {𝔎}_{𝔥}\left({ȶ}_{𝔫+1}\right)={𝔎}_{𝔥}\left({ȶ}_{𝔫}\right)+\frac{1-\eta }{\mathrm{ABC}\left(\eta \right)}\\ \left[\begin{array}{c} {𝕂}_{4}\left({ȶ}_{𝔫},\;{Ȿ}_{{𝔥}_{𝔫}},{𝔗}_{{Ȿ}_{{𝔥}_{𝔫}}},\;{\;𝔗}_{A_{{𝔥}_{𝔫}}},{𝔎}_{{𝔥}_{𝔫}},\;{Ȿ}_{{𝔪}_{𝔫}},{𝔗}_{{𝔪}_{𝔫}}\right)\\ -{𝕂}_{4}\left({ȶ}_{𝔫-1},\;{Ȿ}_{{𝔥}_{𝔫-1}},{𝔗}_{{Ȿ}_{{𝔥}_{𝔫-1}}},\;{\;𝔗}_{A_{{𝔥}_{𝔫-1}}},{𝔎}_{{𝔥}_{𝔫-1}},\;S_{{𝔪}_{𝔫-1}},{𝔗}_{{𝔪}_{𝔫-1}}\right) \end{array}\right]\;\\ +\frac{\eta {𝕂}_{4}\left({ȶ}_{𝔫},\;{Ȿ}_{{𝔥}_{𝔫}},{𝔗}_{{Ȿ}_{{𝔥}_{𝔫}}},\;{\;𝔗}_{A_{{𝔥}_{𝔫}}},{𝔎}_{{𝔥}_{𝔫}},\;{Ȿ}_{{𝔪}_{𝔫}},{𝔗}_{{𝔪}_{𝔫}}\right)}{\mathrm{ABC}\left(\eta \right)\lceil \eta 𝔥}\left[\frac{2𝔥t_{𝔫+1}^{\eta }}{\eta }-\frac{{ȶ}_{𝔫+1}^{\eta +1}}{\eta +1}\right]\;-\;\\ {𝕂}_{4}\left({ȶ}_{𝔫-1},\;{Ȿ}_{{𝔥}_{𝔫-1}},{𝔗}_{{Ȿ}_{{𝔥}_{𝔫-1}}},\;{\;𝔗}_{A_{{𝔥}_{𝔫-1}}},{𝔎}_{{𝔥}_{𝔫-1}},\;{Ȿ}_{{𝔪}_{𝔫-1}},{𝔗}_{{𝔪}_{𝔫-1}}\right)\\ \times \frac{\eta }{\mathrm{ABC}\left(\eta \right)\lceil \eta 𝔥}\left[\frac{𝔥t_{𝔫+1}^{\eta }}{\eta }-\frac{{ȶ}_{𝔫+1}^{\eta +1}}{\eta +1}\right]\;\\ -\frac{\eta {𝕂}_{4}\left({ȶ}_{𝔫},\;{Ȿ}_{{𝔥}_{𝔫}},{𝔗}_{{Ȿ}_{{𝔥}_{𝔫}}},\;{\;𝔗}_{A_{{𝔥}_{𝔫}}},{𝔎}_{{𝔥}_{𝔫}},\;{Ȿ}_{{𝔪}_{𝔫}},{𝔗}_{{𝔪}_{𝔫}}\right)}{\mathrm{ABC}\left(\eta \right)\lceil \eta 𝔥}\left[\frac{𝔥t_{𝔫}^{\eta }}{\eta }-\frac{{ȶ}_{𝔫}^{\eta +1}}{\eta +1}\right]\;\\ -\;{𝕂}_{4}\left({ȶ}_{𝔫-1},\;{Ȿ}_{{𝔥}_{𝔫-1}},{𝔗}_{{Ȿ}_{{𝔥}_{𝔫-1}}},\;{\;𝔗}_{A_{{𝔥}_{𝔫-1}}},{𝔎}_{{𝔥}_{𝔫-1}},\;{Ȿ}_{{𝔪}_{𝔫-1}},{𝔗}_{{𝔪}_{𝔫-1}}\right) \end{array}$$

(87) $$\begin{array}{l} \times \frac{1}{\mathrm{ABC}\left(\eta \right)\lceil \eta 𝔥}\; \end{array}$$

$$\begin{array}{l} {Ȿ}_{𝔪}\left({ȶ}_{𝔫+1}\right)=S_{𝔪}\left({ȶ}_{𝔫}\right)+\frac{1-\eta }{\mathrm{ABC}\left(\eta \right)}\\ \left[\begin{array}{c} {𝕂}_{5}\left({ȶ}_{𝔫},\;{Ȿ}_{{𝔥}_{𝔫}},{𝔗}_{{Ȿ}_{{𝔥}_{𝔫}}},\;{\;𝔗}_{A_{{𝔥}_{𝔫}}},{𝔎}_{{𝔥}_{𝔫}},\;{Ȿ}_{{𝔪}_{𝔫}},{𝔗}_{{𝔪}_{𝔫}}\right)\\ -{𝕂}_{5}\left({ȶ}_{𝔫-1},\;{Ȿ}_{{𝔥}_{𝔫-1}},{𝔗}_{{Ȿ}_{{𝔥}_{𝔫-1}}},\;{\;𝔗}_{A_{{𝔥}_{𝔫-1}}},{𝔎}_{{𝔥}_{𝔫-1}},\;S_{{𝔪}_{𝔫-1}},{𝔗}_{{𝔪}_{𝔫-1}}\right) \end{array}\right]\;\\ +\frac{\eta {\;𝕂}_{5}\left({ȶ}_{𝔫},\;{Ȿ}_{{𝔥}_{𝔫}},{𝔗}_{{Ȿ}_{{𝔥}_{𝔫}}},\;{\;𝔗}_{A_{{𝔥}_{𝔫}}},{𝔎}_{{𝔥}_{𝔫}},\;{Ȿ}_{{𝔪}_{𝔫}},{𝔗}_{{𝔪}_{𝔫}}\right)}{\mathrm{ABC}\left(\eta \right)\lceil \eta 𝔥}\left[\frac{2𝔥t_{𝔫+1}^{\eta }}{\eta }-\frac{{ȶ}_{𝔫+1}^{\eta +1}}{\eta +1}\right]\\ -{𝕂}_{5}\left({ȶ}_{𝔫-1},\;{Ȿ}_{{𝔥}_{𝔫-1}},{𝔗}_{{Ȿ}_{{𝔥}_{𝔫-1}}},\;{\;𝔗}_{A_{{𝔥}_{𝔫-1}}},{𝔎}_{{𝔥}_{𝔫-1}},\;{Ȿ}_{{𝔪}_{𝔫-1}},{𝔗}_{{𝔪}_{𝔫-1}}\right)\\ \times \frac{\eta }{\mathrm{ABC}\left(\eta \right)\lceil \eta 𝔥}\left[\frac{𝔥t_{𝔫+1}^{\eta }}{\eta }-\frac{{ȶ}_{𝔫+1}^{\eta +1}}{\eta +1}\right]\;\\ -\frac{\eta {𝕂}_{5}\left({ȶ}_{𝔫},\;{Ȿ}_{{𝔥}_{𝔫}},{𝔗}_{{Ȿ}_{{𝔥}_{𝔫}}},\;{\;𝔗}_{A_{{𝔥}_{𝔫}}},{𝔎}_{{𝔥}_{𝔫}},\;{Ȿ}_{{𝔪}_{𝔫}},{𝔗}_{{𝔪}_{𝔫}}\right)}{\mathrm{ABC}\left(\eta \right)\lceil \eta 𝔥}\left[\frac{𝔥t_{𝔫}^{\eta }}{\eta }-\frac{{ȶ}_{𝔫}^{\eta +1}}{\eta +1}\right]\;\\ -{𝕂}_{5}\left({ȶ}_{𝔫-1},\;{Ȿ}_{{𝔥}_{𝔫-1}},{𝔗}_{{Ȿ}_{{𝔥}_{𝔫-1}}},\;{\;𝔗}_{A_{{𝔥}_{𝔫-1}}},{𝔎}_{{𝔥}_{𝔫-1}},\;{Ȿ}_{{𝔪}_{𝔫-1}},{𝔗}_{{𝔪}_{𝔫-1}}\right) \end{array}$$

(88) $$\begin{array}{l} \times \frac{1}{\mathrm{ABC}\left(\eta \right)\lceil \eta 𝔥}\; \end{array}$$

$$\begin{array}{l} {𝔗}_{𝔪}\left({ȶ}_{𝔫+1}\right)={𝔗}_{𝔪}\left({ȶ}_{𝔫}\right)+\frac{1-\eta }{\mathrm{ABC}\left(\eta \right)}\\ \left[\begin{array}{c} {𝕂}_{6}\left({ȶ}_{𝔫},\;{Ȿ}_{{𝔥}_{𝔫}},{𝔗}_{{Ȿ}_{{𝔥}_{𝔫}}},\;{\;𝔗}_{A_{{𝔥}_{𝔫}}},{𝔎}_{{𝔥}_{𝔫}},\;{Ȿ}_{{𝔪}_{𝔫}},{𝔗}_{{𝔪}_{𝔫}}\right)\\ -{𝕂}_{6}\left({ȶ}_{𝔫-1},\;{Ȿ}_{{𝔥}_{𝔫-1}},{𝔗}_{{Ȿ}_{{𝔥}_{𝔫-1}}},\;{\;𝔗}_{A_{{𝔥}_{𝔫-1}}},{𝔎}_{{𝔥}_{𝔫-1}},\;S_{{𝔪}_{𝔫-1}},{𝔗}_{{𝔪}_{𝔫-1}}\right) \end{array}\right]\;\\ +\frac{\eta K_{6}\left({ȶ}_{n},\;{Ȿ}_{h_{n}},T_{{Ȿ}_{h_{n}}},\;T_{A_{h_{n}}},K_{h_{n}},\;{Ȿ}_{m_{n}},T_{m_{n}}\right)}{AℬC\left(\eta \right)\lceil \eta \;h}\left[\frac{2ht_{n+1}^{\eta }}{\eta }-\frac{{ȶ}_{n+1}^{\eta +1}}{\eta +1}\right] \end{array}$$

(89) $$\begin{array}{l} {𝕂}_{6}\left({ȶ}_{n-1},\;{Ȿ}_{h_{n-1}},T_{{Ȿ}_{h_{n-1}}},\;T_{A_{h_{n-1}}},K_{h_{n-1}},\;{Ȿ}_{m_{n-1}},T_{m_{n-1}}\right)\\ \times \frac{\eta }{AℬC\left(\eta \right)\lceil \eta \;h}\left[\frac{ht_{n+1}^{\eta }}{\eta }-\frac{{ȶ}_{n+1}^{\eta +1}}{\eta +1}\right]\\ -\;\frac{\eta K_{6}\left({ȶ}_{n},\;{Ȿ}_{h_{n}},T_{{Ȿ}_{h_{n}}},\;T_{A_{h_{n}}},K_{h_{n}},\;{Ȿ}_{m_{n}},T_{m_{n}}\right)}{AℬC\left(\eta \right)\lceil \eta \;h}\left[\frac{ht_{n}^{\eta }}{\eta }-\frac{{ȶ}_{n}^{\eta +1}}{\eta +1}\right]\\ -K_{6}\left({ȶ}_{n-1},\;{Ȿ}_{h_{n-1}},T_{{Ȿ}_{h_{n-1}}},\;T_{A_{h_{n-1}}},K_{h_{n-1}},\;{Ȿ}_{m_{n-1}},T_{m_{n-1}}\right)\\ \times \frac{1}{AℬC\left(\eta \right)\lceil \eta \;h}\; \end{array}$$

### 6.1 Outcomes and results of simulation

In this section, we analyzed the dynamics of dengue disease spread using both fractional and non-fractional models across different compartments of the human and mosquito populations. Simulations based on dengue case data collected from Karnataka by NVBDC from August 2023 to May 2024, as detailed in reference (28), revealed notable differences in the behavior of each population compartment under fractional versus non-fractional conditions.

**Figure 2:**
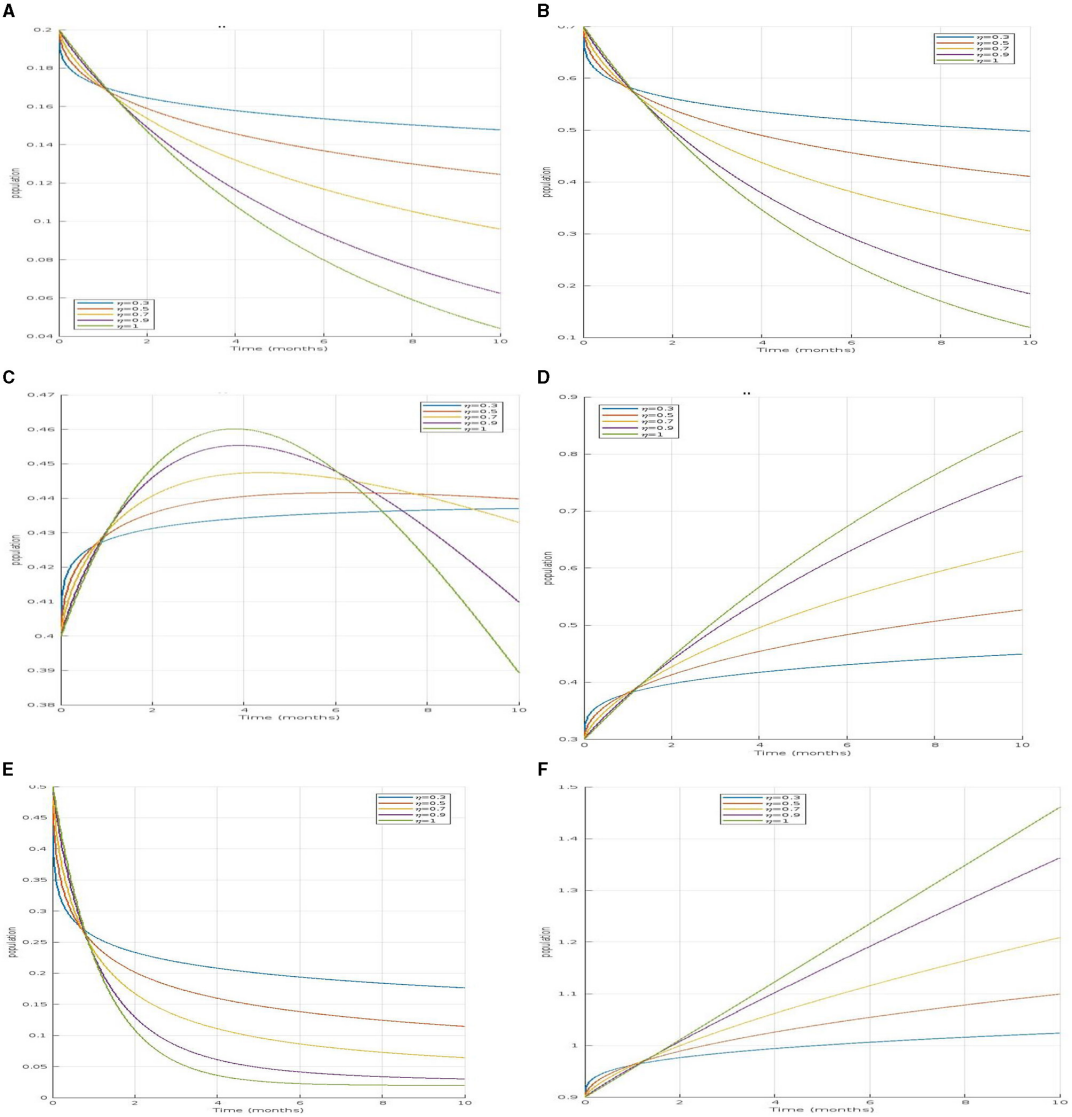
**(A–D)** Illustrate the time series of susceptible, infected, and recovered human populations for fractional orders and respectively. **(E, F)** Depict the time series of susceptible and infected mosquito populations for the same fractional orders. **(A)** Simulation of Ȿ_𝔥_ for different fractional order. **(B)** Simulation of $\;{\;𝔗}_{A_{𝔥}}$ for different fractional order. **(C)** Simulation of 𝔗_ℌ_𝔥__ for different fractional order. **(D)** Simulation of 𝔎_𝔥_ for different fractional order. **(E)** Simulation of Ȿ_𝔪_ for different fractional order. **(F)** Simulation of 𝔗_𝔪_ for different fractional order.

In Figure 2A, the susceptible population decreases with values of η = 0.3, 0.5, 0.7, and 0.9, indicating a more realistic and variable decline due to the complex interactions and memory effects incorporated. In contrast, the non-fractional model shows a constant value of η = 1, reflecting a simpler and less dynamic decrease. This suggests that the fractional model captures a more nuanced reduction in the susceptible population over time compared to the non-fractional approach. This suggests that the fractional model captures a more nuanced reduction in the susceptible population over time compared to the non-fractional approach.

For infected asymptomatic humans ${\;𝔗}_{A_{𝔥}}$ and infected symptomatic humans 𝔗_Ȿ_𝔥__, both populations will initially increase as the infection spreads but will eventually decrease as individuals recover or move between compartments in the Figures 2B, C.

Similarly, for the recovered human population 𝔎_𝔥_ in Figure 2D, the fractional model reflects a slower recovery rate, acknowledging the variability in recovery times, while the non-fractional model suggests a quicker recovery that might not align with real-world scenarios. In the mosquito populations, the susceptible mosquito population Ȿ_𝔪_ decrease in Figure 2E, more slowly in the fractional model, indicating that mosquitoes remain susceptible for longer periods. The infected mosquito population 𝔗_𝔪_ in Figure 2F, also rises gradually in the fractional model, unlike the rapid increase seen in the non-fractional model. Overall, the fractional models provide a more realistic representation of the disease dynamics by incorporating memory effects and delays, which better reflect the natural progression and spread of dengue compared to the more immediate transitions observed in non-fractional models.

The comparison clearly shows that fractional-order models provide a more nuanced understanding of how diseases like dengue evolve over time, influencing both human and mosquito populations. The ability of these models to incorporate memory effects allows them to better simulate the slow and cumulative impacts of disease control measures and environmental changes, offering a more realistic depiction of disease dynamics and aiding in the development of more effective intervention strategies.

Under this section, simulation results are performed, and values of specification are stated in Table 1.

**Table 1:** Description of parameter values.

**Symbols**	**Baseline values**
$\pi_{m}$	0.0071
$\pi_{h}$	0.057
$\delta_{h}$	0.00042
$\delta_{m}$	0.02
$\chi_{Sh}$	0.00567
$\chi_{Ah}$	0.01691
θ	0.025
$\alpha_{m}$	0.5
τ	0.03436
ϱ	0.40
γ	0.0947
$\chi_{m}$	0.0113

**Figure 3:**
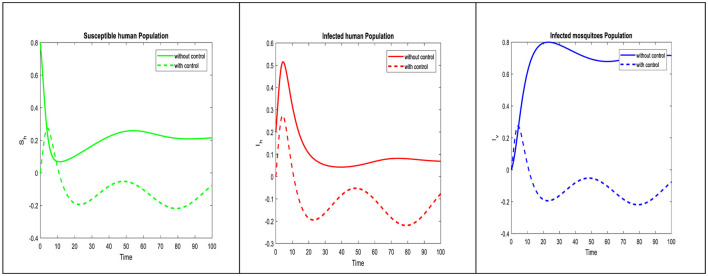
The influence of optimal control on the proposed model dynamics is illustrated.

The disease trajectory can be seen in Figure 3, when optimal control strategies are implemented and their effectiveness in reducing infection rates is highlighted. Comparing the two control strategies, it is evident that self-precautionary measures have a more immediate and direct effect on reducing human infection rates. This suggests that public education campaigns and community involvement can be impactful tools in controlling dengue viremia. Controlling both susceptible and infected mosquito populations is crucial for interrupting the disease transmission cycle. The impact of this strategy on reducing mosquito populations can be observed in the control diagram, illustrating the importance of vector management. Timing is critical for control strategies. The effect effectiveness of vector control may be contingent on seasonal variation in mosquito populations, while self-precaution can be promoted consistently. To maximize their impact, it is crucial to assess the optimal timing and deployment of these strategies.

## 7 Conclusions

The aim of this study is to explore the effect of dengue viremia on the occurrence of different illnesses. We have presented a comprehensive exploration of ABC fractional order Dengue viremia, a novel mathematical model that incorporates critical factors such as relapse and temporary immunity. After the model is created, the positivity and range of solution is evaluated, and the system survival and originality are verified. The basic reproduction value $R_{0}$ is determined by evaluating the equilibrium points. The Rough Hurwitz technique is commonly used to estimate local stability, while lyapunov functions are used to estimate global stability. Specifically, when $R_{0}<1$ in $C_{f},$ it indicates that the disease is unlikely to establish itself. If $R_{0}>1$ at ${\;C}_{p}$, it indicates that the disease is likely to continue to spread. Through the utilization of the Adams-Bash forth numerical scheme, we have successfully simulated disease dynamics, achieving a balance between computational efficiency and accuracy. In addition, we have developed the optimum measures by eradicating the population of mosquitoes and reducing the number of victims. The numerical simulation findings show the behavior of Dengue sickness model affected by different fractional orders, and they can serve as Dengue prevention and control recommendation. The research underscores the importance of mathematical modeling and optimal control techniques in addressing complex infectious disease like Dengue viremia. To develop interventions that reduce and control dengue, it is important to ensure that $R_{0}$ is below as a guideline. For future studies, our model can refine control strategies and adapt them to specific regions and epidemics, which is a promising way to treat infectious diseases and safeguard public health on a global scale.

## Data Availability

The raw data supporting the conclusions of this article will be made available by the authors, without undue reservation.
